# Induced pluripotent stem cell models of Zellweger spectrum disorder show impaired peroxisome assembly and cell type-specific lipid abnormalities

**DOI:** 10.1186/s13287-015-0149-3

**Published:** 2015-08-29

**Authors:** Xiao-Ming Wang, Wing Yan Yik, Peilin Zhang, Wange Lu, Ning Huang, Bo Ram Kim, Darryl Shibata, Madison Zitting, Robert H. Chow, Ann B. Moser, Steven J. Steinberg, Joseph G. Hacia

**Affiliations:** Department of Biochemistry and Molecular Biology, University of Southern California, Los Angeles, California USA; Department of Pathology, University of Southern California, Los Angeles, California USA; Department of Physiology and Biophysics, University of Southern California, Los Angeles, California USA; Hugo W. Moser Research Institute at Kennedy Krieger, Baltimore, Maryland USA

## Abstract

**Introduction:**

Zellweger spectrum disorder (PBD-ZSD) is a disease continuum caused by mutations in a subset of *PEX* genes required for normal peroxisome assembly and function. They highlight the importance of peroxisomes in the development and functions of the central nervous system, liver, and other organs. To date, the underlying bases for the cell-type specificity of disease are not fully elucidated.

**Methods:**

Primary skin fibroblasts from seven PBD-ZSD patients with biallelic *PEX1*, *PEX10*, *PEX12*, or *PEX26* mutations and three healthy donors were transduced with retroviral vectors expressing Yamanaka reprogramming factors. Candidate induced pluripotent stem cells (iPSCs) were subject to global gene expression, DNA methylation, copy number variation, genotyping, in vitro differentiation and teratoma formation assays. Confirmed iPSCs were differentiated into neural progenitor cells (NPCs), neurons, oligodendrocyte precursor cells (OPCs), and hepatocyte-like cell cultures with peroxisome assembly evaluated by microscopy. Saturated very long chain fatty acid (sVLCFA) and plasmalogen levels were determined in primary fibroblasts and their derivatives.

**Results:**

iPSCs were derived from seven PBD-ZSD patient-derived fibroblasts with mild to severe peroxisome assembly defects. Although patient and control skin fibroblasts had similar gene expression profiles, genes related to mitochondrial functions and organelle cross-talk were differentially expressed among corresponding iPSCs. Mitochondrial DNA levels were consistent among patient and control fibroblasts, but varied among all iPSCs. Relative to matching controls, sVLCFA levels were elevated in patient-derived fibroblasts, reduced in patient-derived iPSCs, and not significantly different in patient-derived NPCs. All cell types derived from donors with biallelic null mutations in a *PEX* gene showed plasmalogen deficiencies. Reporter gene assays compatible with high content screening (HCS) indicated patient-derived OPC and hepatocyte-like cell cultures had impaired peroxisome assembly.

**Conclusions:**

Normal peroxisome activity levels are not required for cellular reprogramming of skin fibroblasts. Patient iPSC gene expression profiles were consistent with hypotheses highlighting the role of altered mitochondrial activities and organelle cross-talk in PBD-ZSD pathogenesis. sVLCFA abnormalities dramatically differed among patient cell types, similar to observations made in iPSC models of X-linked adrenoleukodystrophy. We propose that iPSCs could assist investigations into the cell type-specificity of peroxisomal activities, toxicology studies, and in HCS for targeted therapies for peroxisome-related disorders.

**Electronic supplementary material:**

The online version of this article (doi:10.1186/s13287-015-0149-3) contains supplementary material, which is available to authorized users.

## Introduction

Peroxisomes are dynamic organelles that play critical roles in metabolic processes required for normal eukaryotic cell functions [1, 2]. The mammalian peroxisome proteome can vary according to the tissue, cell type, and physiological conditions [3]. Although the human peroxisomal proteome is not fully defined, at least 80 human proteins have been annotated as localizing to peroxisomes [4, 5]. In humans and other mammals, peroxisomal activities are responsible for the catabolism of branched chain and very long chain fatty acids, hydrogen peroxide byproducts of fatty acid oxidation, polyamines, certain amino acids, and glyoxylate [6]. In addition, they are required for the biosynthesis of ether-phospholipids, such as plasmalogens, platelet activating factor (PAF), and mature bile acids [6–8].

Zellweger spectrum disorder (PBD-ZSD) is a disease continuum consisting of Zellweger syndrome (ZS), neonatal adrenoleukodystrophy (NALD), and infantile Refsum disease (IRD), which are caused by biallelic defects in any of 14 *PEX* genes required for normal peroxisome assembly [9–11]. Individuals with ZS have profound intellectual disabilities secondary to neuronal migration defects and hypomyelination, hypotonia, liver dysfunction, and skeletal abnormalities, with survival up to 2 years of age [12, 13]. Nevertheless, the majority of PBD-ZSD patients have NALD and IRD, milder forms of disease that present after the newborn period [11]. These individuals typically show mild to moderate intellectual disabilities, craniofacial dysmorphism, liver dysfunction, progressive sensorineural hearing loss, retinopathy, and enamel hypoplasia [11, 14]. Individuals with IRD can survive into adulthood, as exemplified by a report in 2011 of a 28-year-old cognitively normal individual with IRD that manifested as severe visual and sensorineural hearing loss and enamel disease [15]. In general, disease severity is associated with the levels of residual *PEX* gene function [16].

Cultured skin fibroblasts from PBD-ZSD patients typically show defects in peroxisome assembly and metabolic functions [17]. As such, they provide a valuable platform for clinical diagnostics, studying the metabolic basis of disease, and screening for novel therapeutic agents [17, 18]. Nevertheless, cell type-specific differences in peroxisome morphology, number, protein composition, and metabolic activities, limit the ability of patient-derived cultured fibroblasts to model the specialized effects that peroxisome dysfunction have on other cell populations, such as those from central nervous system (CNS) and hepatic lineages, more relevant to PBD-ZSD pathology [3].

Here, we report the generation and characterization of patient-specific induced pluripotent stem cell (iPSC), CNS, and hepatocyte-like cell models of mild to severe PBD-ZSD. Gene expression profiles of patient-specific iPSCs, but not skin fibroblasts, reflected proposed pathomechanisms of disease highlighting cross-talk among multiple organelles. Furthermore, the variation in lipid abnormalities among patient cell types is consistent with cell type-specific peroxisomal activity levels. Collectively, our results suggest that iPSCs and their derivatives could provide valuable in vitro model systems to investigate molecular mechanisms and genetic and environmental modifiers relevant to peroxisome-related disorders in addition to screening for and evaluating targeted therapeutic interventions.

## Methods

### iPSC derivation

Primary dermal fibroblast cultures from PBD-ZSD patients and controls were obtained from the Kennedy Krieger Institute and Coriell Institute Cell Repositories (CIRC), respectively. We obtained Johns Hopkins University School of Medicine and University of Southern California Institutional Review Board approval for human subject research. HepG2 cells were purchased from CIRC. All the cells described herein were cultured at 37 °C with 5 % CO_2_. Human primary dermal fibroblasts and mitomycin C (Sigma-Aldrich) inactivated mouse embryonic fibroblasts (iMEFs) were cultured in fibroblast medium (DMEM with 10 % fetal bovine serum (FBS), L-glutamine, penicillin/streptomycin, vitamin solution, essential and nonessential amino acids (Life Technologies)), as described [19]. iPSCs were cultured on a layer of iMEFs in iPSC medium (DMEM:F12 medium with 20 % KSR, L-glutamine, penicillin/streptomycin, nonessential amino acids, β-mecaptoethanol and bFGF (Life Technologies)) as described [20–22].

Primary fibroblasts were transduced twice with a mixture of five retroviruses expressing the human *OCT4*, *SOX2*, *KLF4*, and *C-MYC* reprogramming factors and green fluorescent protein (GFP; to measure transduction efficiency) as described [20]. After 4 days, cells were trypsinized and re-plated on iMEF feeders and cultured in iPSC medium containing 1 mM valproic acid. By 4 weeks, candidate iPSC colonies were manually picked and clonally expanded. Confirmatory analyses were performed on multiple iPSC colonies from controls and PBD-ZSD patient donors, as described below and listed in Additional file 1.

### Immunostaining and differentiation assays

Alkaline phosphatase staining and immunostaining analysis using antibodies against OCT4, NANOG, SOX2, SSEA4, TRA-1-60, TuJ1, α-SMA, and AFP were performed as described [20]. Embryoid bodies (EBs) were produced from candidate iPSCs and subjected to in vitro differentiation assays, as described [20]. iPSCs were subcutaneously injected to the dorsal flanks of immunodeficient (SCID) mice to generate teratomas, which were excised and subjected to histological analysis, as described [20].

### Global gene expression profiling

Total RNA samples were processed, and analyzed on Affymetrix Human Genome 133A 2.0 or 133 Plus 2.0 GeneChips, as described [19]. The RMA algorithm was used to generate log2-transformed gene expression values, and conditional false discovery rates (FDRs) were determined by the spacings LOESS histogram (SPLOSH) method using the WebArray platform [23] (Additional file 2). We performed hierarchical clustering analysis using Partek Genomics Suite software, conducted GeneOntology (GO) and Kyoto Encyclopedia of Genes and Genomes (KEGG) pathway analyses using WebGestalt tools [24] and used Ingenuity Pathways Analysis (IPA) software (Ingenuity Systems) to analyze other functional relationships. Scaled gene expression scores and .cel files are available at the National Center for Biotechnology Information (NCBI) Gene Expression Omnibus (GEO) repository [25] under Series Accession Number GSE43996.

### Global genetic and epigenetic analysis

Human CytoSNP-12 Infinium HD BeadChips and GenomeStudio software (Illumina) were used for genome-wide single nucleotide polymorphism (SNP) genotyping and for data filtering and analysis, respectively. Copy number variation (CNV) analysis was performed using CNVPartition version 2.4.4 with a confidence threshold set at 50 and a minimum of 10 SNP probes per CNV region [26]. Specific *PEX* gene exons were sequenced using described protocols [27]. 450K Infinium Methylation BeadChips (Illumina) and GenomeStudio software were used for global DNA methylation analysis, as described [28, 29]. DNA methylation levels were summarized as β-values ranging from 0 (unmethylated) to 1 (fully methylated). Scaled DNA methylation scores and .idat files are available at the NCBI GEO repository [25] under Series Accession Number GSE68134. Confirmatory bisulfite DNA sequencing was conducted as described [28, 29].

### mtDNA analysis

The NovaQUANT Human Mitochondrial to Nuclear DNA Ratio Kit (EMD Millipore, Darmstadt, Germany) was used to estimate mtDNA levels in select control and PBD-ZSD patient-derived fibroblasts and iPSCs according to manufacturer’s instructions. All mtDNA measurements were performed in triplicate. Controls provided by the manufacturers included total DNA isolated from human 143B cells and human 143B rho zero cells. As previously discussed [30], rho zero cells are devoid of mtDNA.

### Lipid analysis

As previously described [20], we evaluated relative saturated very long chain fatty acid (sVLCFA) levels in cell lysates by determining the ratio of C26:0-lysophosphorylcholine (C26:LPC) and C22:0-lysophosphorylcholine (C22:LPC) levels (i.e., C26:0LPC/C22:0LPC) by liquid chromatography–tandem mass spectrometry (LC-MS/MS). We report %C26:0LPC as being relative to the total amount of all lysophosphatidylcholine molecular species (C26:0, C24:0, C22:0, C20:0, C18:0, C18:1, and C18:2 LPCs) and lyso-platelet activating factor molecular species (C16:0-Lyso-PAF, and 1-C18:0-Lyso-PAF) determined in the same LC-MS/MS analysis. All values are provided in Additional file 3. We also used liquid LC-MS/MS to measure 16:0p/20:4, 16:0p/18:1, 18:1p/20:4, 18:0p/20:4 phosphatidylethanolamine (PE) plasmalogen levels in cell lysates as described in [31]. The ratios of total PE plasmalogens to total LPC are provided in Additional file 3.

### CNS lineage differentiation

EBs were formed and maintained as described [20] for 4 days, then switched to neural induction medium (NIM) containing DMEM:F12 medium with 1 % N2, L-glutamine, penicillin/streptomycin, nonessential amino acids, and 2 μg/ml heparin (Life Technologies) for 3 days. On day 7, EBs were attached to Matrigel-coated (BD Biosciences) cell culture plates and maintained in the same medium for an extra 7 to 10 days for neural epithelia (NE) induction. Small columnar-like neural rosette structures of NE appeared around day 10 [32, 33].

To initiate motor neuron differentiation, 1 μM retinoic acid (RA) was added to NIM at day 10. After 5 more days, the NE rosettes were gently blown off by a 1-ml pipette and triturated to form motor neuron progenitors/neural spheres by culturing in neural differentiation medium (NDM) (DMEM:F12 with 1 % N2, 2 % B27, L-glutamine, penicillin/streptomycin, nonessential amino acids, 2 μg/ml heparin, 1 μM RA and 100 ng/ml sonic hedgehog (SHH)) for about 1 month (Life Technologies)). For terminal motor neuron differentiation, neural spheres were triturated and attached to laminin-coated cell culture plates and maintained in NDM with 1 μM cAMP, 200 μg/ml ascorbic acid and neurotrophic factors (10 ng/ml each of BDNF, GDNF and IGF1) for up to 7 weeks [32, 33].

To initiate oligodendrocyte differentiation, iPSCs were detached and resuspended in transition medium containing 50 % iPSC medium and 50 % glial restrictive medium (GRM) (DMEM:F12, 2 % B27 (Invitrogen), 25 μg/ml insulin, 6.3 ng/ml progesterone, 10 μg/ml putrescine, 50 ng/ml sodium selenite, 50 μg/ml holotransferrin, and 40 ng/ml triiodothyronine (Sigma)) with 5 ng/ml fibroblast growth factor (FGF2) and 20 ng/ml endothelial growth factor (EGF) in low-adherent plates for 2 days. On day 3, unattached EBs were switched to GRM supplemented with 20 ng/ml EGF and 5 μM RA for 8 days with daily media changes. On day 11, yellow spheres were selected, cut to smaller pieces and maintained in GRM supplemented with 20 ng/ml EGF. On day 28, yellow spheres were again cut to smaller pieces and attached to 1:30 diluted growth-factor-reduced Matrigel (BD Biosciences) in the same medium. After 1 week, the attached cell clusters were dissociated by incubating in 1x HBSS for 10–15 minutes and attached to poly-L-ornithine/fibronectin double-coated plates. To expand oligodendrocyte progenitors (OPs), cells were maintained in GRM supplemented with 1 % N2, 10 ng/ml FGF2 and 20 ng/ml EGF (Life Technologies) for 10 days, then switched to GRM supplemented with PDGF-AA (R&D systems), IGF1 (Peprotech), biotin, and cAMP (Sigma-Aldrich). To obtain terminally differentiated oligodendrocytes (OLs), cells were maintained in GRM supplemented with 1 % N2, 50 ng/ml noggin (R&D systems), 5 ng/ml FGF2 and 10 ng/ml EGF for 2–3 days. Afterwards, FGF2 and EGF were removed from the medium along with the addition of 1 mM cAMP, 200 nM ascorbic acid (Sigma-Aldrich), 20 ng/ml IGF, GDNF, and CNTF (Peprotech) [34–38].

### Hepatocyte lineage differentiation

Hepatic cell lineages were derived following the protocol of Duan et al. [39, 40] with modifications. Briefly, iPSCs cultured on matrigel-coated plates with MEF-conditioned medium were induced into definitive endoderm by switching to serum-free RPMI 1640 medium (Life Tech) supplemented with 100 ng/ml Activin A (Peprotech), 2 mM L-glutamine, and 1 % antibiotic-antimycotic for 48 hours. This medium was supplemented with 1 × B27 supplement (Life Tech) and 0.5 mM sodium butyrate (Sigma Aldrich) for the next 3–6 days. To initiate differentiation, we treated definitive endodermal cells with 20 ng/ml FGF4, 20 ng/ml bone morphogenic protein 2 (BMP2) and 20 ng/ml hepatocyte growth factor (HGF) (Peprotech) in Iscove’s modified Dulbecco’s medium (Gibco) with 20 % FBS, 2 mM L-glutamine, 0.3 mM monothioglycerol (Sigma Aldrich), 1 % antibiotic-antimycotic, 1 μM insulin (Gibco), 0.5 % DMSO and 100 nM dexamethosome (Sigma-Aldrich) for 10 days. For maturation, we cultured these cells in hepatocyte culture medium supplemented with SingleQuots (Lonza Walkerville) with 2 % FBS, 20 ng/ml FGF4, 20 ng/ml hepatocyte growth factor (HGF), 50 ng/ml oncostatin M (R&D Systems), 100 nM dexamethasone and 0.5 % DMSO for 6 to 10 days.

### Characterization of hepatocyte-like cell cultures

Immunostaining was conducted using the following antibodies: anti-albumin (Thermo Scientific #RB-1925-R2), anti-AFP (Life Tech #18-0055), anti-HNF4a (Santa Cruz #sc-6556), anti-CYP3A (L-14) (Santa Cruz #sc-30621), anti-ASGPR (Santa Cruz #sc-13467) and appropriate FITC- or rhodamine-conjugated secondary antibodies. Flow cytometry analysis was performed using the FACS LSRII machine (BD Biosciences). Cells in suspension were fixed in 2 % paraformaldehyde for 15 minutes and stained with primary antibodies against ASGPR in 5 % normal donkey serum at room temperature for 30 minutes followed by secondary antibodies for 20 minutes. Control samples were stained with the corresponding IgG only.

Total RNA samples from flow-sorted ASGPR-positive candidate hepatocyte-like cells obtained from healthy (control1) and PBD-ZSD patient (PBD_PEX1ms1 and PBD_PEX1fs1) donors were processed and analyzed on Affymetrix Human Genome 133A 2.0 microarrays as described in the “Global gene expression profiling” section above. Scaled gene expression scores are provided in Additional file 4. In addition, scaled gene expression scores and .cel files are available at the NCBI GEO repository [25] under Series Accession Number GSE69066.

Glycogen storage was evaluated using the Periodic Acid-Schiff (PAS) Kit (Sigma-Aldrich). The amount of urea secreted into the cell culture medium was quantified using the QuantiChrom Urea Assay Kit (BioAssay Systems). The amount of human albumin secreted in the supernatant was determined by the AssayMax Human Albumin ELISA Kit (Assaypro LLC). Urea production and albumin levels were normalized to total cell numbers, determined by counting trypsinized cells with a hemocytometer.

## Results

### Derivation of iPSCs from control and PBD-ZSD patient fibroblasts

**Fig. 1:**
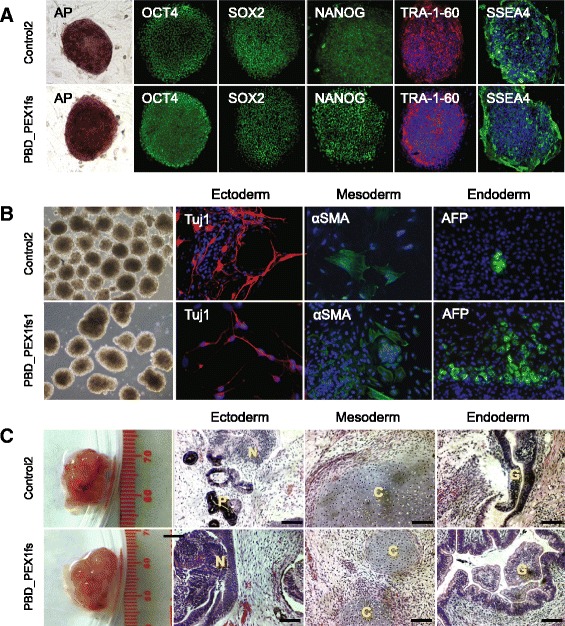
Characterization of iPSCs derived from PBD-ZSD patient and healthy control donors. **a** Alkaline phosphatase (AP) staining and immunostaining for pluripotency markers in representative healthy control and patient iPSCs. **b** Immunostaining for cell populations derived from each of the three germ layers based on in vitro EB differentiation assays conducted on representative healthy control and patient iPSCs. **c** Teratomas derived from representative healthy control and patient iPSCs. Teratomas consisted of cell populations representative of all three germ layers. Scale bar = 50 μm. *N* neural rosettes; *P* pigment epithelium; *C* cartilage tissue; *G* glandular tissue

Cultured primary skin fibroblasts from three healthy control and seven PBD-ZSD patient donors with mutations in the *PEX1*, *PEX10*, *PEX12*, or *PEX26* genes were transduced with retroviruses expressing the human *OCT4*, *SOX2*, *KLF4*, and *c-MYC* reprogramming factors (Table 1). Patient and control fibroblasts produced iPSC-like colonies by 2 weeks and TRA-1-60- or TRA-1-81-positive colonies were clonally expanded after 1 month. We characterized multiple candidate iPSCs from control and patients, all showing the appropriate morphology and positive immunostaining for pluripotency markers (Fig. 1a; Additional file 5). All control and PBD-ZSD patient-derived candidate iPSCs could be differentiated in vitro into cells derived from all three germ layers (Fig. 1b). All three candidate iPSCs (one control and two patient-derived) injected into immune-deficient mice produced teratomas with tissue representative of all three germ layers (Fig. 1c).

**Table 1:** Skin fibroblast donor information

Current ID	Prior ID	Description	*PEX* gene mutations	*PEX* gene notes
Control1	AG05838	Healthy female, 36 years	Presumed wild type	None
Control2	AG09599	Healthy female, 30 years	Presumed wild type	None
Control3	AG13153	Healthy male, 30 years	Presumed wild type	None
PBD_PEX1fs1	PBD721	PBD-ZSD patient	*PEX1* c.2097_2098insT p.I700fs; c.2916delA p.G973fs	Two null alleles
PBD_PEX1fs2	PBD702	PBD-ZSD patient	*PEX1* c.2097_2098insT p.I700fs; 2916delA p.G973fs	Two null alleles
PBD_PEX1ms1	PBD615	PBD-ZSD patient	Homozygous *PEX1* c.2528G>A p.G843D	Hypomorphic alleles^a^
PBD_PEX1ms2	PBD643	PBD-ZSD patient	Homozygous *PEX1* c.2528G>A p.G843D	Hypomorphic alleles^a^
PBD_PEX10	PBD687	PBD-ZSD patient	*PEX10* c.337delC p. L113fs; c.890T>C p.L297P	Null allele and hypomorphic allele^b^
PBD_PEX12^c^	PBD673	PBD-ZSD patient	Homozygous *PEX12* c.959C>T p.S320F	Hypomorphic alleles^c^
PBD_PEX26	PBD604	PBD-ZSD patient	Homozygous *PEX26* c.292 C>T p.R98W	Hypomorphic alleles^d^

Prior ID number for control cell lines obtained from Coriell Cell Repositories and PBD-ZSD patient cells obtained from the Kennedy Krieger Institute are provided

^a^Skin fibroblasts derived from multiple patients with this genotype have a temperature-sensitive peroxisome assembly defect [52]

^b^Skin fibroblasts derived from this patient have relative sVLCFA levels in the normal range under standard growth conditions [53]. *PEX1* c.880A>G p.T294A allele of unknown significance is also present

^c^Skin fibroblasts derived from multiple patients with this genotype have relative sVLCFA levels in the normal range under standard growth conditions [54]

^d^Skin fibroblasts derived from multiple patients with this genotype have a temperature-sensitive peroxisome assembly defect [55]

*PBD-ZSD* Zellweger spectrum disorder, *sVLCFA* saturated very long chain fatty acid

In all cases, skin fibroblasts and iPSCs derived from the same donor had at least 99.9 % concordant SNP genotypes, based on BeadArray data. From these analyses, we did not detect copy number changes (CNCs; i.e., insertions or deletions >10 kb in length) in five PBD-ZSD patient and four control iPSCs (Additional file 6). Consistent with prior reports involving reprogrammed human cells [41–43], we detected CNCs in 19/28 (68 %) of the iPSCs analyzed (Additional file 6). Patient iPSCs retained the expected *PEX* gene mutations and control iPSCs lacked these specific mutations.

### Global gene expression and DNA methylation profiles of patient and control-derived cells

**Fig. 2:**
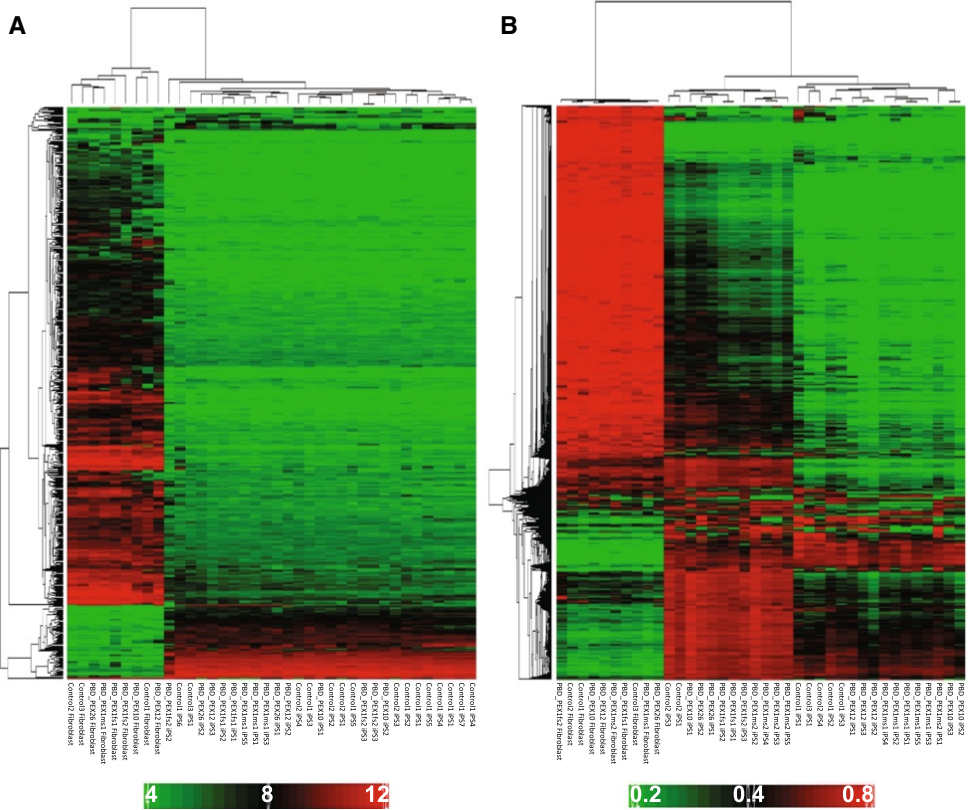
Gene expression and epigenetic profiles of fibroblasts and iPSCs. **a** Dendrogram depicting unsupervised hierarchical clustering analysis of gene expression data from PBD-ZSD patient and healthy control skin fibroblasts and iPSCs. Analysis was based on log2-transformed gene expression scores from 575 probe sets with coefficient of variation (CV) greater than 0.25 and conducted using average linkage and Euclidean distance. Color bar represents log-2 transformed gene expression values. **b** Dendrogram depicting results of unsupervised hierarchical clustering analysis of DNA methylation data from PBD-ZSD patient and healthy control skin fibroblasts and iPSCs. Analysis was based on 4073 DNA methylation assays interrogating autosomal CpG loci with CV greater than 0.75 and the 10th largest and smallest β-value being greater than 0.6 and less than 0.4, respectively, in order to represent the most variable loci. Clustering was performed using average linkage and Pearson dissimilarity distance. Color bar represents β-values

We measured the expression levels of over 18,000 transcripts in iPSCs and skin fibroblasts using microarrays. Unsupervised hierarchical clustering analyses based on expression data from the most variably expressed transcripts (Fig. 2a) or iPSC signature genes [44] yielded two distinct sample clusters comprised entirely of either skin fibroblast or iPSC samples. In both clusters (Fig. 2a and Additional file 7), the samples grouped independent of donor health with robust expression of iPSC signature genes in all candidate iPSCs analyzed, but not in skin fibroblasts. We also examined the DNA methylation levels of over 485,000 CpG sites in genomic DNAs (gDNAs) from all the fibroblast cultures and iPSCs listed in Table 1. Hierarchical clustering analysis based on DNA methylation data from the most highly variable loci produced two distinct clusters that clearly separated the skin fibroblast and iPSC gDNA samples, again independent of donor health (Fig. 2b).

### Differentially expressed genes in patient iPSCs reflect proposed pathogenic mechanisms

No differentially expressed genes (DEGs) (>1.2-fold change, FDR <0.05) were uncovered based on global gene expression analysis of skin fibroblasts derived from six PBD-ZSD patient and three healthy control donors. In contrast to these observations in fibroblasts, we identified 132 DEGs (79 with higher and 53 with lower expression in PBD-ZSD relative to control cells) based on global gene expression profiles of 13 iPSCs from six PBD-ZSD patient donors and 12 iPSCs from three healthy control donors (Additional file 8A). GO analysis yielded enrichment for “Cellular Component” with categories that included the endoplasmic reticulum (18 genes), mitochondrion (19 genes), and golgi apparatus (16 genes) (Additional file 9A). Enriched KEGG pathways relevant to peroxisome biology included ‘Fatty Acid Metabolism’ (Additional file 9D). IPA pathway analyses highlighted 87 enriched categories that were mostly broadly defined with two related to carbohydrate metabolism (‘synthesis of carbohydrates’ and ‘uptake of monosaccharide’) and one related to lipid metabolism (‘accumulation of lipid’) (Additional file 10E).

Pathway analysis was also conducted on DEGS based on whether they showed higher expression in PBD-ZSD patient or control iPSCs. This revealed that most of the mitochondrial DEGs (13 of 19) and all three HLA-related DEGS (HLA-E, HLA-F, and HLA-G) showed higher expression in the peroxisome-deficient patient iPSCs (Additional file 9B, D, E). IPA pathway analysis highlighted 58 and 64 categories of DEGS with higher expression in patient-derived and control iPSCs, respectively. The former included ‘accumulation of lipid’ while the latter included ‘synthesis of carbohydrates’ and ‘uptake of monosaccharide’ (Additional file 9E).

We also annotated probe tilings and manually searched for DEGs relevant to peroxisome biology using the DAVID Bioinformatics resource. Although none encoded strictly peroxisomal proteins, multiple DEGs were directly or indirectly related to peroxisomal activities. For example, *ATG12* (higher in patient iPSCs) and *KIAA0652* (also known as *ATG13*) (higher in control iPSCs) are relevant to pexophagy, the autophagic degradation of peroxisomes [45]. PBD-ZSD patient cells can have glycosylphosphatidylinositol (GPI) lipid remodeling defects, which results in the absence of the 1-alkyl-2-acyl form of GPI-anchored proteins on their surface [46, 47]. Control iPSCs had higher expression of the *PIGL* and *PGAP2* genes critical for GPI-anchored protein biosynthesis while patient iPSCs had higher expression of the *THY1* and *FOLR1* genes that encode GPI anchor proteins. DEGS relevant to biochemical activities either directly or indirectly relevant to peroxisomes included *ELOV5*, *ACAT1*, *ALDH3A2*, *CPT1A*, *LIPA*, and *NUDT4* (fatty acid metabolism), *CLN8*, *LIPA*, and *OSBL2* (cholesterol metabolism), and *ACTAT1* (branched chain amino acid metabolism). Splice variants of the differentially expressed *ALDH3A2* gene can yield peroxisomal or endoplasmic reticulum (ER) proteins [48].

### Instability of *PEX1*-mutated transcripts predicted to be subject to nonsense-mediated decay

As an internal control to check our sensitivity to detect DEGs related to peroxisome function, we compared the gene expression profiles of nine iPSC colonies derived from all three control donors and four iPSC colonies derived from PBD_PEX1fs1 and PBD_PEX1fs2. These PBD-ZSD patient-derived colonies should only produce unstable *PEX1* transcripts due to nonsense-mediated decay. Only 13 DEGs were found in this comparison (Additional file 8B). In keeping with expectations, *PEX1* was among the DEGs that showed reduced expression in the patient cells. Due to our limited statistical power in this subset analysis, the numbers of DEGs were too limited to conduct meaningful pathway enrichment analysis. Nevertheless, we note that the aforementioned *ALDH3A2* gene was a DEG in this analysis.

### mtDNA copy number is consistent among fibroblasts but varies in iPSCs

**Fig. 3:**
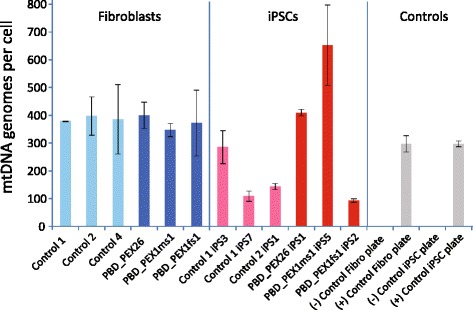
Estimated mtDNA genome copy number in PBD-ZSD patient and healthy control-derived cells. The numbers of mtDNA genomes per cell were estimated by quantitative PCR in triplicate. Total DNA isolated from human 143B cells and human 143B rho cells (devoid of mtDNA) served as positive (+) and negative (−) controls. Fibroblast and iPSC DNA samples were analyzed on different 96-well plates with embedded positive controls (*grey*) yielding similar results (330 and 267 mtDNA genomes per cell, respectively). All data from the fibroblast (*light blue*: controls; *dark blue*: patients) and iPSC (*light red*: controls; *dark red*: patients) plates were normalized so that internal positive control provided an estimate of 297 mtDNA genomes per cell (average of mtDNA estimates above). Error bars represent the high and low estimates of mtDNA genomes per cell in a given sample. Fibroblast control4 is derived from fibroblast culture AG04446 obtained from a 48-year-old healthy male donor (Coriell Cell Repositories). *iPSC* induced pluripotent stem cell

To further examine the effects of impaired peroxisome assembly on mitochondrial biology, we estimated mtDNA genome copy number per diploid nuclear genome in fibroblasts and iPSCs from patients and controls (Fig. 3). Normalized estimates of mtDNA genome copy number were consistent, all within 1.2-fold of one another. In contrast, mtDNA genome copy number estimates varied up to 7.1-fold among iPSCs. Moreover, mtDNA genome copy numbers varied 2.6-fold between two different iPSC colonies derived from healthy donor control1 (Fig. 3).

### Derivation and characterization of CNS cell lineages from control and PBD-ZSD patient fibroblasts

Neural progenitor inductions were performed on six control and 17 patient iPSC colonies (Additional file 10). To estimate in vitro neural differentiation efficiencies, we calculated the percentage of attached EBs that formed neural rosettes. Excluding data from the *PEX10* and *PEX12* mutant iPSC colonies derived from patient fibroblasts with relative sVLCFA levels in the normal range (Additional file 3, discussed in the next section), the cohort of patient EBs had a 1.8-fold reduced neural differentiation efficiency relative to control-derived EBs (*P* = 0.028) (Additional file 10A). However, there was no significant difference in the average rates of neural rosette formation from patient- and control-derived EBs, all ranging from 9 to 13 days (Additional file 10B).

**Fig. 4:**
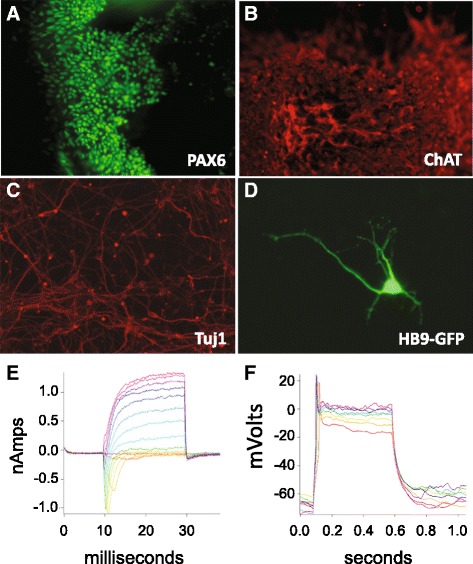
Neural differentiation of PBD-ZSD-derived iPSCs. PDB_PEX1ms1 patient-derived iPSCs were immunostained and imaged during the process of differentiation into motor neurons. Images depicting the expression of **a** PAX6 in the neural progenitor cells, **b** ChAT in neurons, **c** TuJ1 in neurons, and **d** GFP transgene under the control of the *HB9* enhancer in transfected motor neurons. **e, f** Whole cell patch clamp recording from candidate neurons derived from PBD-ZSD patient PBD_PEX1fs1 iPSCs. **e** Voltage-clamp records at a single patient cell, showing currents recorded at different voltages. The shapes of the curves are typical for a cell expressing sodium and potassium channels. Holding potential was −70 mV and the voltages of the steps range from −60 mV up to +60 mV, in 10-mV steps. **f** Current-clamp records at a single patient cell in response to current injection. Current injection triggers action potential firing, as expected for neurons

In preliminary studies, we demonstrated that patient and control iPSCs could be differentiated into Tuj1-positive neurons and HB9-expressing candidate motor neurons. Representative immunostaining images acquired during the neural differentiation process are provided in Fig. 4 and Additional file 11. There were no obvious morphological differences among the patient- and control-derived neurons. Patch clamp analysis of a neuron derived from patient PBD_PEX1fs1 demonstrated electrophysiological characteristics indicative of active sodium channels and potassium channels (Fig. 4e, f).

**Fig. 5:**
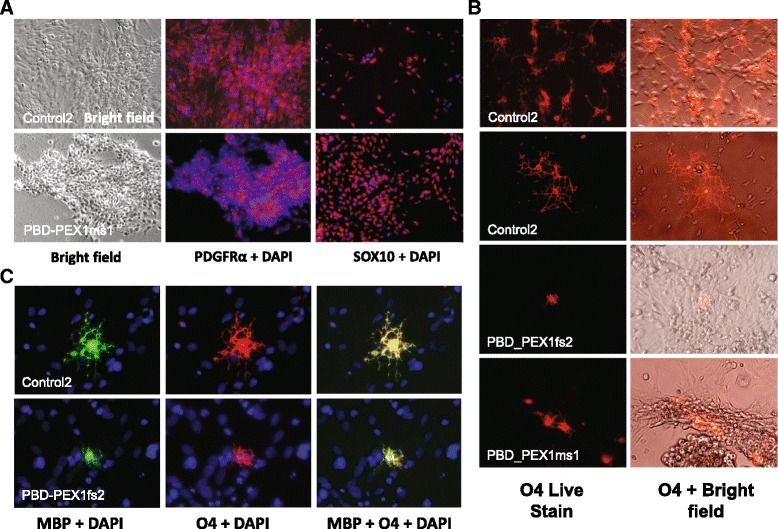
OPCs and mature OLs derived from PBD-ZSD patient and healthy control iPSCs. **a** Images of OPCs derived from the indicated patient (*top row*) and healthy control (*bottom row*) donor immunostained for the OPC markers, PDGFRα and SOX10. **b** Images of OLs derived from the indicated patient and healthy control donors immunostained for O4 without (*left*) and with (*right*) bright field staining. **c** Images of mature OLs derived from the indicated patient (*top row*) and healthy control (*bottom row*) immunostained for myelin basic protein (MBP) and O4 using the same magnification. *Arrows* indicate patient cell that is positive for MBP and O4. *Blue* color represents DAPI nuclear counterstaining in all cases

Given their relevance to the pathophysiology of PBD-ZSD [49, 50], we primarily focused on deriving OL cell lineages from iPSCs. We demonstrated that patient and control iPSCs could be differentiated into morphologically indistinguishable OP cells (OPCs) (Fig. 5a) and passaged a similar number of times. Two weeks after growth factor withdrawal, large numbers of healthy control OPCs differentiated into highly branched mature OLs expressing O4 and MBP (Fig. 5b, c). In contrast, patient-derived OPCs cultured under the same conditions produced a limited number of small poorly branched O4-positive cells which could not be maintained as a monolayer of attached cells (Fig. 5b). Instead, they detached shortly after growth factor withdrawal and tended to form neural spheres, while control-derived OLs could be maintained as a monolayer. Nevertheless, we did observe a small and poorly branched PBD-ZSD patient-derived OL showing O4- and MBP-positive staining (Fig. 5c).

### Relative sVCLFA and plasmalogen levels in iPSC and CNS cells from PBD-ZSD patients and controls

To begin to investigate the effects *PEX* gene mutations have on lipid catabolism in different cell types, we evaluated the levels of sVLCFAs in patient- and control-derived fibroblasts, iPSCs, and neural progenitor cells (NPCs). Consistent with prior reports [51], PBD-ZSD patient skin fibroblasts cultured in fibroblast growth media showed elevated sVLCFA levels relative to controls (Table 2, Additional file 3). The magnitudes of the increases were also consistent with prior knowledge of the biochemical defect and known or predicted residual *PEX* gene functions [52–55]. Since their *PEX* gene mutations are compatible with normal relative sVCLFA levels in fibroblasts cultured under standard conditions [53, 54], we removed PBD_PEX10 and PBD_PEX12 and their derivatives from further data analysis. Overall, the remaining group of PBD-ZSD fibroblasts had ≥13.0-fold increased sVLCFA levels relative to controls (*P* < 7×10^−3^ for C26:0LPC/C22:0LPC and %C26:0LPC, see Methods). Similar results were obtained for patient and control fibroblasts cultured in iPSC growth media (Table 2).

**Table 2:** Relative saturated very long chain fatty acid levels in cultured patient- and control-derived cells

	C26:0LPC/C22:0 LPC^a^				%C26:0LPC^a^			
Cell type	Mean control	Mean patient	FC^b^	*P* ^c^	Mean control	Mean patient	FC^b^	*P* ^c^
Fibroblast^d^	1.24	16.44	13.3	<3×10^−4^	0.82	10.33	12.6	<9×10^−6^
Fibroblast^e^	0.70	13.96	19.8	<7×10^−3^	0.23	3.36	14.3	0.01
iPSC^f^	6.61	1.31	−5.0	0.001	0.36	0.11	−3.4	0.07
NPC^g^	7.12	8.25	1.2	0.8	1.68	1.60	−1.1	0.9

^a^Geometric means of the indicated ratios are provided. %C26:0LPC relative to the sum of all LPCs and Lyso-PAFs measured (see Methods). ^b^Fold-change (FC): ratio of patient to control data. ^c^Based on two-tailed Student *t*-test of log-transformed data. ^d^Cultured in fibroblast growth media (three control, five patient samples); ^e^Cultured in iPSC growth media (three control, five patient samples). ^f^Induced pluripotent stem cells (iPSCs): four control, 15 test samples; ^g^Neural progenitor cells (NPCs): two control, seven patient samples

Relative sVLCFA levels were significantly reduced in patient compared to control iPSCs cultured in iPSC growth media (5.0-fold, *P* = 0.001); however, differences in %C26:0LPC did not reach statistical significance (Table 2, Additional file 3). We did not conduct experiments examining fibroblast growth media due to compatibility issues. In our analysis of iPSC-derived cells, we found no statistically significant differences in the relative sVLCFA levels of patient- and control-derived NPCs (Table 2, Additional file 3). There were marked intra-group fluctuations in relative sVLCFA levels among NPCs, even among those derived from the same donor. This could reflect possible cellular heterogeneity within the NPCs, which were obtained by dissection of neural rosettes.

We expanded our studies to investigate the effects *PEX* gene mutations have on lipid biosynthesis in different cell types by determining the relative levels of PE plasmalogens in all the samples described above. In agreement with prior reports [51], PBD-ZSD patient skin fibroblasts with biallelic null mutations in *PEX1* (PBD_fs1 and PBD_fs2) grown in fibroblast or iPSC growth media showed over a threefold average reduction in relative PE plasmalogen levels compared to controls (Additional file 3, Student *t*-test not performed due to limited sample sizes). The relative PE plasmalogen levels in the remaining patient skin fibroblasts grown in either growth media were consistent with prior knowledge [52–55]. Relative PE plasmalogen levels were also significantly reduced in patient compared to control iPSCs (3.8-fold, *P* = 0.01) (Additional file 3). Consistent with our sVLCFA studies, there were significant intra-group fluctuations in the relative PE plasmalogen levels of NPCs, even among those derived from the same donor.

### Derivation and characterization of hepatocyte-like cells from control and PBD-ZSD patient fibroblasts

**Fig. 6:**
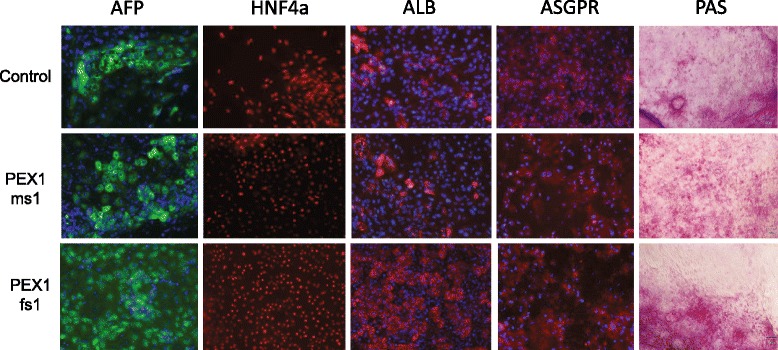
Characterization of iPSC-derived hepatocytes by immunostaining and Periodic acid-Schiff staining (PAS). Expression of AFP (*green*), HNF4a (*red*), ALB (*red*), and ASGPR (*red*) were detected by immunostaining. *Blue* represents DAPI staining. PAS staining was used to indicate glycogen storage

We induced two PBD-ZSD patient and one control iPSCs into definitive endoderm and hepatocyte-like cell cultures with similar efficiencies. After completion of the maturation protocol, hepatocyte-like cell cultures showed cells with positive immunostaining for albumin, AFP, HNF4a, and ASGPR (Fig. 6). We isolated ASGPR-positive cells by FACS analysis and conducted microarray-based global gene expression analysis, which indicated the robust expression of hepatocyte-like cell markers *AFP*, *ALB*, *APOA2*, *FOXA2*, *KRT8*, *KRT18*, *KRT19*, *SERPINA1* (also known as *AAT*), and *TTR* (Additional file 4). Nevertheless, we note that cytochrome P450 gene family members were poorly expressed in the hepatocyte-like cell cultures derived from both healthy individuals and patients, with the exception of *CYP1B1* which showed moderate to robust expression (Additional file 4). Although *CYP1B1* is not a specific hepatocyte marker, it has been noted that its induced expression suggests hepatocyte commitment and maturation [56]. Although immunostaining analysis indicated that the control- and patient-derived cell cultures were not homogenous, they all contained hepatocyte-like cells. The presence of hepatocyte-like cells was further supported by staining for glycogen storage using the PAS Assay kit (Fig. 6). Moreover, all hepatocyte-like cell cultures produced urea and albumin (Additional file 12).

### Patient-derived neural and hepatocyte-like cells show defects in peroxisome assembly

**Fig. 7:**
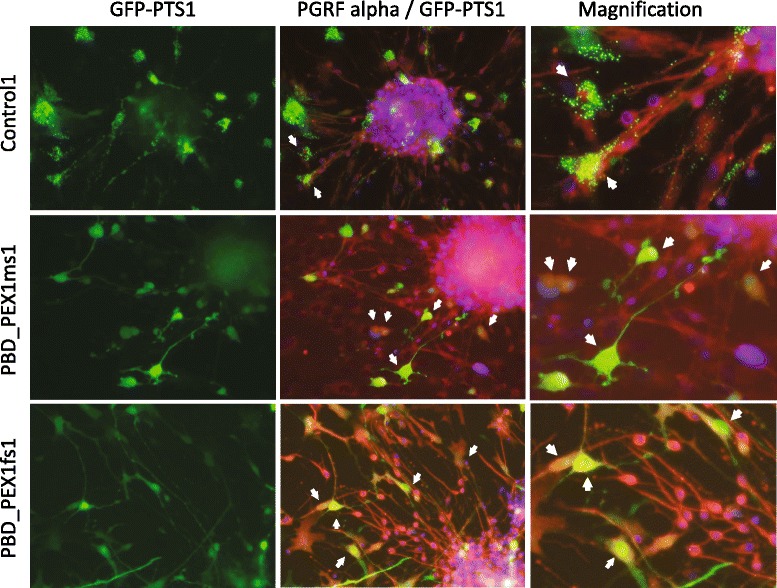
Peroxisome assembly in OPCs expressing the GFP-PTS1 reporter gene. Cells were immunostained with antibodies against PGFR-alpha (*red*) and nuclei were counterstained with DAPI (*blue*). As indicated by GFP-positive puncta of appropriate size relative to nuclei, GFP-PTS1 was imported into peroxisomes in control cells (*top row*) whereas in the marked PBD-ZSD patient cells (*bottom two rows*), GFP-PTS1 showed cytoplasmic localization, reflecting a peroxisome assembly defect. *Arrows* highlight cells co-expressing PGFR-alpha and GFP-PTS1

**Fig. 8:**
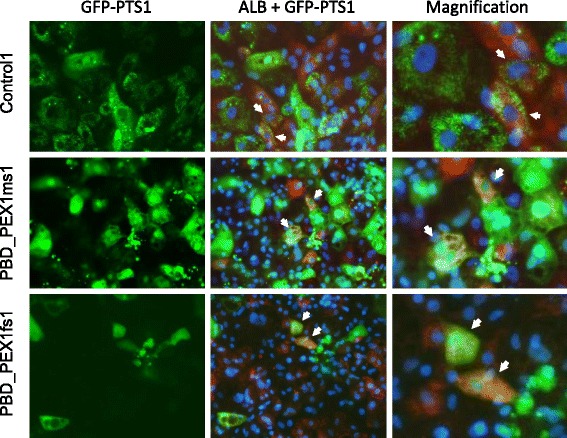
Peroxisome assembly in hepatocyte-like cells expressing the GFP-PTS1 reporter gene. Cells were immunostained with antibodies against ALB (*red*) and nuclei were counterstained with DAPI (*blue*). GFP-positive puncta of appropriate size relative to nuclei in control cells (*top strip*) indicate peroxisomal import of GFP-PTS1. In the marked patient cells (*bottom two rows*), GFP-PTS1 had cytoplasmic localization, indicating a peroxisome assembly defect. *Arrows* highlight cells co-expressing ALB and GFP-PTS1

Control- and patient-derived OPCs and hepatocyte-like cells were transduced with vectors expressing the GFP-PTS1 reporter protein that is imported into the peroxisome matrix in normal human cells, but remains cytosolic in PBD-ZSD patient cells with peroxisome assembly defect [18]. In all cases for OPCs and hepatocyte-like cells, the control cells showed abundant GFP-positive puncta with the appropriate size (relative to nuclei) and distribution consistent with robust peroxisome assembly (Figs. 7 and 8). In contrast, patient-derived cells all showed cytoplasmic GFP localization that reflects the peroxisome assembly defect in the donors (Figs. 7 and 8).

## Discussion

Although it is known that impaired peroxisome assembly is causally responsible for PBD-ZSD [57–59], the mechanisms underlying the cell type-specificity of disease are not fully understood. While most organ systems are affected, impaired CNS [12, 60, 61] and hepatic cell functions [62–64] play important roles in disease pathogenesis and progression. Here, we demonstrate that PBD-ZSD patient-derived skin fibroblasts with mutations in different *PEX* genes can be reprogrammed with similar efficiencies into iPSCs that could be maintained in culture for prolonged times. These patient-specific resources could provide a gateway to new models to investigate the cell-type specificity of peroxisome activities and their roles in the pathophysiology of PBD-ZSD.

To begin to explore their potential applications, we demonstrated that control- and patient-derived iPSCs would produce CNS cell types relevant to the etiology of PBD-ZSD. Despite considerable intra-group variation consistent with prior reports involving iPSCs derived from healthy donors [65], there was a statistically significant reduction in neural differentiation potency in patient- relative to control-derived EBs (Additional file 10A). Additional studies are required to confirm these observations given the large number of variables under consideration including the limited numbers of cell lines investigated and iPSC passage numbers. In contrast, the timing of neural rosette formation upon induction was similar between control- and patient-derived EBs (Additional file 10B).

Patient-derived iPSCs were capable of differentiating into a variety of neural cell types, including electrically active neurons (Fig. 4e, f). More pertinent to the neuropathogenesis of PBD-ZSD [12], we could generate OPCs from both patient and control iPSCs; however, it was considerably more difficult to generate MBP- and O4-expressing branched mature OLs from PBD-ZSD patient relative to healthy control cells. The poor branching of PBD patient-derived O4-positive cells and their inability to be maintained as a monolayer during differentiation should be explored in larger-scale studies to determine definitively if peroxisome dysfunction is casually responsible for these observations.

In parallel, we demonstrated that control- and patient-derived iPSCs are capable of producing hepatocyte-like cells, which displayed the appropriate protein and gene expression markers, cell morphology, and ability to store glycogen (Fig. 6). Moreover, control- and patient-derived hepatocyte-like cell cultures produced urea and albumin (Additional file 12). Although our studies were limited, control- and patient-derived iPSCs showed similar abilities to differentiate into hepatocyte-like cells.

To evaluate peroxisome assembly, we transduced control- and patient-derived neural precursor and hepatocyte-like cells with vectors designed to express a GFP-PTS1 reporter protein (i.e., GFP with a C-terminal peroxisome targeting signal). In keeping with expectations, control-derived cells showed punctate GFP fluorescence indicative of robust peroxisome assembly, while patient-derived cells showed diffuse cytoplasmic localization of the GFP-PTS1 reporter protein indicative of impaired peroxisome assembly (Figs. 7 and 8). Future studies evaluating the localization and abundance of other peroxisomal proteins in control- and patient-derived iPSCs, through immunostaining [17] and proteomics technologies [4, 5], could be of value towards elucidating the cell type specificity of peroxisome functions.

In agreement with prior work [66], patient-derived skin fibroblast cultures had elevated relative sVLCFA and %C26:LPC levels compared to those from healthy controls (Table 2). The only exceptions involved cells from donors PBD_PEX10 and PBD_PEX12, which have *PEX* gene mutations that are associated with relative sVLCFA levels in the normal range in skin fibroblasts [53, 54] and thus were not considered in iPSC and NPC lipid data analyses. In contrast, patient-derived iPSCs had significantly lower relative sVLCFA levels than controls with no statistically significant differences in %C26:0LPC levels (Table 2). Moreover, there were no statistically significant differences in the relative sVLCFA or %C26:0LPC levels in the patient- and control-derived NPCs analyzed, but possible cellular heterogeneity within and among NPCs should be taken into consideration when interpreting these results.

It is relevant to compare these biochemical analyses with those of iPSCs derived from the skin fibroblasts from individuals with X-linked adrenoleukodystrophy (X-ALD) [20, 67], a complex neurological disorder caused by mutations in the *ABCD1* gene that encodes a peroxisome membrane protein [68–70]. Males with *ABCD1* null mutations have elevated sVLCFA levels in their blood and urine and reduced sVLCFA catabolic activity in their cultured skin fibroblasts, but otherwise normal peroxisome assembly and metabolic activities. Similar to our observations involving PBD-ZSD patient-derived cells, X-ALD patient-derived iPSCs had low relative sVLCFA levels [20, 67]. Intriguingly, X-ALD iPSCs can be differentiated into OLs with elevated relative sVLCFA levels [67]. Our current studies provide additional evidence for the existence of cell type-specific lipid abnormalities that result from peroxisome dysfunction. Further studies are needed to determine if these observations have relevance to the developmental abnormalities and/or degenerative conditions found in individuals with PBD-ZSD and other peroxisomal disorders, such as X-ALD.

As previously discussed [20], cellular sVLCFA levels are influenced by their rates of biosynthesis and catabolism, rates of cell proliferation, and uptake from culture medium [67]. We examined the expression levels of *ELOVL* gene family members that encode fatty acid elongating enzymes critical for VLCFA biosynthesis. In agreement with our prior report [20], the pivotal *ELOVL1* family member responsible for the elongation of C22:0 to C24:0 and C26:0 fatty acids [71, 72] had significantly higher expression in fibroblasts relative to iPSCs, regardless of donor health status. We observed the differential expression of one ELOVL family member (*ELOVL5*), which had a modest 1.4-fold higher expression in patient relative to control iPSCs (FDR =0.008). Given that it is involved in the elongation of C18:3, n-6 to C20:3, n-6 and C18:4, n-3 to C20:4, n-3 fatty acids in mouse liver [73], the modest elevation in ELOVL5 transcript levels is unlikely to explain the reduced relative sVLCFA levels in patient iPSCs. Other genes directly involved in peroxisomal VLCFA catabolism were not differentially expressed in patient relative to control fibroblasts or iPSCs. Regarding other hypotheses, it is technically challenging to directly address cell proliferation rates and media uptake given the specialized conditions required for the growth and maintenance of iPSCs and their derivatives. As mentioned in our prior studies [20], the lower sVLCFA levels in iPSC relative to fibroblast growth media could influence lipid profiles. The MEF feeders in the iPSC media could as well; however, the comparisons of control and patient cells were made under the same growth conditions. Experiments using a variety of controlled growth media could be useful to further investigate the cell type specificity of aberrant lipid levels in patient cells.

To further address the cell type specificity of lipid metabolic defects, we determined relative PE plasmalogen levels in fibroblasts, iPSCs, and NPCs from patients and controls. We focused on cells with biallelic null mutations in a given *PEX* gene since this should result in marked plasmalogen deficiencies due to the crucial roles peroxisome plays in plasmalogen biosynthesis [6–8]. In agreement with these expectations, all such patient-derived cells (fibroblasts, iPSCs, and NPCs) showed low relative PE plasmalogen levels (Additional file 3). Patient fibroblasts, iPSCs, and NPCs with partially functional hypomorphic *PEX* gene alleles provided relative PE plasmalogen levels generally consistent with prior reports of the plasmalogen biosynthetic activity in the starting fibroblast cultures [52–55]. Overall, the abnormalities in relative sVLCFA levels observed in patient-derived cells showed more striking cell type specificities than the corresponding abnormalities in relative PE plasmalogen levels. This suggests that studies into the cell type specificity of other peroxisomal metabolic pathways, such as bile acid and amino acid metabolism, may be warranted in patient and control cells in the future.

Similar to our experiences with iPSCs from X-ALD patients [20], PBD-ZSD patient-derived iPSCs, but not fibroblasts, showed gene expression signatures consistent with proposed mechanisms of pathogenesis. Most striking were groups of DEGs enriched for organelle localization, especially those of mitochondrial function which tended to be upregulated in patient iPSCs relative to controls. Although mtDNA levels were consistent among all control- and patient-derived fibroblasts (Fig. 3), they were variable among all iPSCs, even different colonies from the same donor. This is in general agreement with prior work showing variation in mtDNA levels, and even mtDNA mutation status, among iPSCs [74–76], with changes in mtDNA content even reported according to the passage number of a given iPSC colony [77]. Nevertheless, there were no reproducible differences in mtDNA levels among patient- and control-derived iPSCs. Future studies may focus on mtDNA mutation status in patient-derived cells and their changes according to passage number.

Historically, there have been numerous reports of mitochondrial abnormalities in the peroxisome-deficient mammalian cells [78–82]. More recently, the extent of molecular cross-talk between mitochondria and peroxisomes has been increasingly appreciated. This has been spurred on by the discovery of mitochondrial derived vesicles (MDVs) that transport cargo to a subpopulation of peroxisomes and lysosomes [83–86]. Although likely to be complex, to date the only cargo known of MDVs transported from mitochondria to peroxisomes is the mitochondrial outer membrane protein MAPL [83–86]. We also note a body of literature linking mitochondrial dysfunction with X-ALD [87–89].

Furthermore, DEGs showed enrichment for gene related to ER and Golgi function. There is a clear link between peroxisome and ER biology given the fact that peroxisome arises from the ER through a de novo pathway involving membrane budding [1]. Likewise, ER stress has been observed in peroxisome-deficient cells [78, 90–92]. The Golgi enrichment could reflect alterations in cell trafficking and cellular communication that would require further functional characterization. For example, it is known that the ER, the Golgi, and the peroxisome are all involved in the generation of the lipid portion of GPI-anchored proteins, which are associated with lipid rafts [46]. Indirect evidence based on the clinical phenotypes of individuals with mutations in genes involved in GPI-anchor protein biosynthesis indicates that GPI-anchor protein abnormalities can result in intellectual disabilities [93].

In terms of future applications and directions, there are numerous opportunities to improve patient-derived models of PBD-ZSD and other diseases. For example, targeted genetic modifications present an emerging strategy for modeling age-related disease phenotypes in cell culture [94]. iPSCs could also provide the basis for co-culture models, especially those involving neurons and OLs, or three-dimensional organoid models to investigate noncell autonomous processes relevant to disease pathogenesis and progression [95]. Nevertheless, we respect that it remains a challenge to generate in vitro model systems for PBD-ZSD and other complex disorders that involve multiple organ systems and possible gene–environment interactions.

## Conclusions

The iPSCs reported herein complement PBD-ZSD patient-derived fibroblast culture models and a diverse group of animal models that have provided valuable insights into the pathomechanisms of disease [96–100]. Patient-derived iPSC models provide the unique advantages of representing *PEX* gene mutations and possible modifier genes in cell types most relevant to clinical phenotypes. Our patient cohort includes a diverse spectrum of *PEX* gene mutations with varying activity, including those with two null *PEX* gene alleles and two hypomorphic *PEX* gene alleles that confer partial function. This presents opportunities to evaluate mutation-specific therapies (including nonsense suppressor drugs [101] and molecular chaperones [18, 102] for individuals with the common *PEX1* p.G843D missense mutation) in relevant cell populations. The multiple iPSC colonies we generated for each PBD-ZSD patient will help to minimize confounding effects that extraneous genomic sequence changes (conferred due to reprogramming) have on the model system. We also note that patient-derived cell models are not subject to species–specific differences in peroxisome biology [103–105] that could confound the evaluation of some targeted therapies. This is perhaps best illustrated by differences in peroxisome proliferation observed in murine and human cells in response to PPAR-alpha agonists [3].

Finally, the demonstration by GFP-PTS1 reporter assays that patient-derived neural and hepatic cell lineages have impaired peroxisome assembly provides important proof-of-concept that they could be used for quantitative, cell-based, high-content screening (HCS) for compounds that improve peroxisome assembly. Once optimized for cell number and purity, iPSC-derived cells could be used to build upon the success of a prior HCS study that uncovered small molecules which improve peroxisome assembly in PBD-ZSD patient fibroblasts [18]. Likewise, we note the emerging role of iPSCs in toxicology assays for potential liabilities of therapeutic agents [106] and the possibility of uncovering environmental exposures that could more severely impact patients with PBD-ZSD and individuals with other diseases associated with peroxisomal dysfunction. In a broader context, the results of HCS using PBD-ZSD patient-derived neural and hepatic cells could help address fundamental questions regarding the potential benefits of evaluating multiple patient cell types in drug discovery efforts for a variety of disorders.

## Additional files

Additional file 1:
**Characterization of candidate iPSCs.** A complete listing of the protein, genetic, epigenetic, and cell differentiation assays performed on the iPSCs generated in this study is provided. (XLSX 11 kb) Additional file 2:
**Log-transformed gene expression scores from PBD-ZSD patient and healthy donor control fibroblasts and iPSCs.** A complete list of log-transformed gene expression scores from PBD-ZSD patient and healthy donor control fibroblasts and iPSCs is provided. (XLSX 11932 kb) Additional file 3:
**Lipid composition of cultured PBD-ZSD patient and control-derived cells.** A complete list of all biochemical measurements pertinent to the lipid composition of the cells discussed herein is provided. (XLSX 61 kb) Additional file 4:
**Gene expression analysis of ASGPR-positive hepatocyte-like cell cultures.** A complete list of log-transformed gene expression scores from PBD-ZSD patient and healthy donor control ASGPR-positive hepatocyte-like cell cultures is provided. (XLSX 3605 kb) Additional file 5:
**Immunostaining and alkaline phosphatase staining of iPSCs.** Immunostaining data of pluripotency markers and alkaline phosphatase staining data for representative iPSCs described in this study is provided. (PDF 6195 kb) Additional file 6:
**Copy number variation in PBD-ZSD patient and healthy control-derived iPSCs.** A full list of copy number changes (CNCs) detected for iPSCs in this study is provided. (XLSX 17 kb) Additional file 7:
**Hierarchical clustering analysis of data from genes related to pluripotency from PBD-ZSD patient and control fibroblasts and iPSCs.** This analysis was based on gene expression data from 30 pluripotency genes reported in reference [44]. (PPTX 934 kb) Additional file 8:
**Differentially expressed genes between PBD-ZSD patient and control healthy donor-derived iPSCs.** A complete list of all gene expression scores and differentially expressed genes (DEGs) between PBD-ZSD patient and healthy donor control fibroblasts is provided. (XLSX 77 kb) Additional file 9:
**Pathway analysis of DEGS between PBD-ZSD patient and healthy donor-derived iPSCs.** Gene Ontology (GO), KEGG, and IPA Pathway analyses of patient and healthy donor control fibroblasts are provided. (XLSX 546 kb) Additional file 10:
**Differentiation potential of iPSCs to neural rosettes.** The differentiation potential of PBD-ZSD and healthy control-derived iPSCs to neural rosettes was determined based on the percentage of attached EBs that formed neural rosettes in culture. In addition, we analyzed the number of days required for EB differentiation into neural rosettes. (PPTX 370 kb) Additional file 11:
**Differentiation potential of iPSCs to neural rosettes.** Representative images of healthy control and PBD-ZSD patient-derived cells in various stages of neural differentiation. (PDF 11822 kb) Additional file 12:
**Activities of hepatocyte-like cell cultures.** Data relevant to the levels of albumin and urea produced in PBD-ZSD patient and healthy control hepatocyte-like cell cultures is provided. (XLSX 9 kb)

## Abbreviations

CIRC: Coriell Institute Cell Repositories
CNC: Copy number change
CNS: Central nervous system
CNV: Copy number variation
DEG: Differentially expressed gene
EB: Embryoid body
EGF: Endothelial growth factor
ER: Endoplasmic reticulum
FBS: Fetal bovine serum
FDR: False discovery rate
FGF: Fibroblast growth factor
gDNA: Genomic DNA
GEO: Gene Expression Omnibus
GFP: Green fluorescent protein
GO: GeneOntology
GPI: Glycosylphosphatidylinositol
GRM: Glial restrictive medium
HCS: High-content screening
iMEF: Inactivated mouse embryonic fibroblast
IPA: Ingenuity Pathways Analysis
iPSC: Induced pluripotent stem cell
KEGG: Kyoto Encyclopedia of Genes and Genomes
IRD: Infantile Refsum disease
LC-MS/MS: Liquid chromatography–tandem mass spectrometry
LPC: Lysophosphorylcholine
MDV: Mitochondrial derived vesicle
NALD: Neonatal adrenoleukodystrophy
NCBI: National Center for Biotechnology Information
NDM: Neural differentiation medium
NE: Neural epithelia
NIM: Neural induction medium
NPC: Neural progenitor cell
OL: Oligodendrocyte
OP: Oligodendrocyte progenitor
OPC: Oligodendrocyte progenitor cell
PAF: Platelet activating factor
PAS: Periodic Acid-Schiff
PBD: Peroxisome biogenesis disorder
PBD-ZSD: Zellweger spectrum disorder
PE: Phosphatidylethanolamine
RA: Retinoic acid
SHH: Sonic hedgehog
SNP: Single nucleotide polymorphism
sVLCFA: Saturated very long chain fatty acid
X-ALD: X-linked adrenoleukodystrophy
ZS: Zellweger syndrome
