# Supplementary material for: Induced pluripotent stem cell models of Zellweger spectrum disorder show impaired peroxisome assembly and cell type-specific lipid abnormalities
Source: Stem Cell Res Ther. 2015 Aug 29;6:158. doi: 10.1186/s13287-015-0149-3 (PMC4553005; doi:10.1186/s13287-015-0149-3)
Supplement: Additional file 5: — Immunostaining and alkaline phosphatase staining of iPSCs. Immunostaining data of pluripotency markers and alkaline phosphatase staining data for representative iPSCs described in this study is provided. (PDF 6195 kb) [file 13287_2015_149_MOESM5_ESM.pdf]

Control1 iPS1

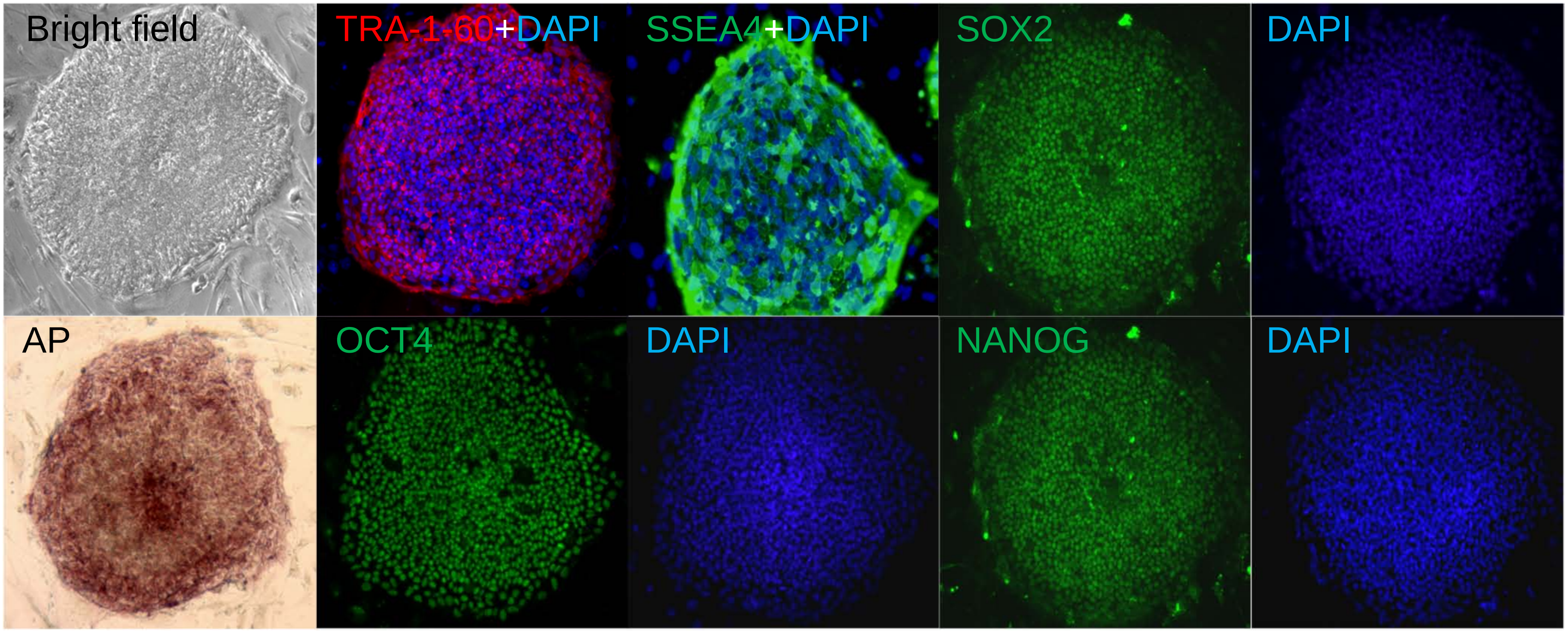

Donor fibroblast ID# AG05838

Control1 iPS3

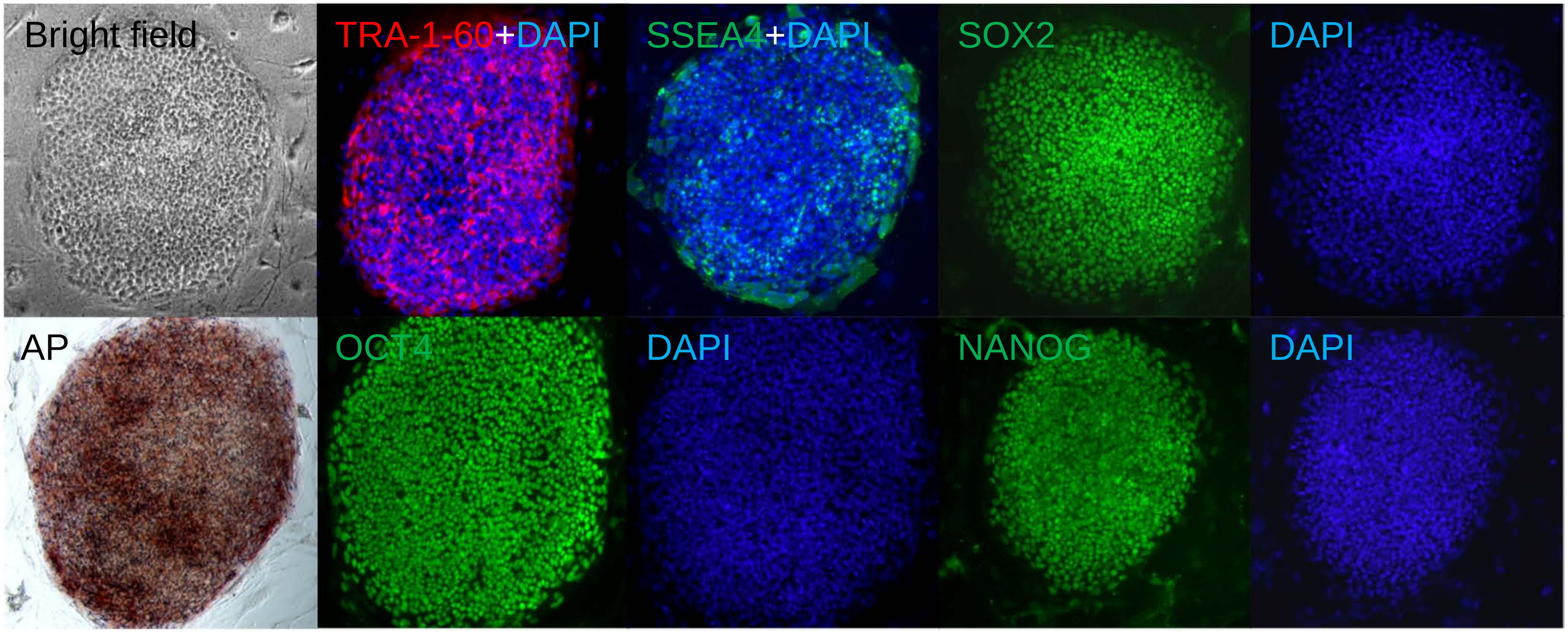

Donor fibroblast ID# AG05838

Control2 iPS1

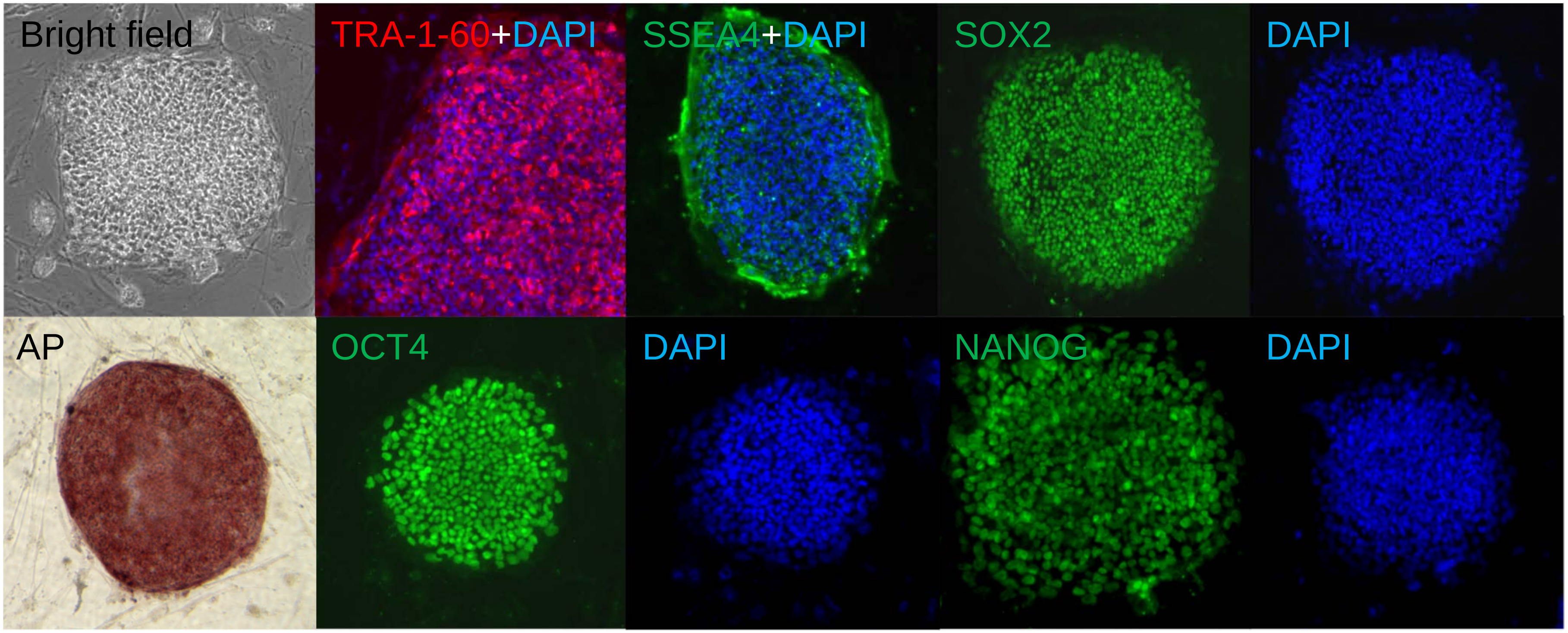

Donor fibroblast ID AG09599

Control2 iPS3

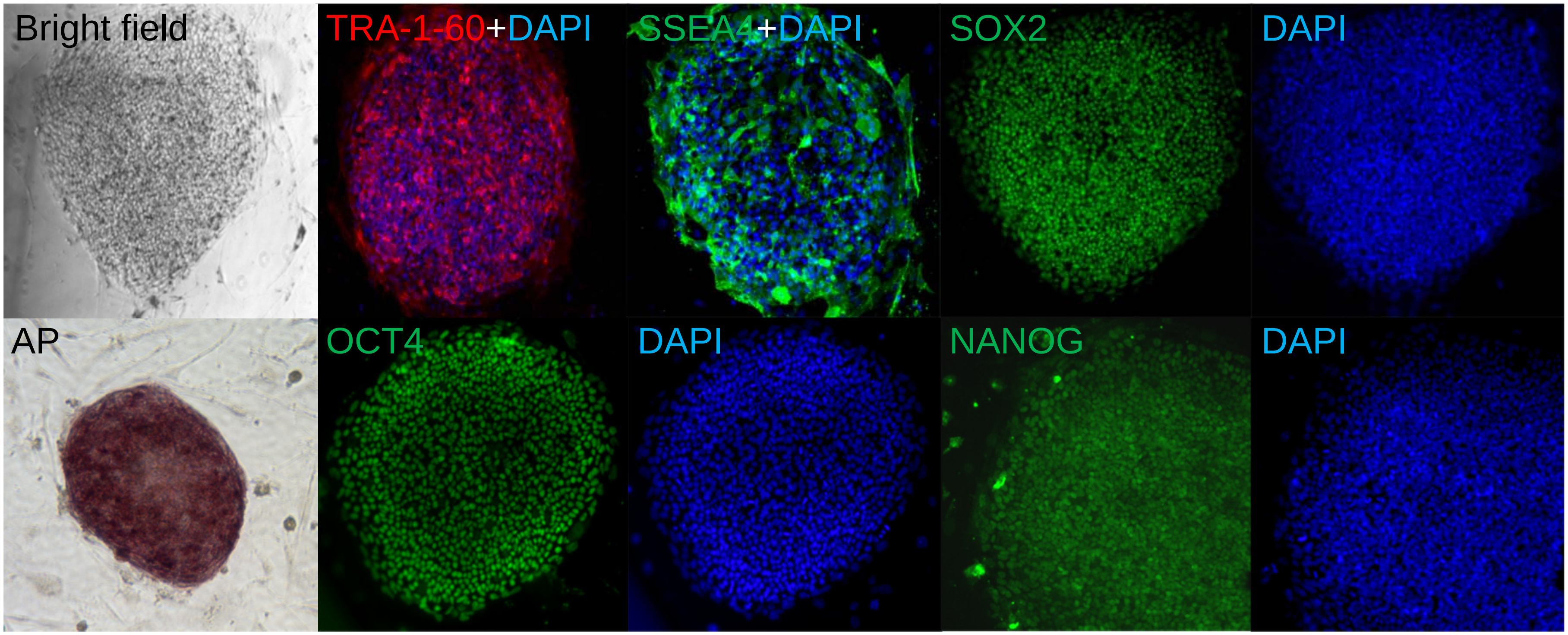

Donor fibroblast ID# AG09599

Control2 iPS4

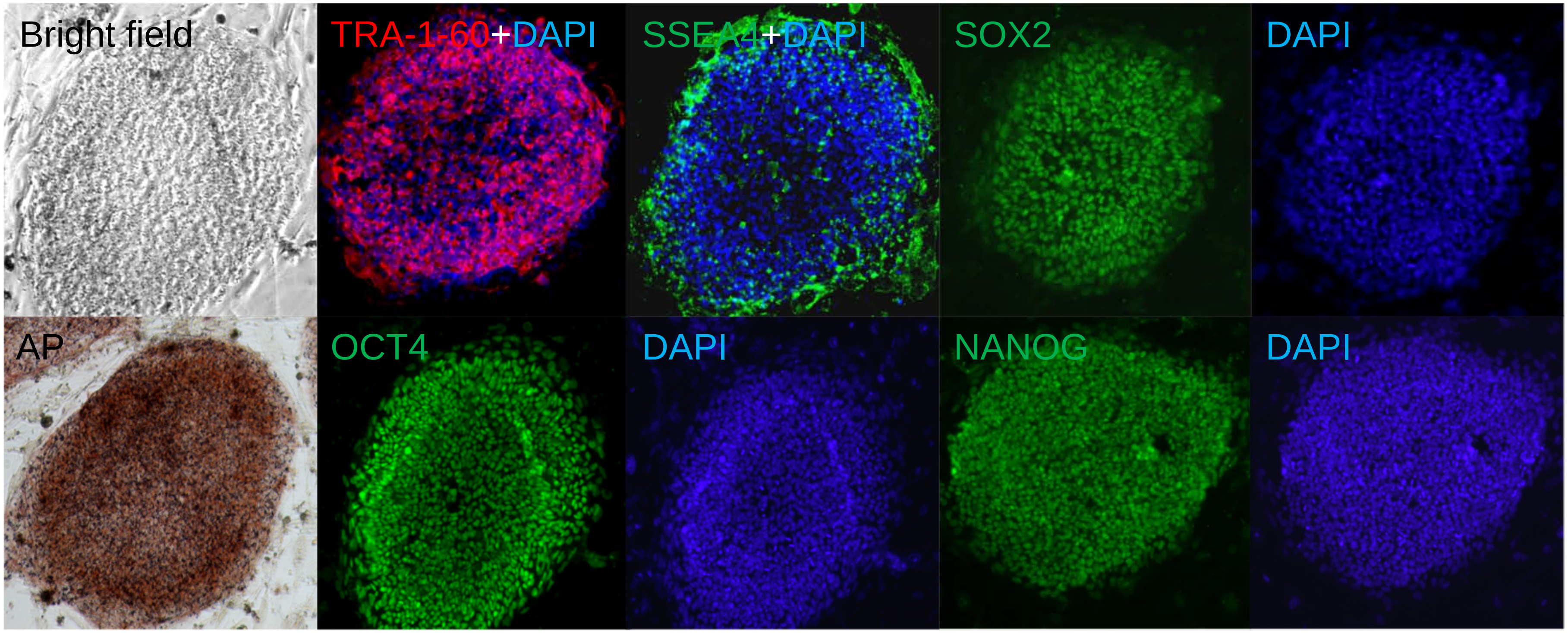

Donor fibroblast ID AG09599

Control3 iPS1

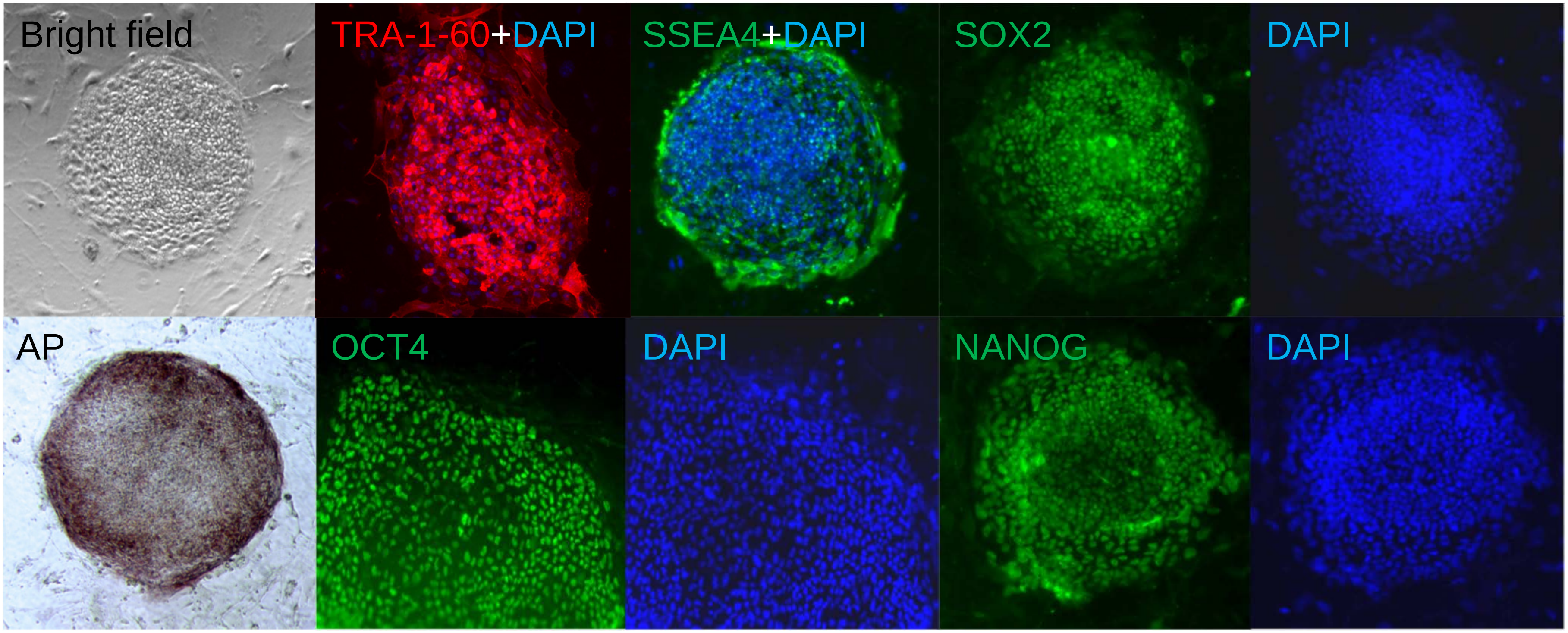

Donor fibroblast ID# AG13153

PBD_PEX1fs2 iPS3

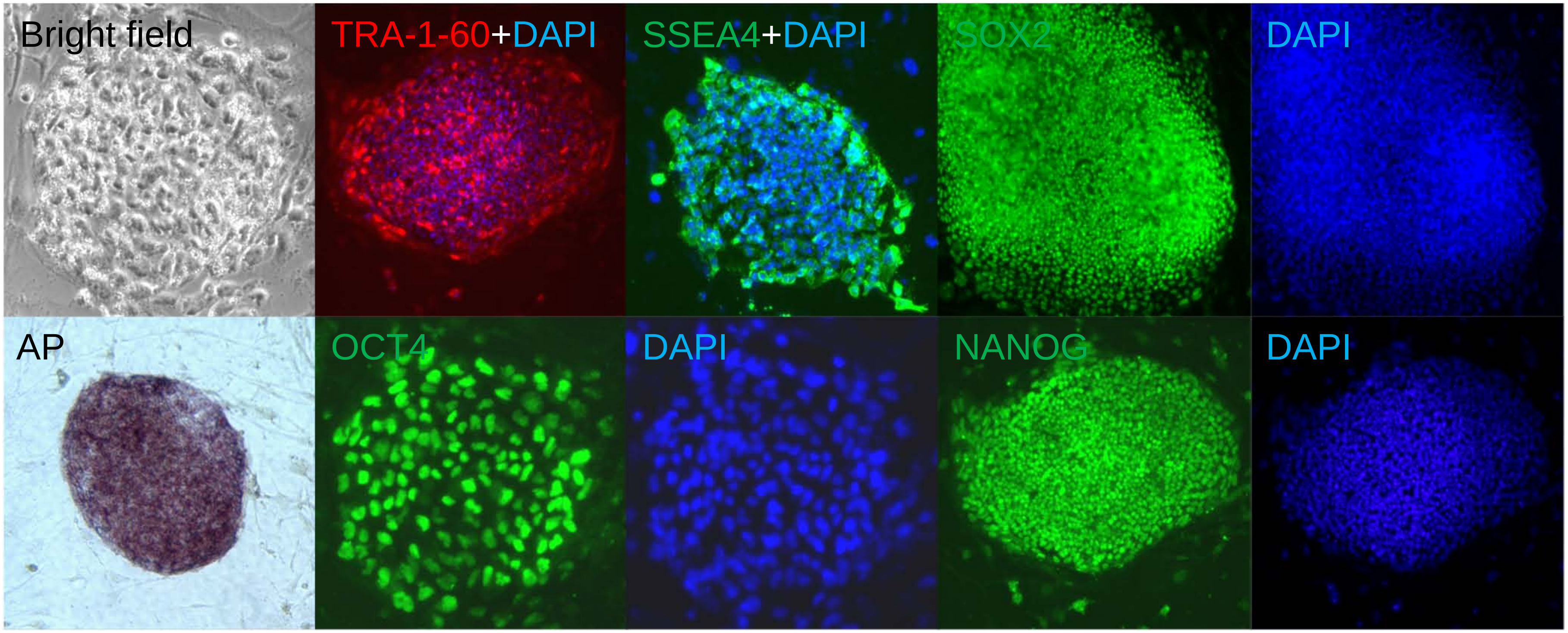

Donor Fibroblast ID# PBD702

PBD_PEX1fs1 iPS1

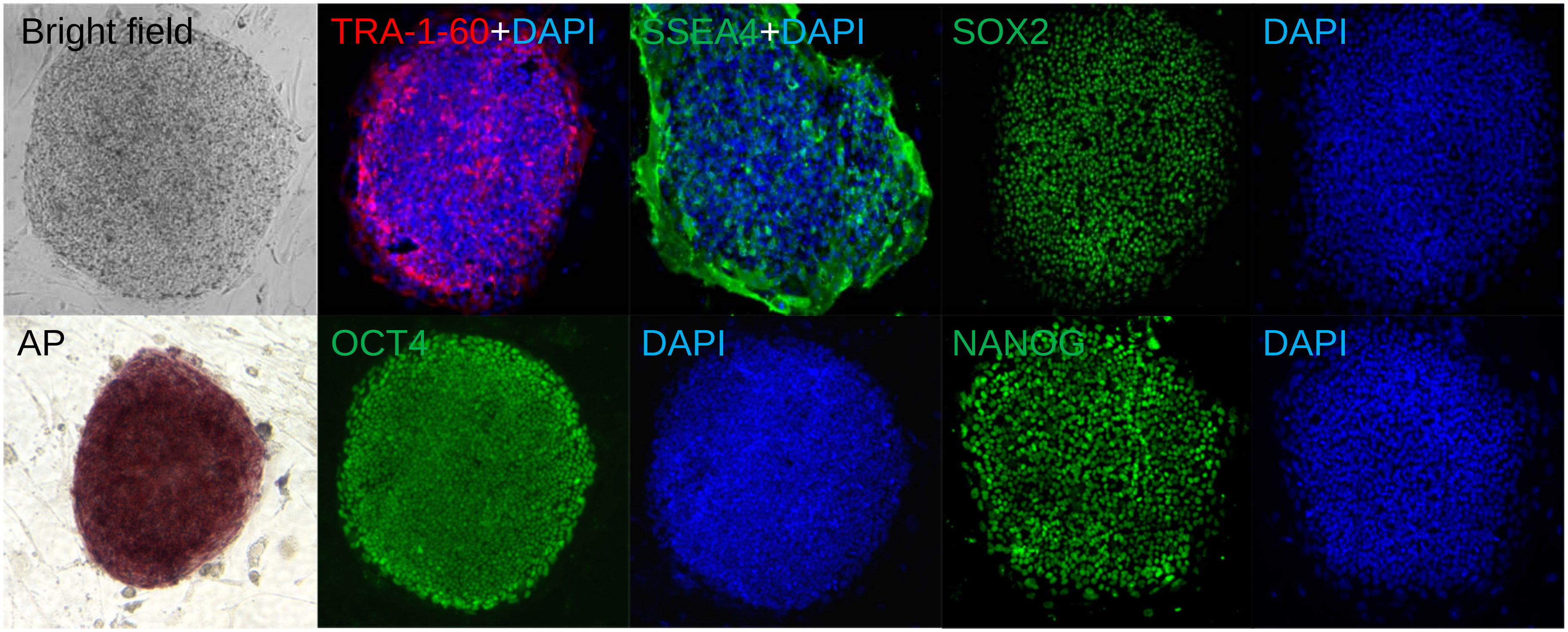

Donor Fibroblast ID# PBD721

PBD_PEX1fs1 iPS2

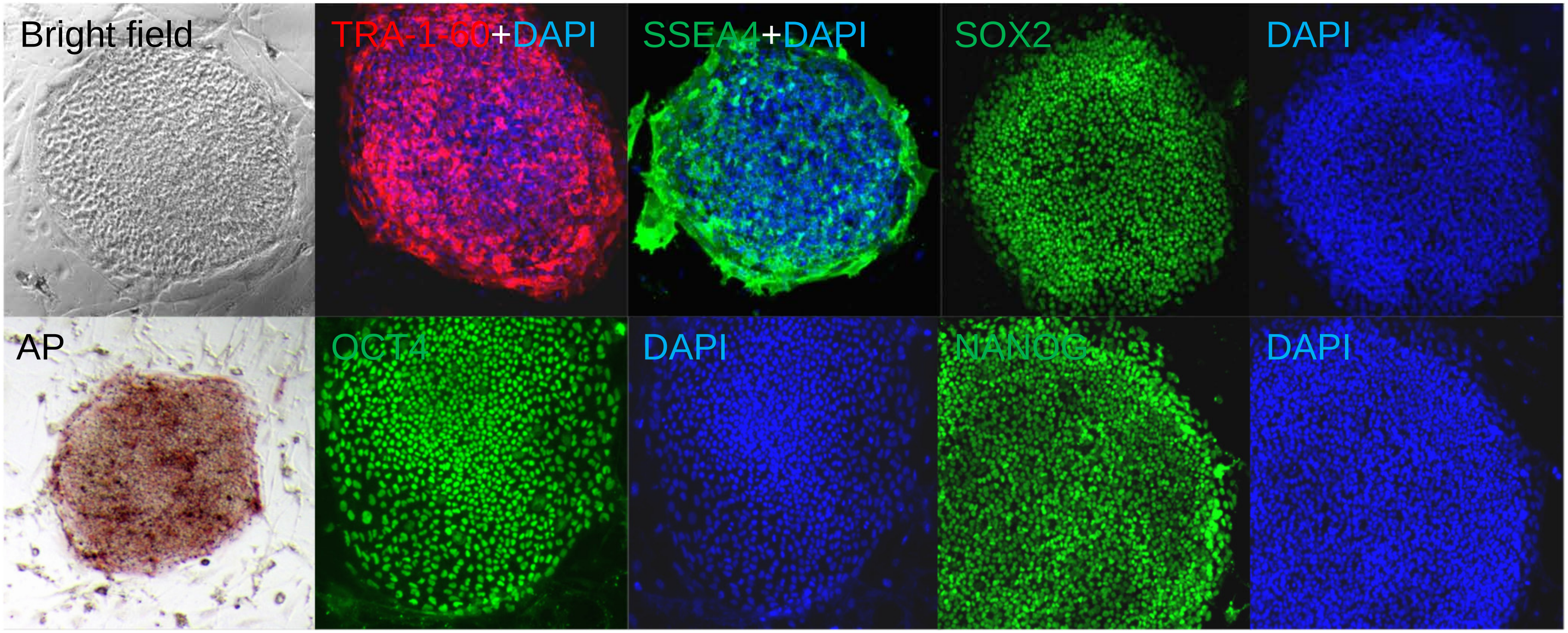

Donor Fibroblast ID# PBD721

PBD_PEX1ms1 iPS1

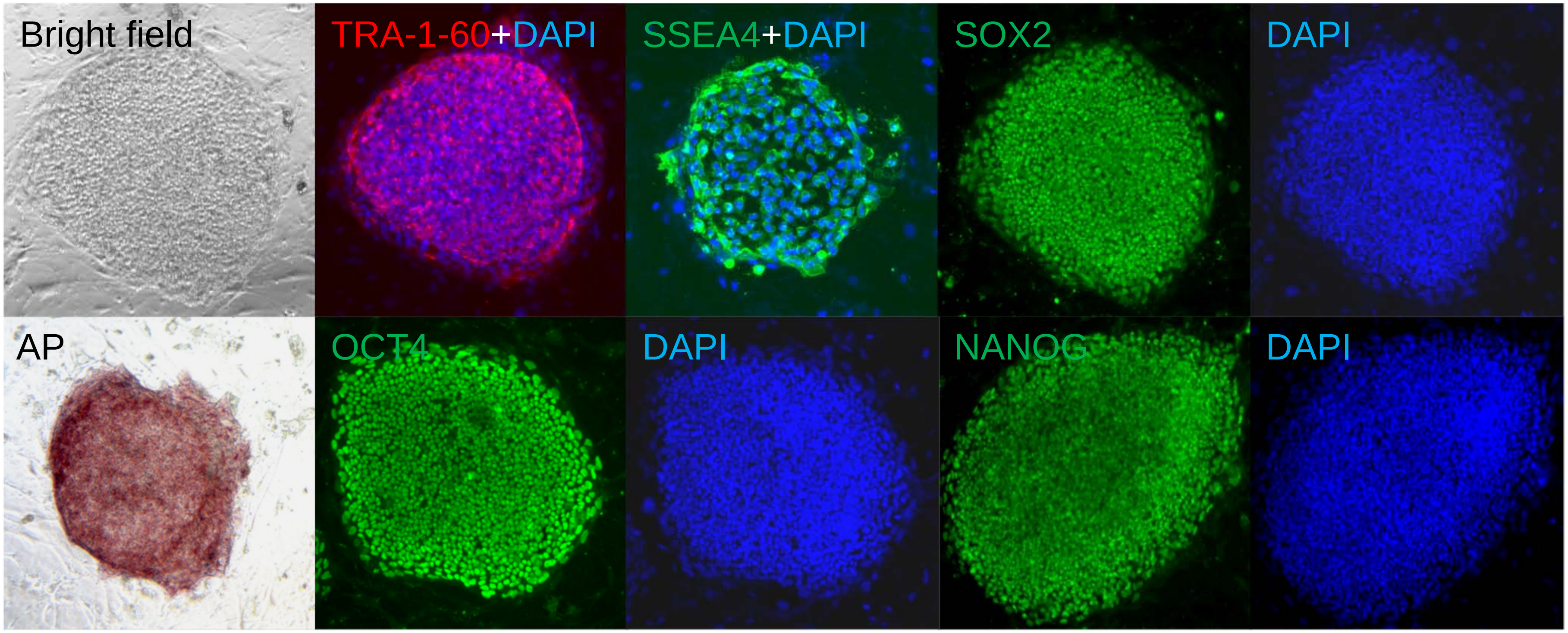

Donor Fibroblast ID# PBD615

PBD_PEX1ms1 iPS2

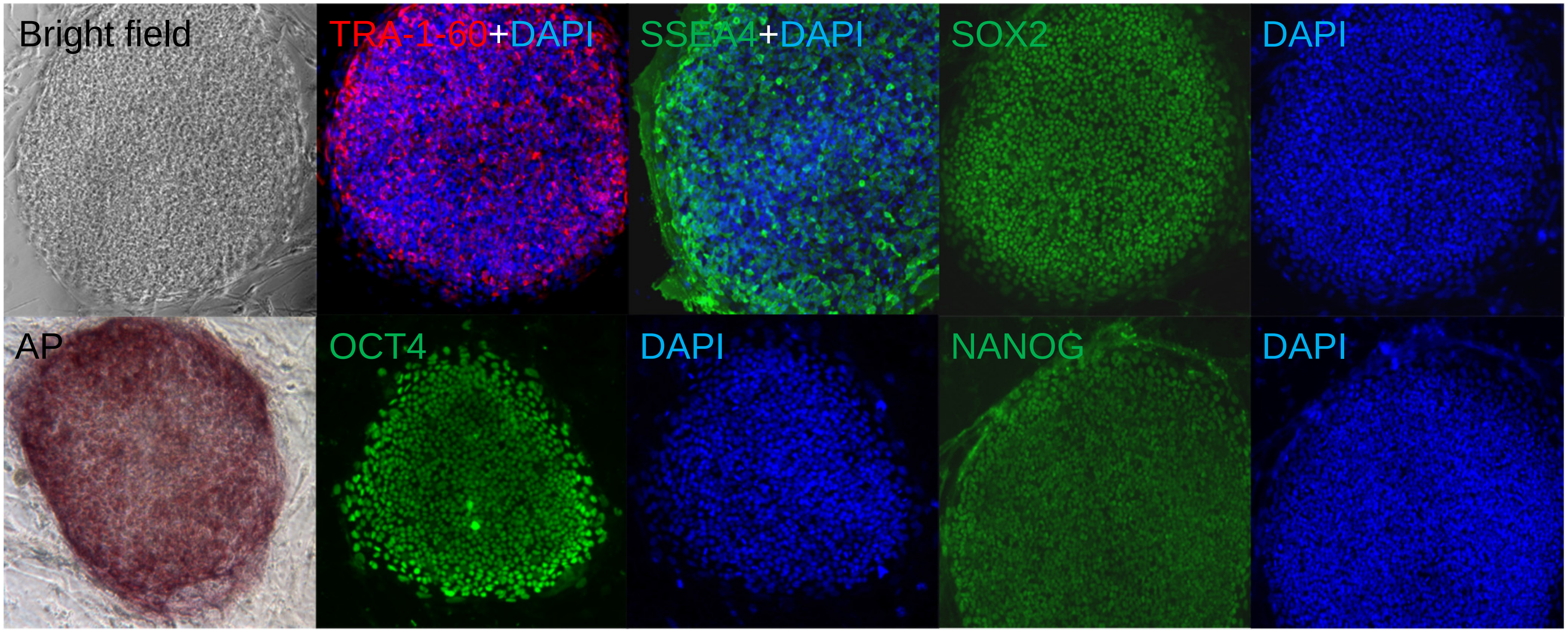

Donor Fibroblast ID# PBD615

PBD_PEX1ms1 iPS4

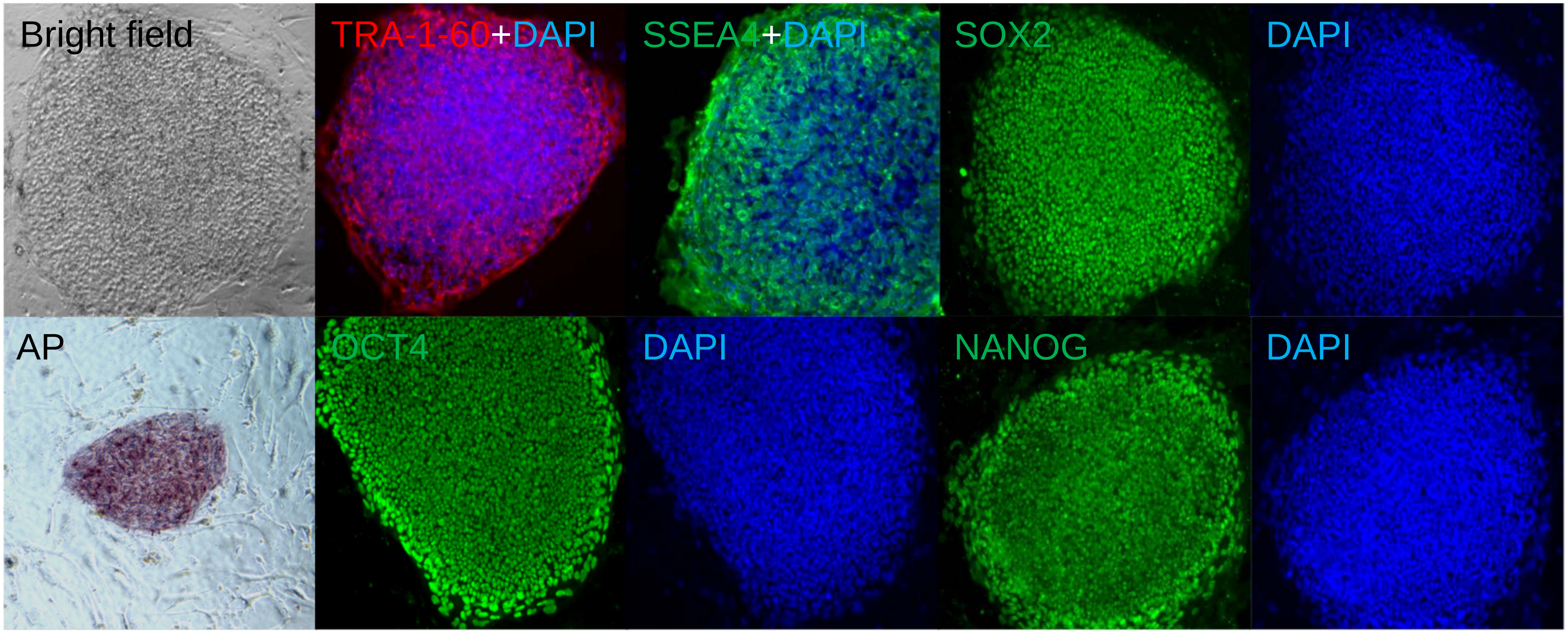

Donor Fibroblast ID# PBD615

PBD_PEX1ms1 iPS5

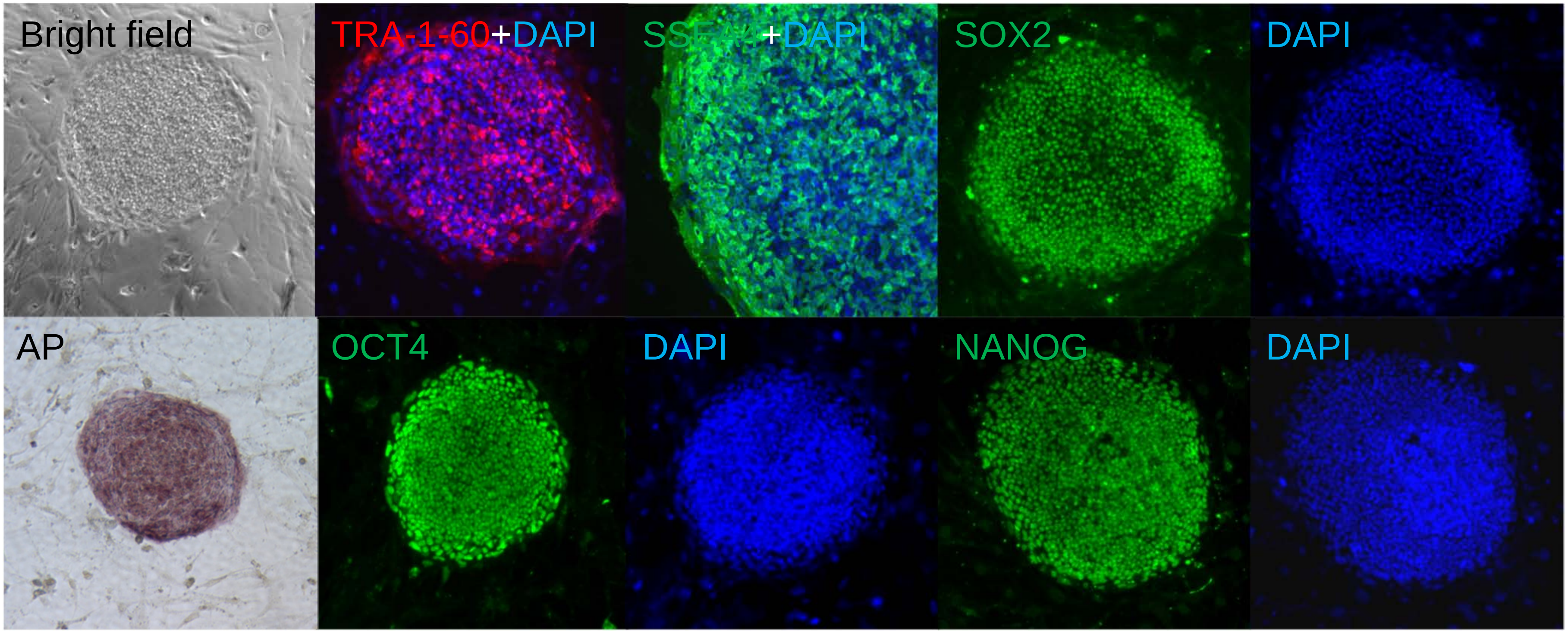

Donor Fibroblast ID# PBD615

PBD_PEX1ms2 iPS1

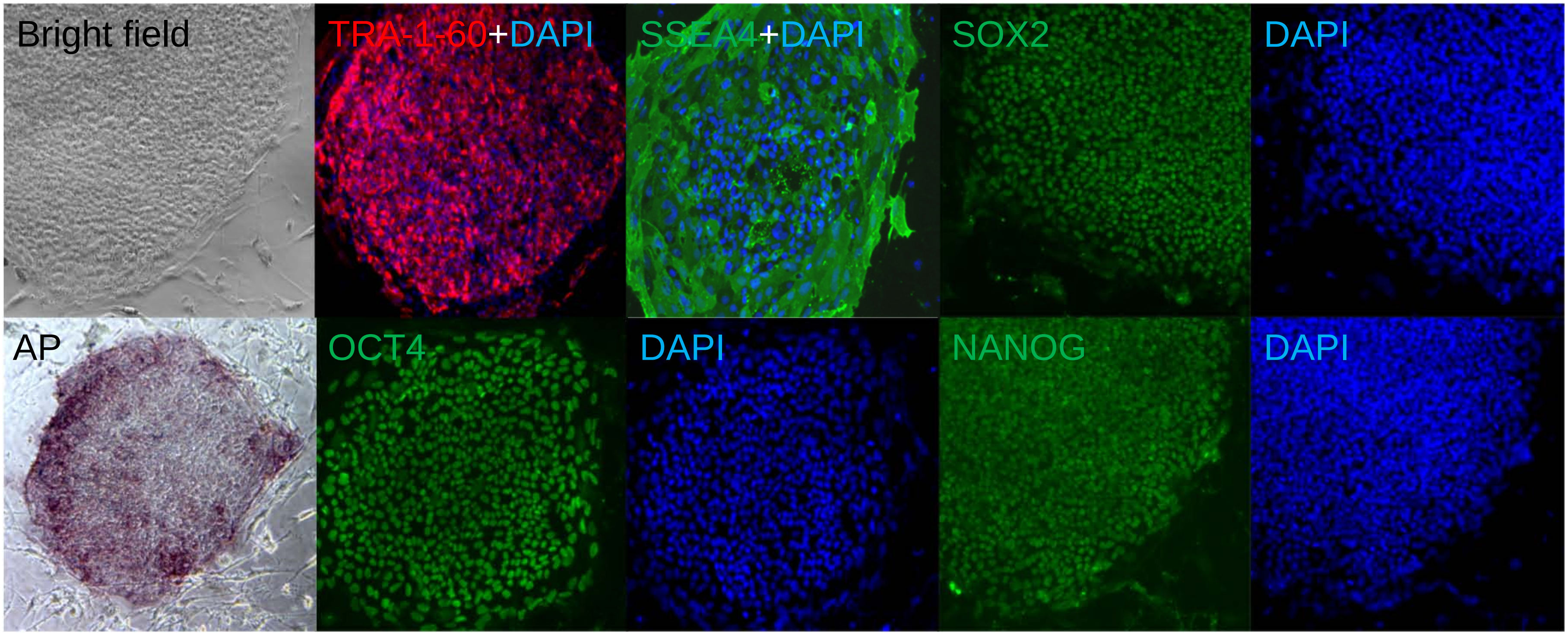

Donor Fibroblast ID# PBD643

PBD_PEX1ms2 iPS2

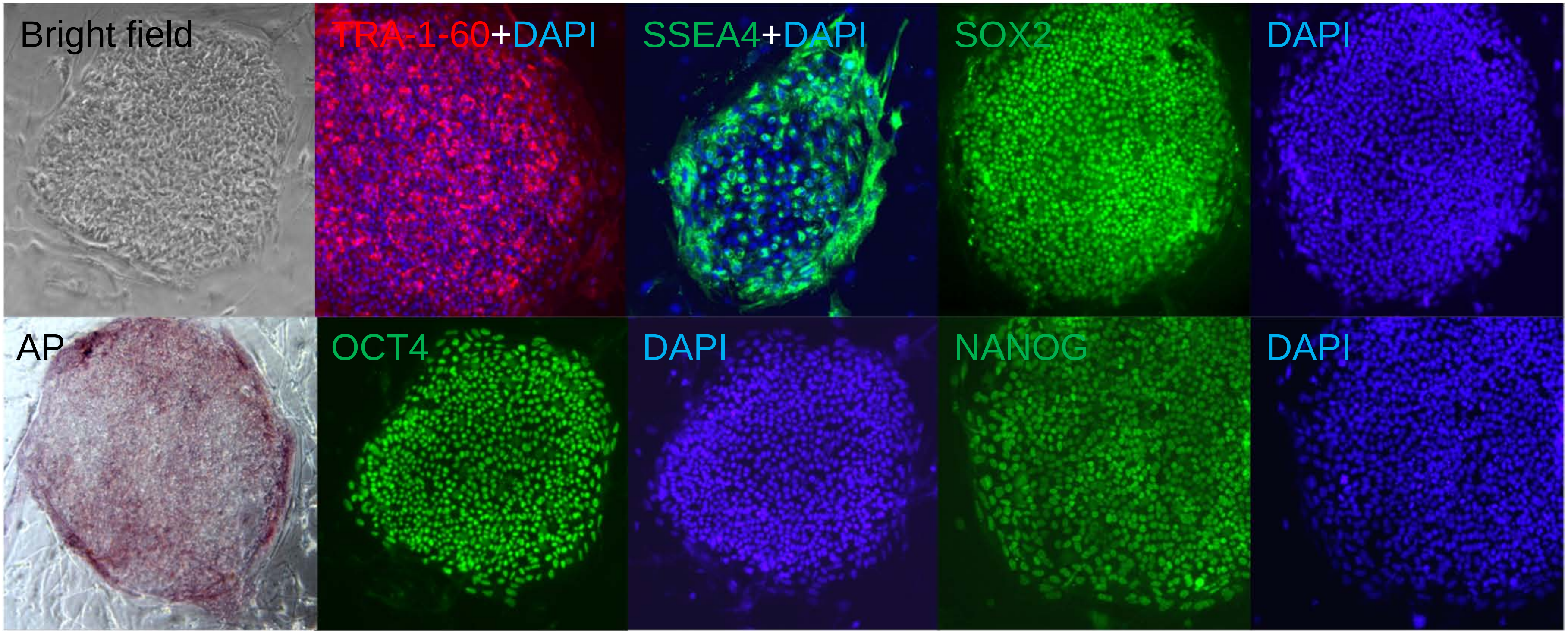

Donor Fibroblast ID# PBD643

PBD_PEX12 iPS1

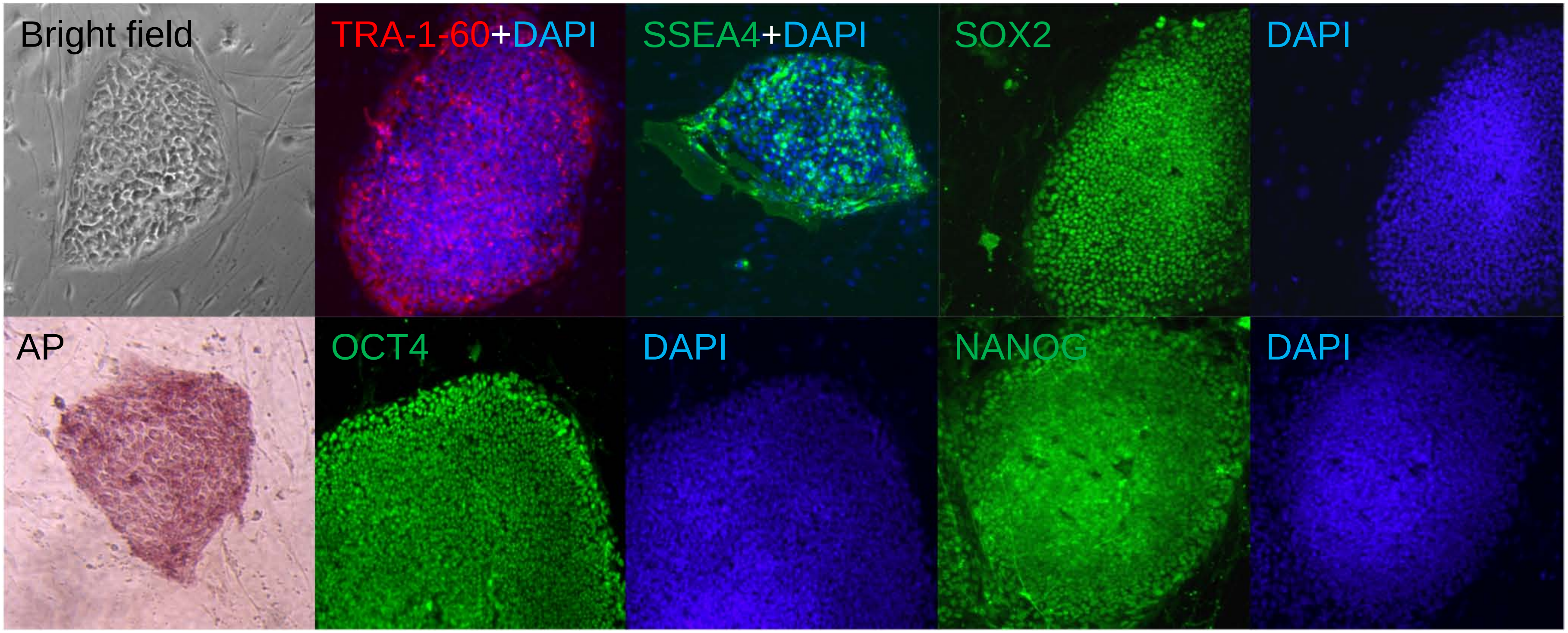

Donor Fibroblast ID# PBD673

PBD_PEX10 iPS2

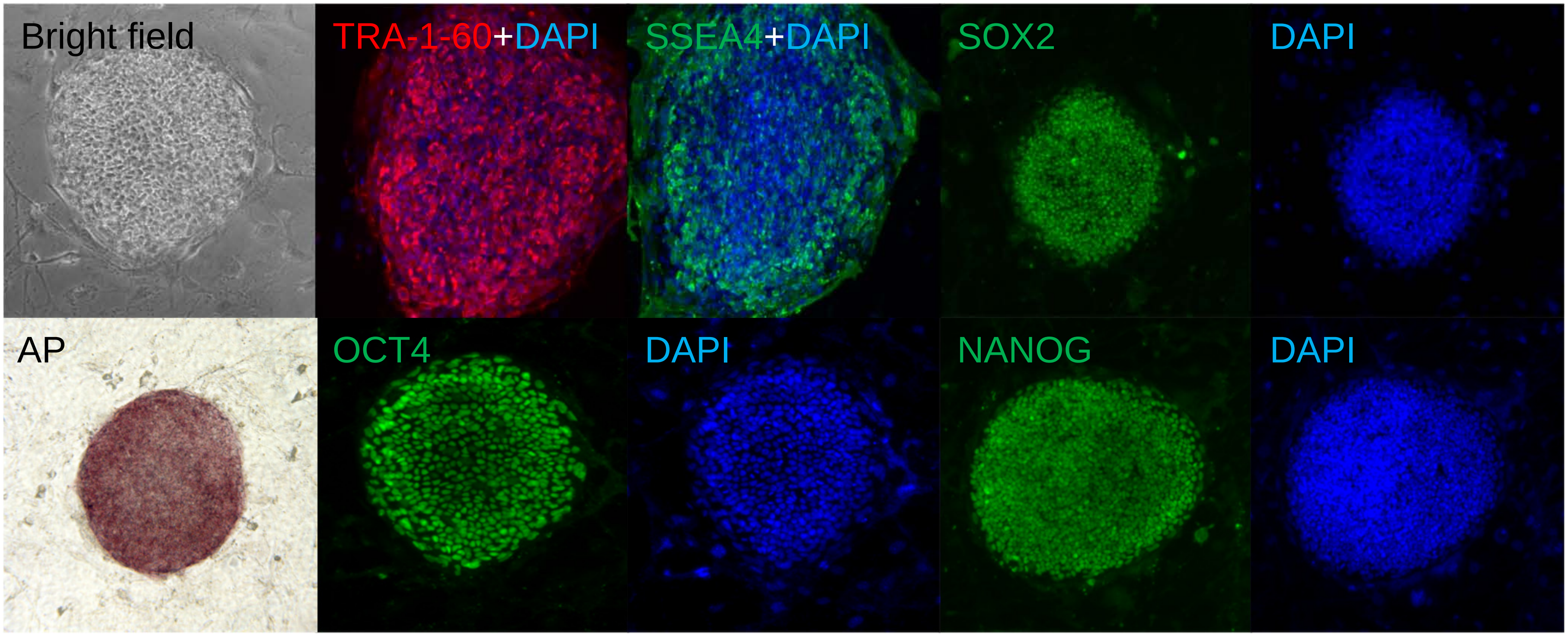

Donor Fibroblast ID# PBD687

PBD_PEX12 iPS2

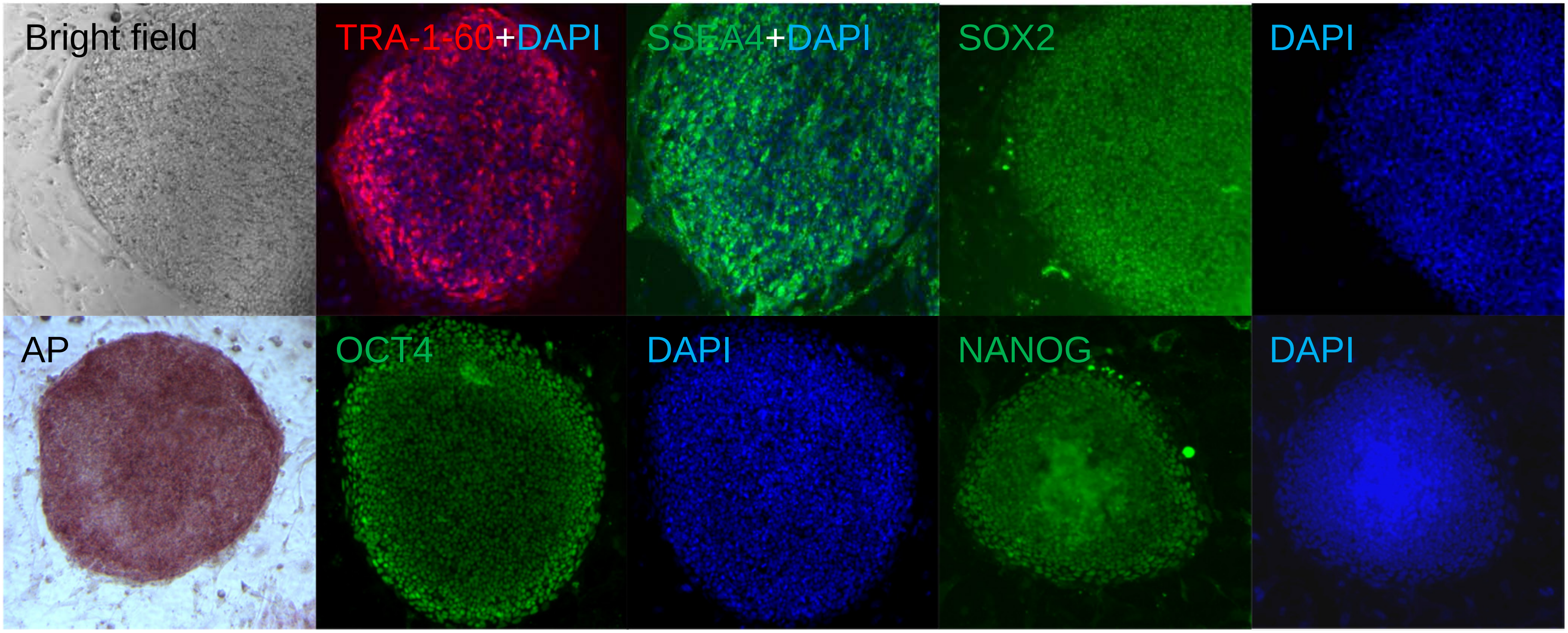

Donor Fibroblast ID# PBD673

PBD_PEX12 iPS3

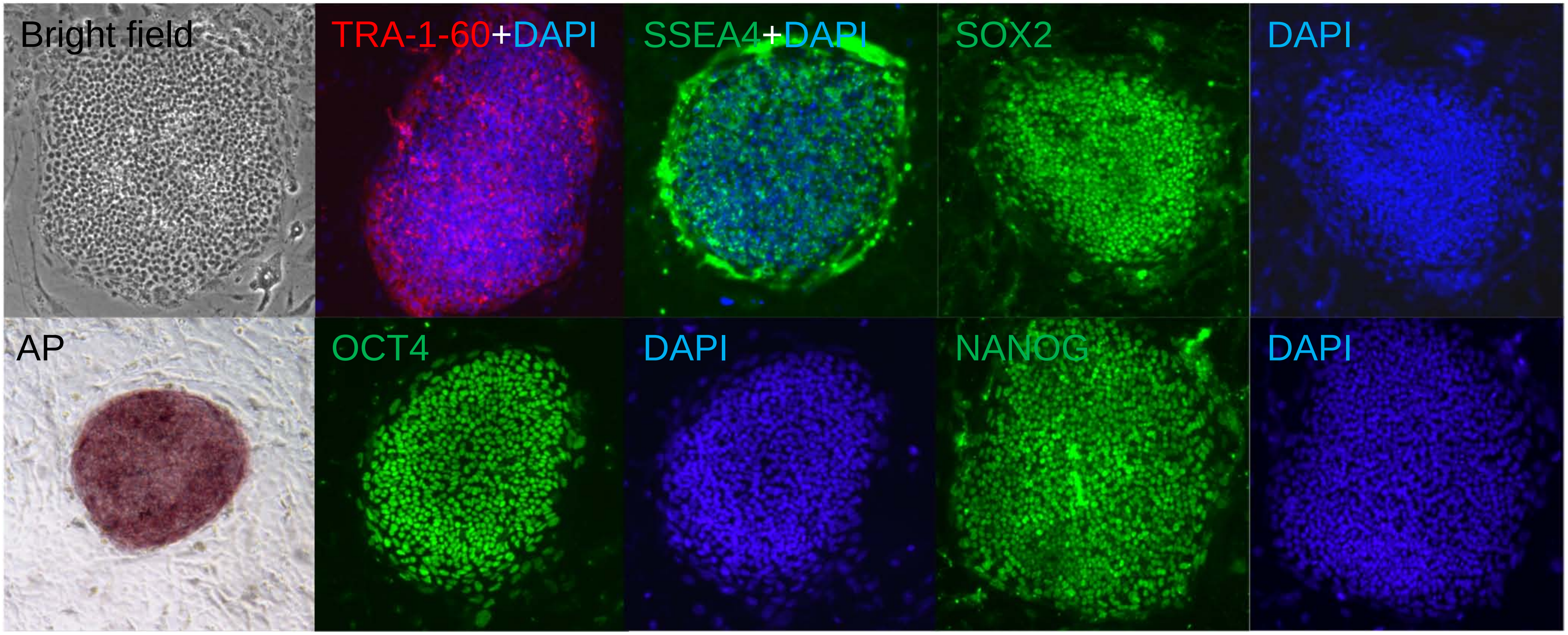

Donor Fibroblast ID# PBD673

PBD_PEX26 iPS1

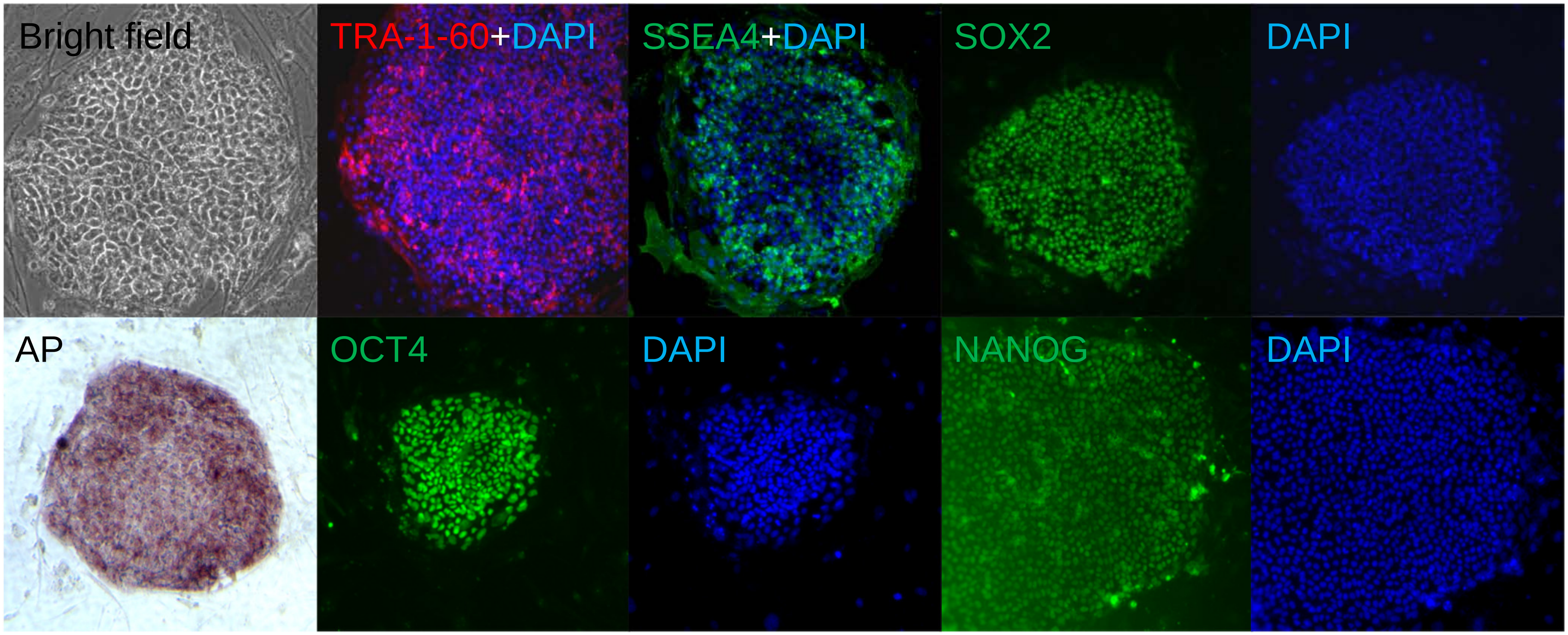

Donor Fibroblast ID# PBD604

PBD_PEX26 iPS2

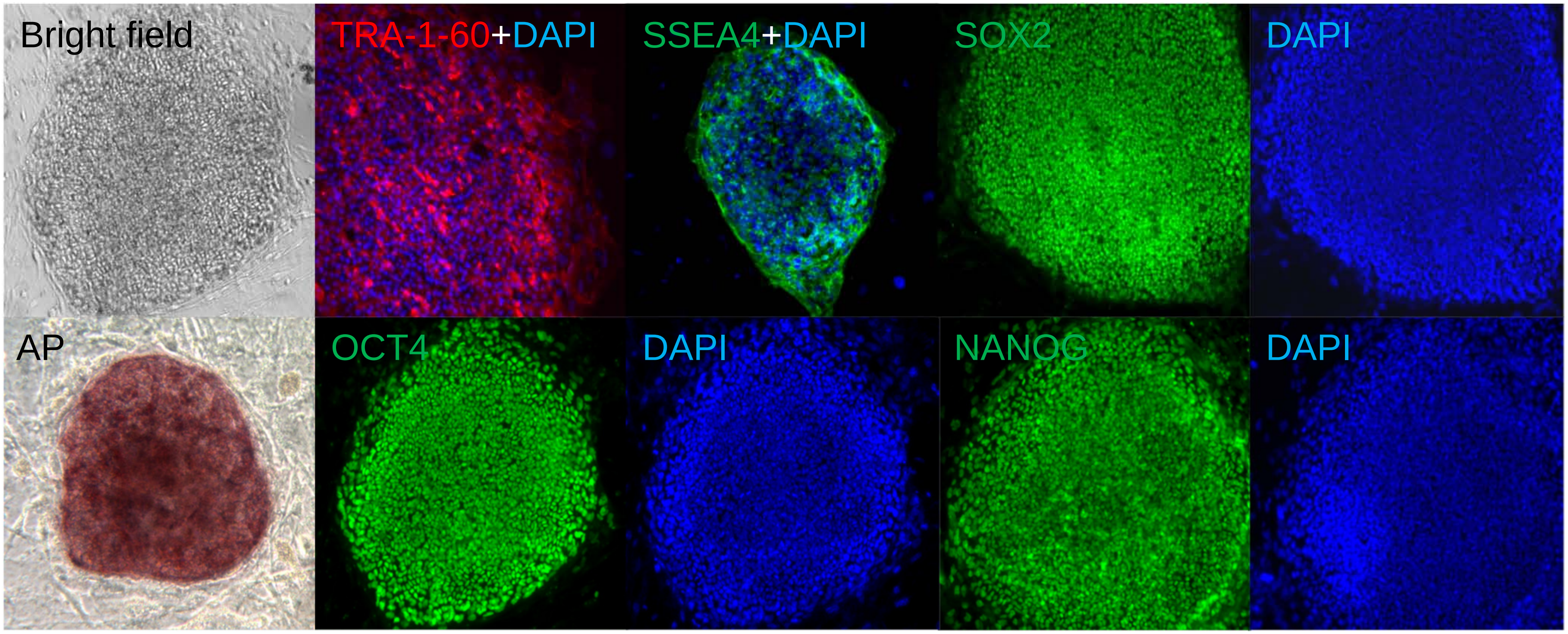

Donor Fibroblast ID# PBD604
