# Supplementary figures and images for: Induced pluripotent stem cell models of Zellweger spectrum disorder show impaired peroxisome assembly and cell type-specific lipid abnormalities
Source: Stem Cell Res Ther. 2015 Aug 29;6:158. doi: 10.1186/s13287-015-0149-3 (PMC4553005; doi:10.1186/s13287-015-0149-3)

## Slide 1
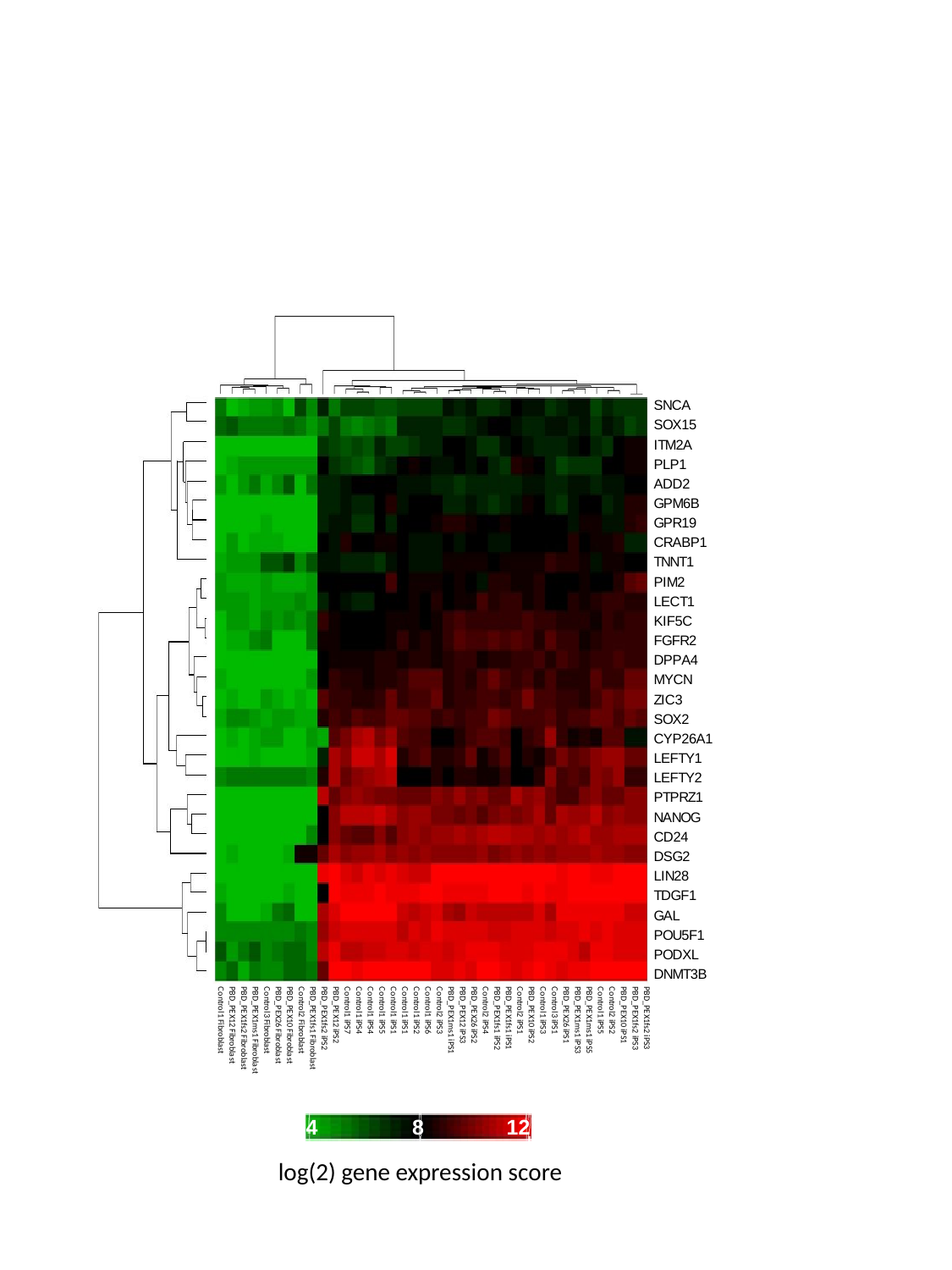

4 8 12
log(2) gene expression score

Supplement: Additional file 7: — Hierarchical clustering analysis of data from genes related to pluripotency from PBD-ZSD patient and control fibroblasts and iPSCs. This analysis was based on gene expression data from 30 pluripotency genes reported in reference [44]. (PPTX 934 kb) [file 13287_2015_149_MOESM7_ESM.pptx]
