# Supplementary material for: Induced pluripotent stem cell models of Zellweger spectrum disorder show impaired peroxisome assembly and cell type-specific lipid abnormalities
Source: Stem Cell Res Ther. 2015 Aug 29;6:158. doi: 10.1186/s13287-015-0149-3 (PMC4553005; doi:10.1186/s13287-015-0149-3)
Supplement: Additional file 10: — Differentiation potential of iPSCs to neural rosettes. The differentiation potential of PBD-ZSD and healthy control-derived iPSCs to neural rosettes was determined based on the percentage of attached EBs that formed neural rosettes in culture. In addition, we analyzed the number of days required for EB differentiation into neural rosettes. (PPTX 370 kb) [file 13287_2015_149_MOESM10_ESM.pptx]

## Slide 1
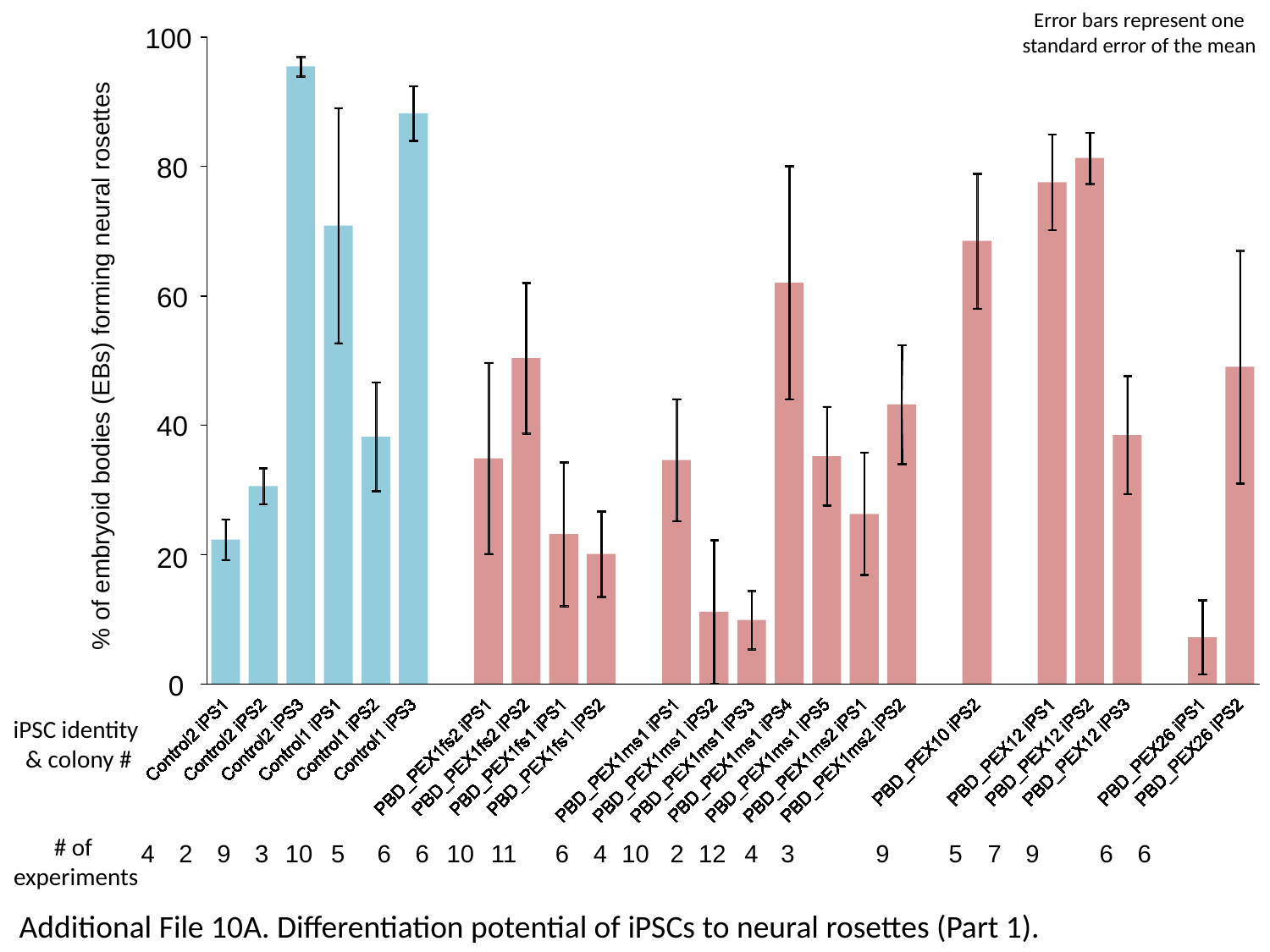

Error bars represent one
standard error of the mean
100
80
60
% of embryoid bodies (EBs) forming neural rosettes
40
20
0
iPSC identity
& colony #
# of
experiments
4
2
9
3
10
5
6
6
10
11
6
4
10
2
12
4
3
9
5
7
9
6
6
Additional File 10A. Differentiation potential of iPSCs to neural rosettes (Part 1).

## Slide 2
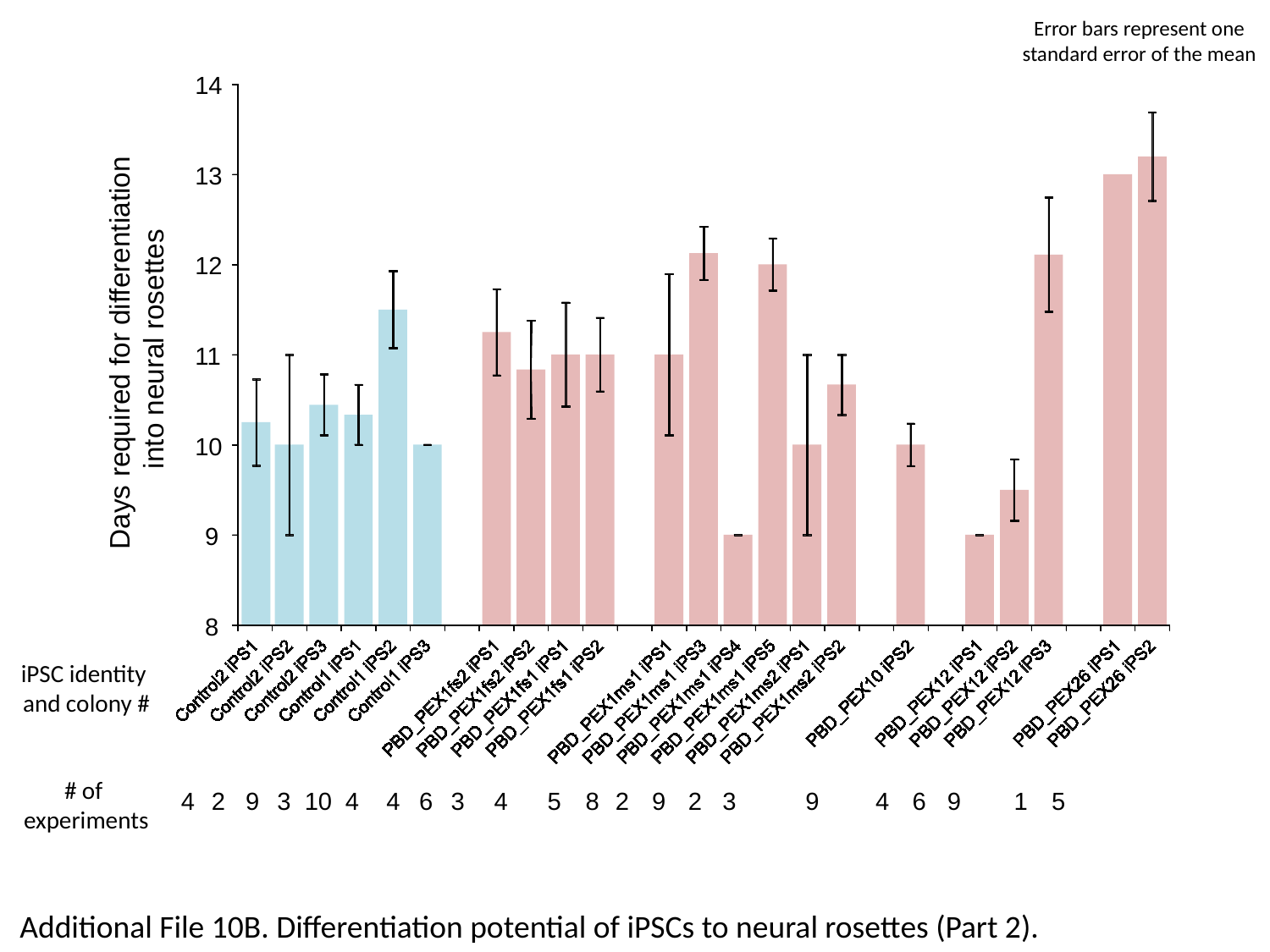

Error bars represent one
standard error of the mean
14
13
12
Days required for differentiation
into neural rosettes
11
10
9
8
iPSC identity
and colony #
# of
experiments
4
2
9
3
10
4
4
6
3
4
5
8
2
9
2
3
9
1
5
4
6
9
Additional File 10B. Differentiation potential of iPSCs to neural rosettes (Part 2).
