# Supplementary material for: Induced pluripotent stem cell models of Zellweger spectrum disorder show impaired peroxisome assembly and cell type-specific lipid abnormalities
Source: Stem Cell Res Ther. 2015 Aug 29;6:158. doi: 10.1186/s13287-015-0149-3 (PMC4553005; doi:10.1186/s13287-015-0149-3)
Supplement: Additional file 11: — Differentiation potential of iPSCs to neural rosettes. Representative images of healthy control and PBD-ZSD patient-derived cells in various stages of neural differentiation. (PDF 11822 kb) [file 13287_2015_149_MOESM11_ESM.pdf]

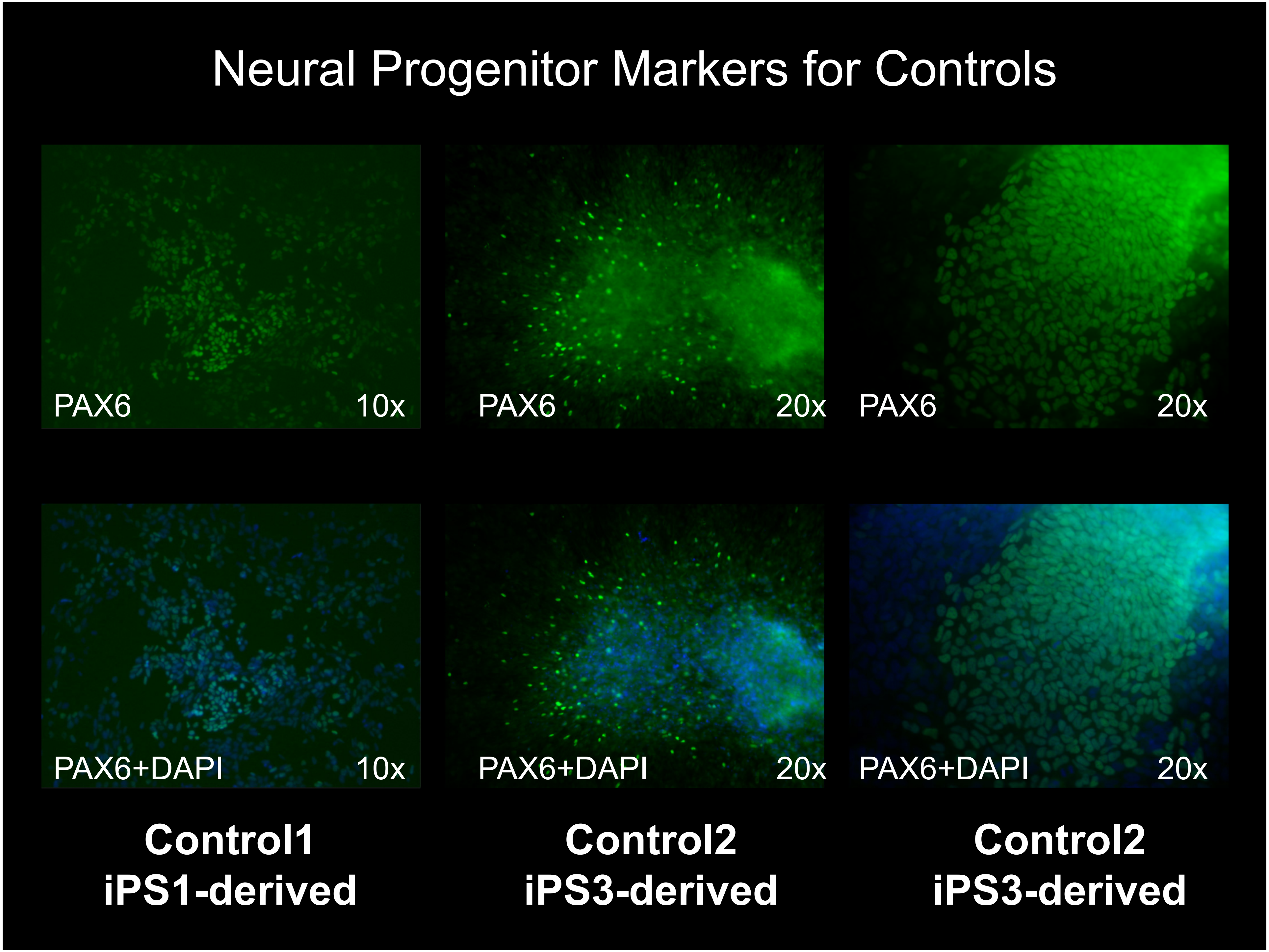
Neural Progenitor Markers for Controls
PAX6
10x
PAX6
20x
PAX6
20x
PAX6+DAPI
10x
PAX6+DAPI
20x
PAX6+DAPI
20x
Control1
iPS1-derived
Control2
iPS3-derived
Control2
iPS3-derived

Motor Neuron Progenitor Markers for Controls

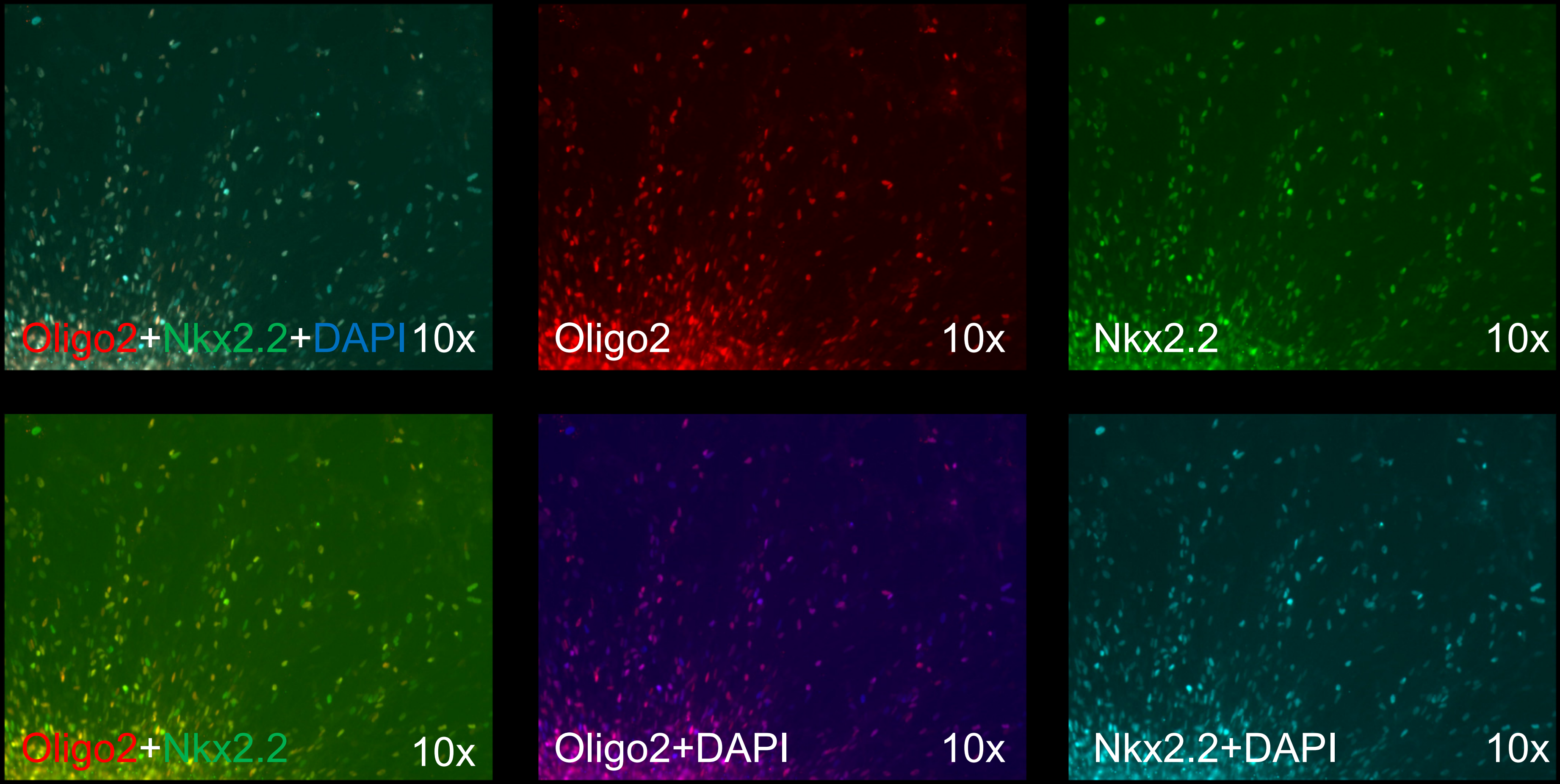

All Control1iPS1-derived

Motor Neuron Progenitor Markers for Controls

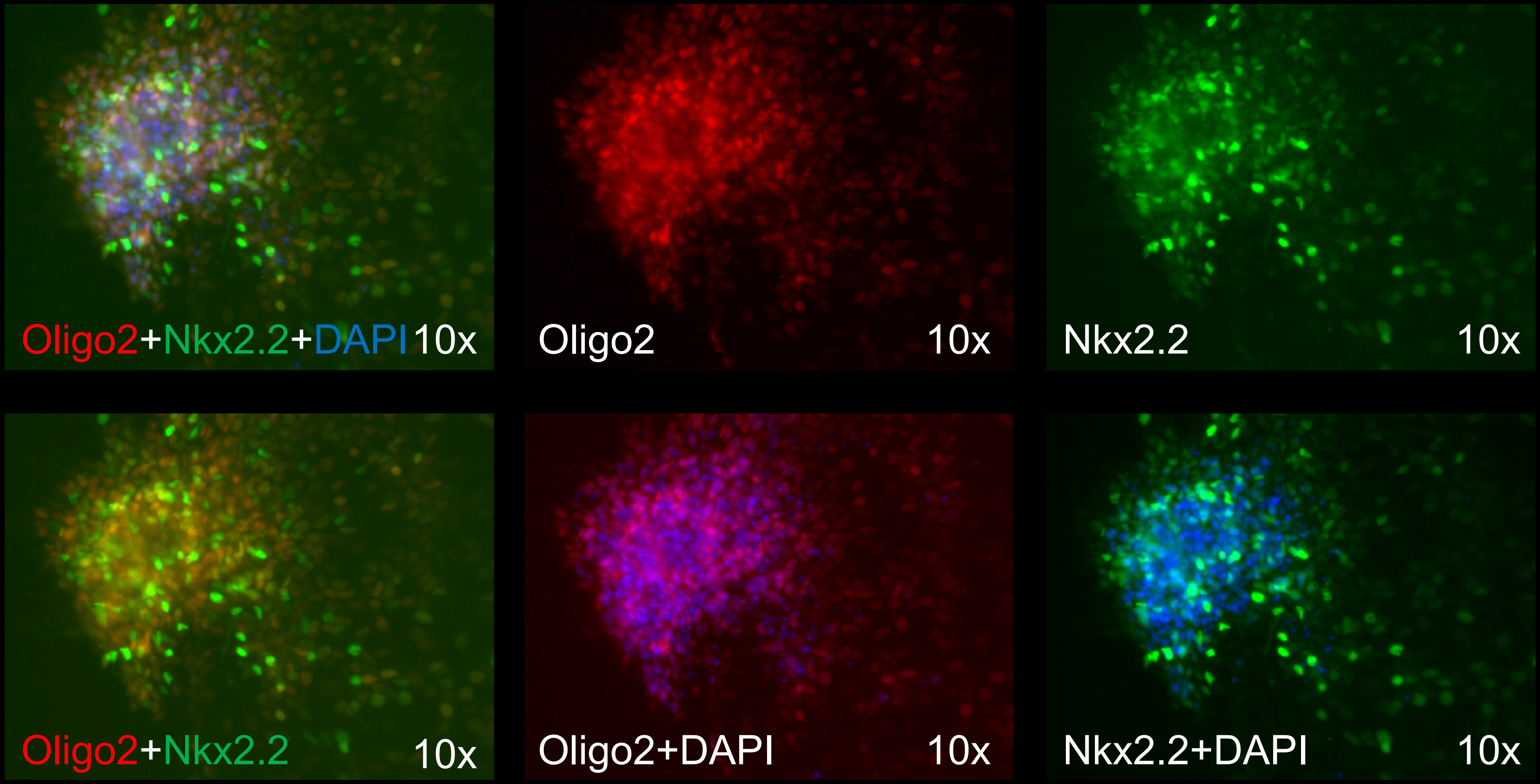

All Control2 iPS3-derived

Motor Neuron Progenitor Markers for Controls

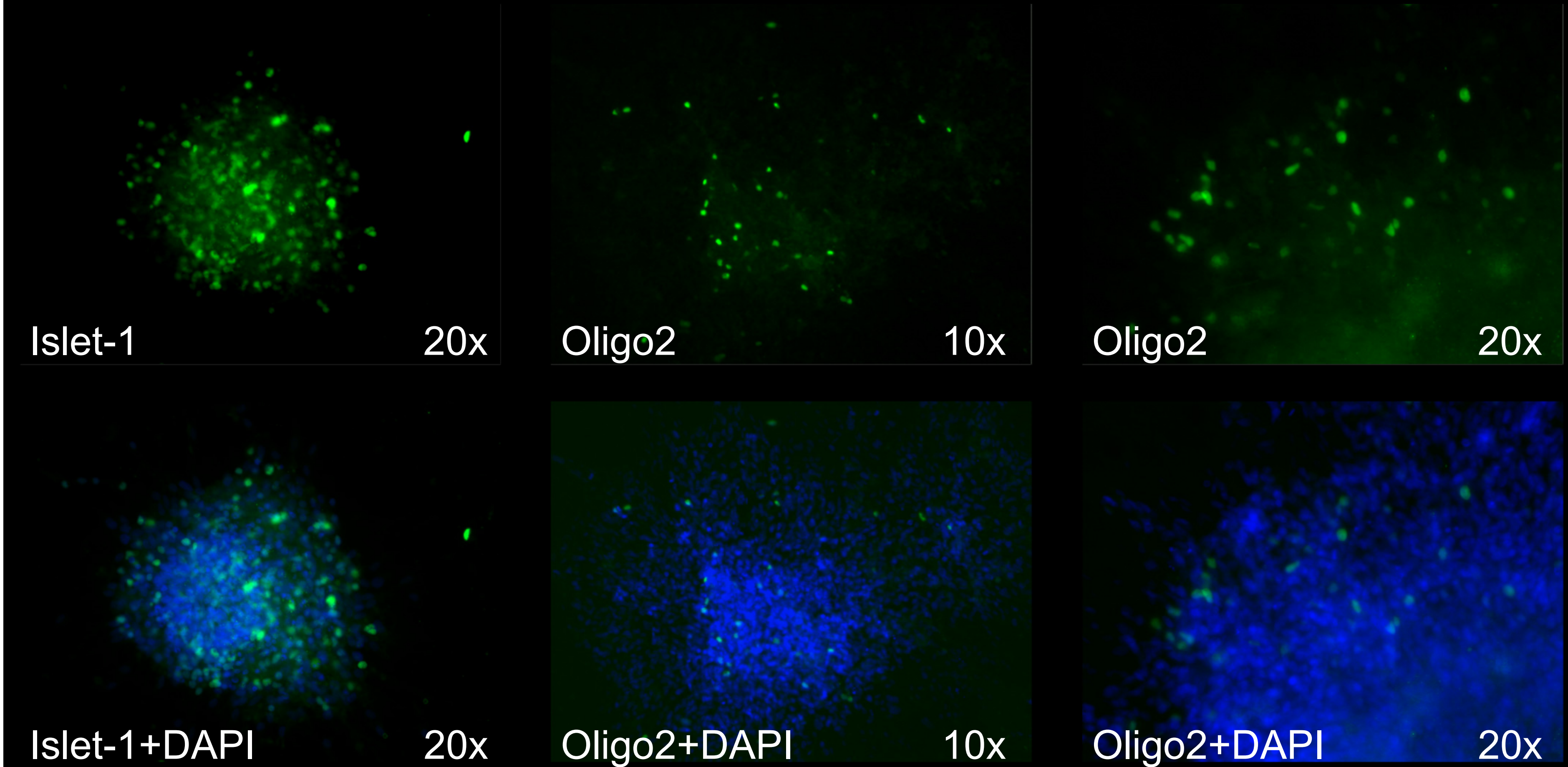

Control2 iPS1-derived

Control2 iPS3-derived

Control2 iPS3-derived

Motor Neuron Progenitor Markers for Controls

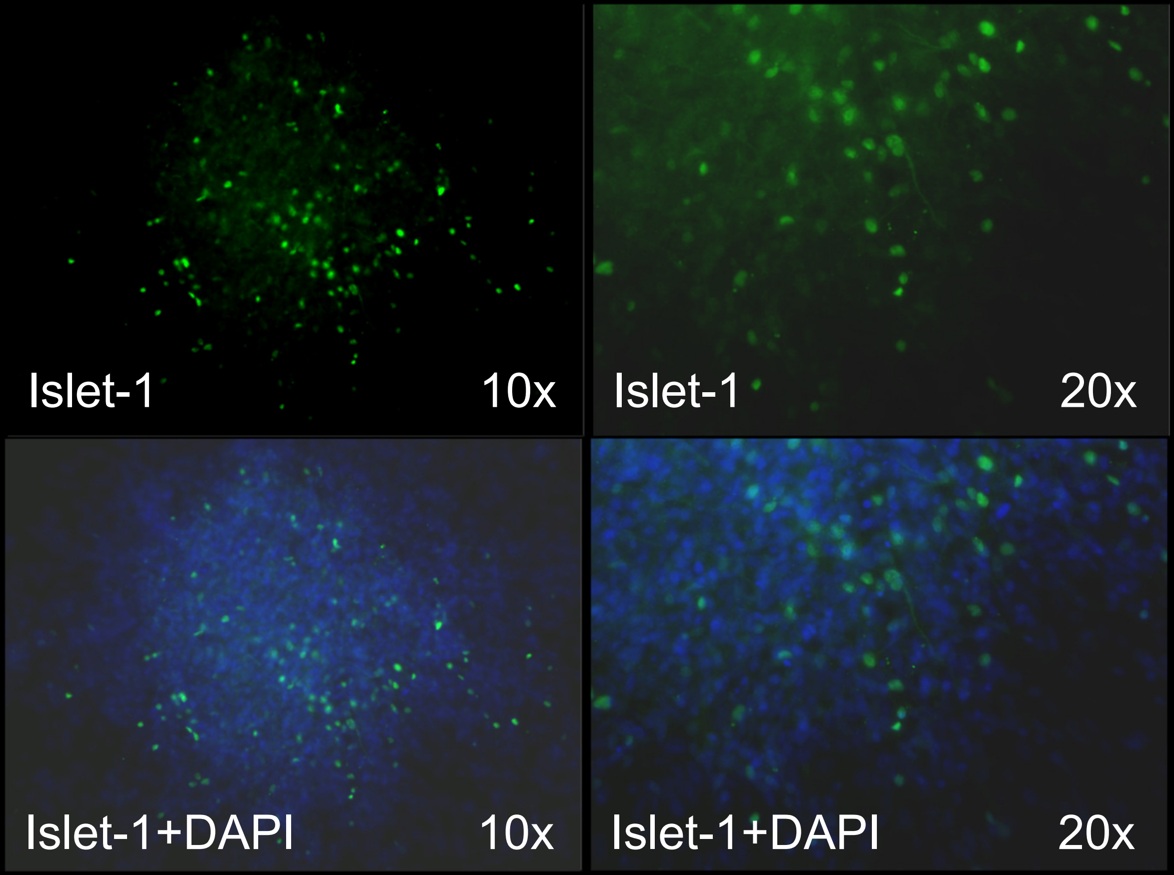

All Control2 iPS3-derived

Neuron Markers for Controls

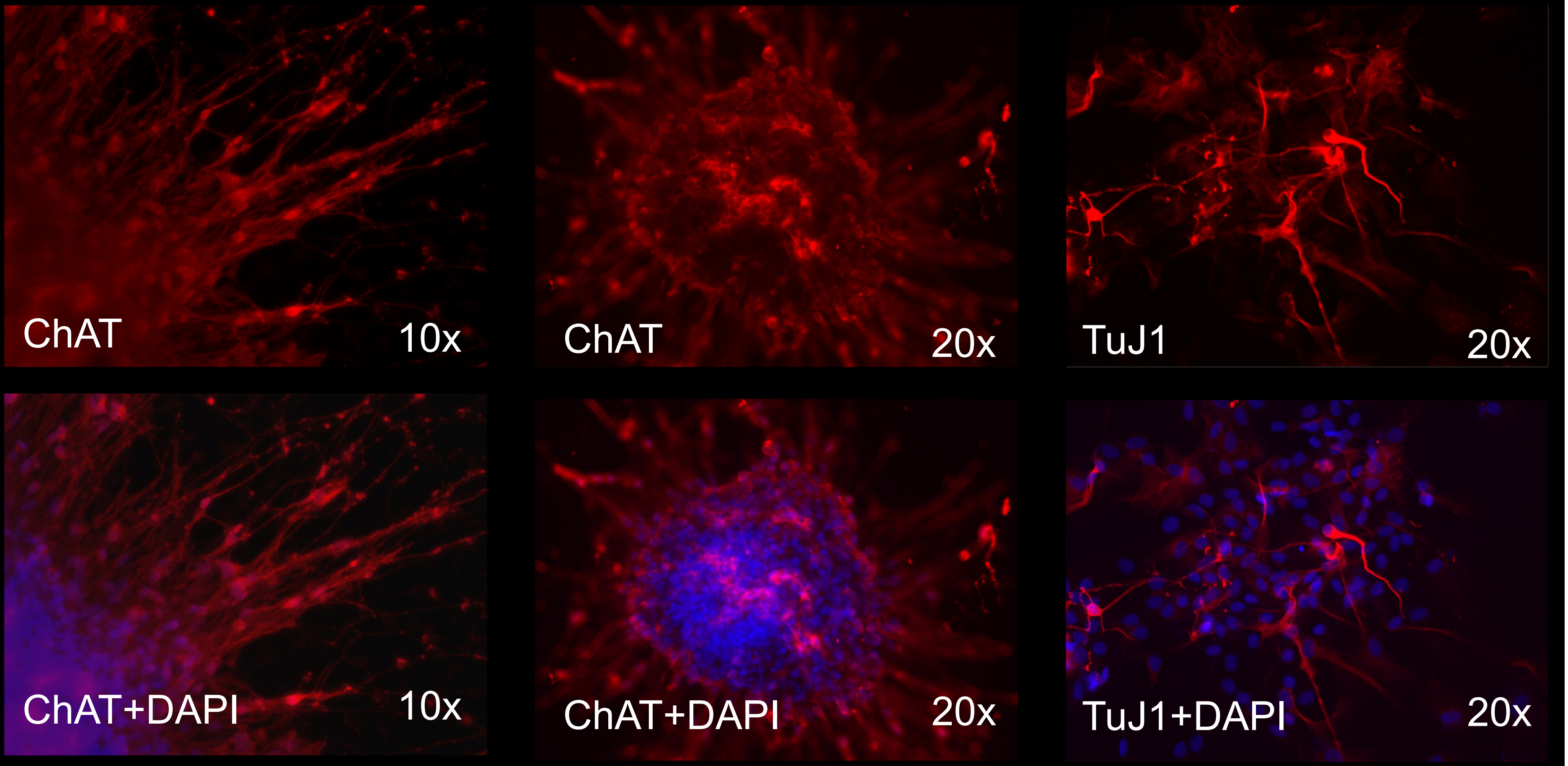

All are Control2 iPS1-derived

Neuron Markers for Controls

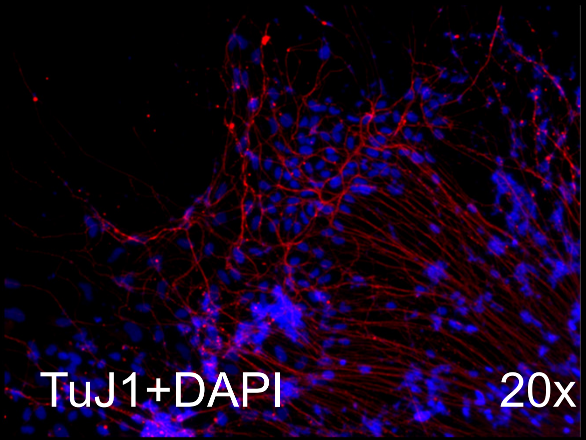

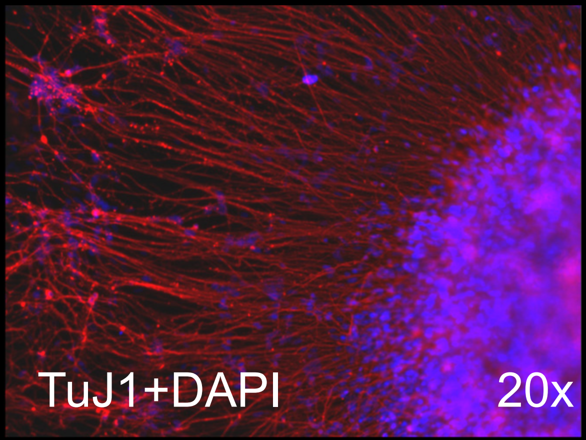

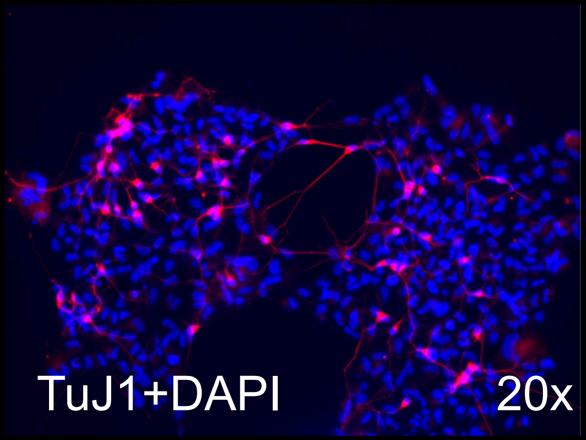

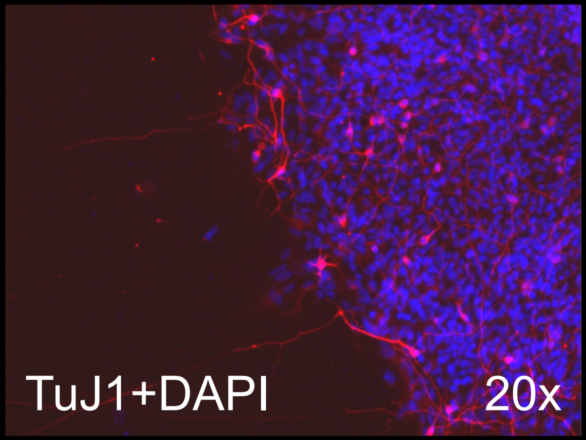

All are Control2 iPS3-derived

Neuron Markers for Controls

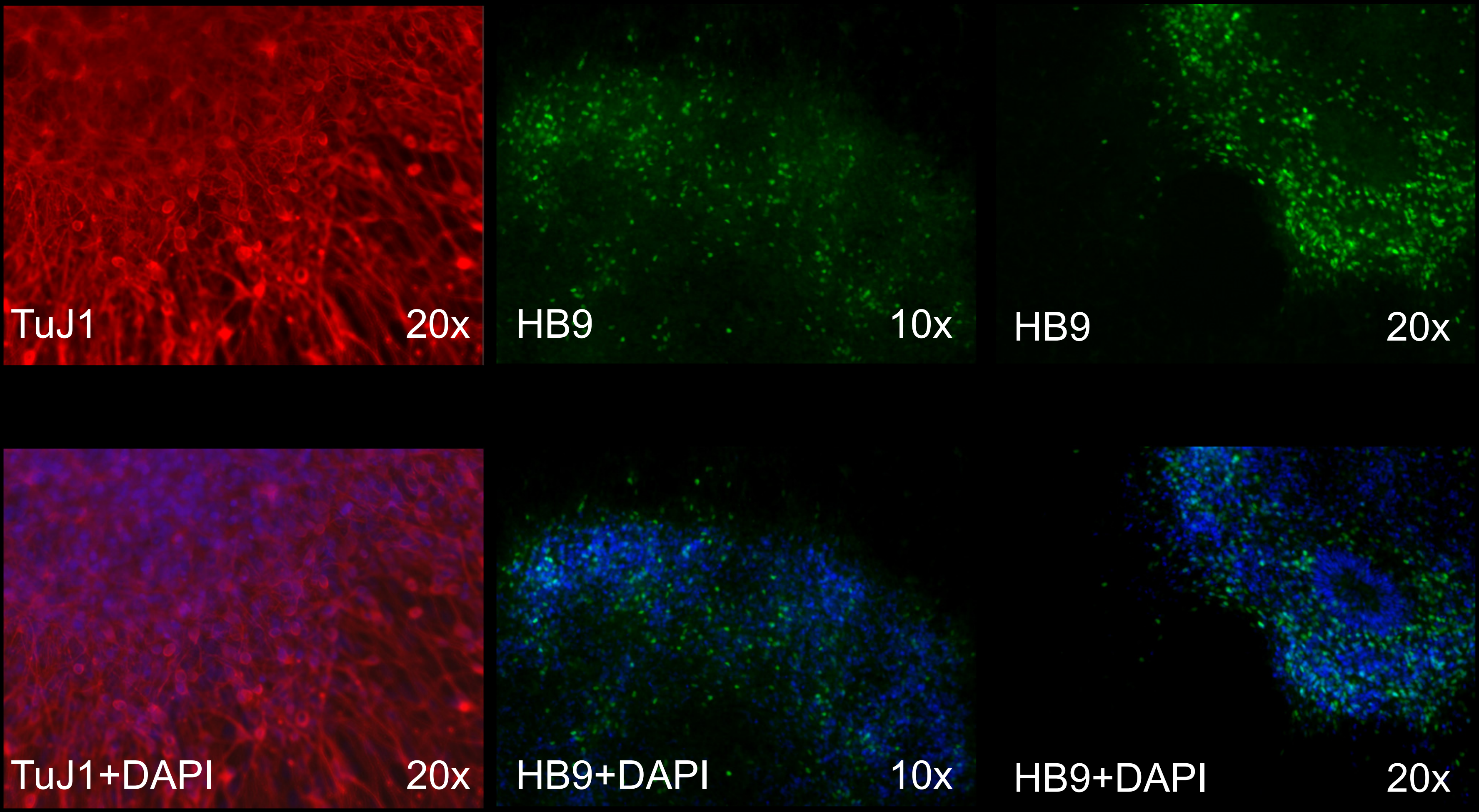

All are Control2 iPS3-derived

Neuron Markers for Controls

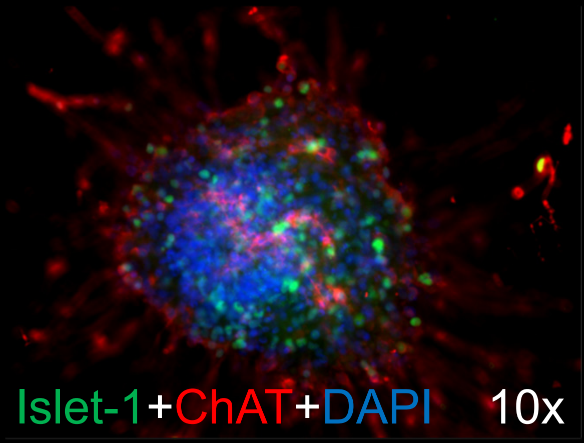

Control2 iPS1-derived

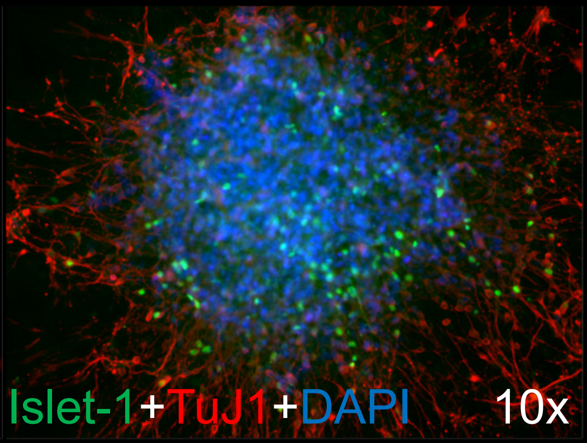

Control2 iPS3-derived

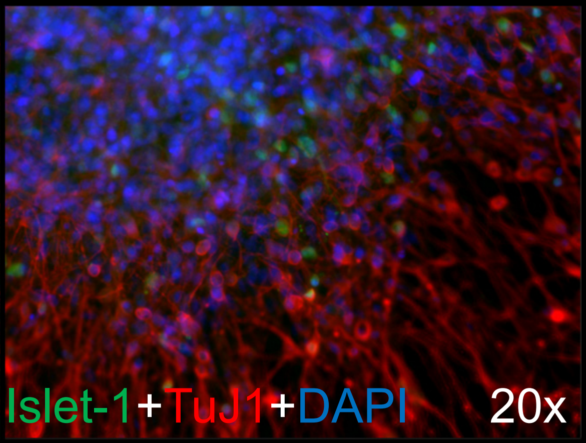

Control2 iPS3-derived

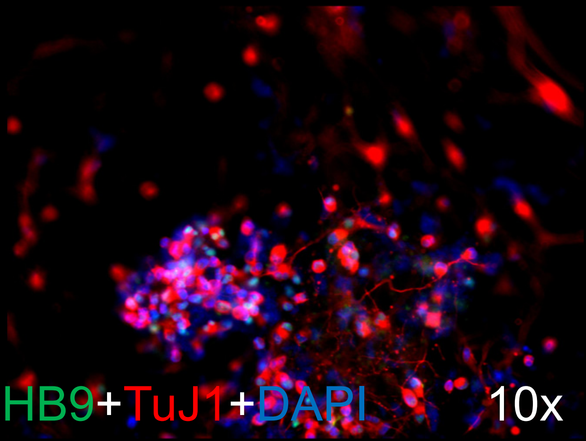

Control2 iPS3-derived

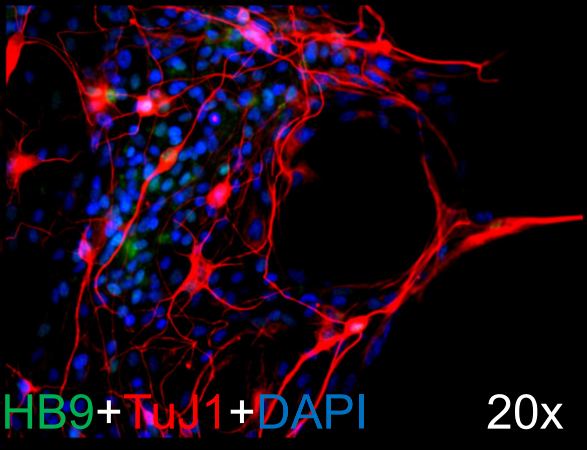

Control2 iPS3-derived

Neural Progenitor Markers for PBD-ZSD Patients

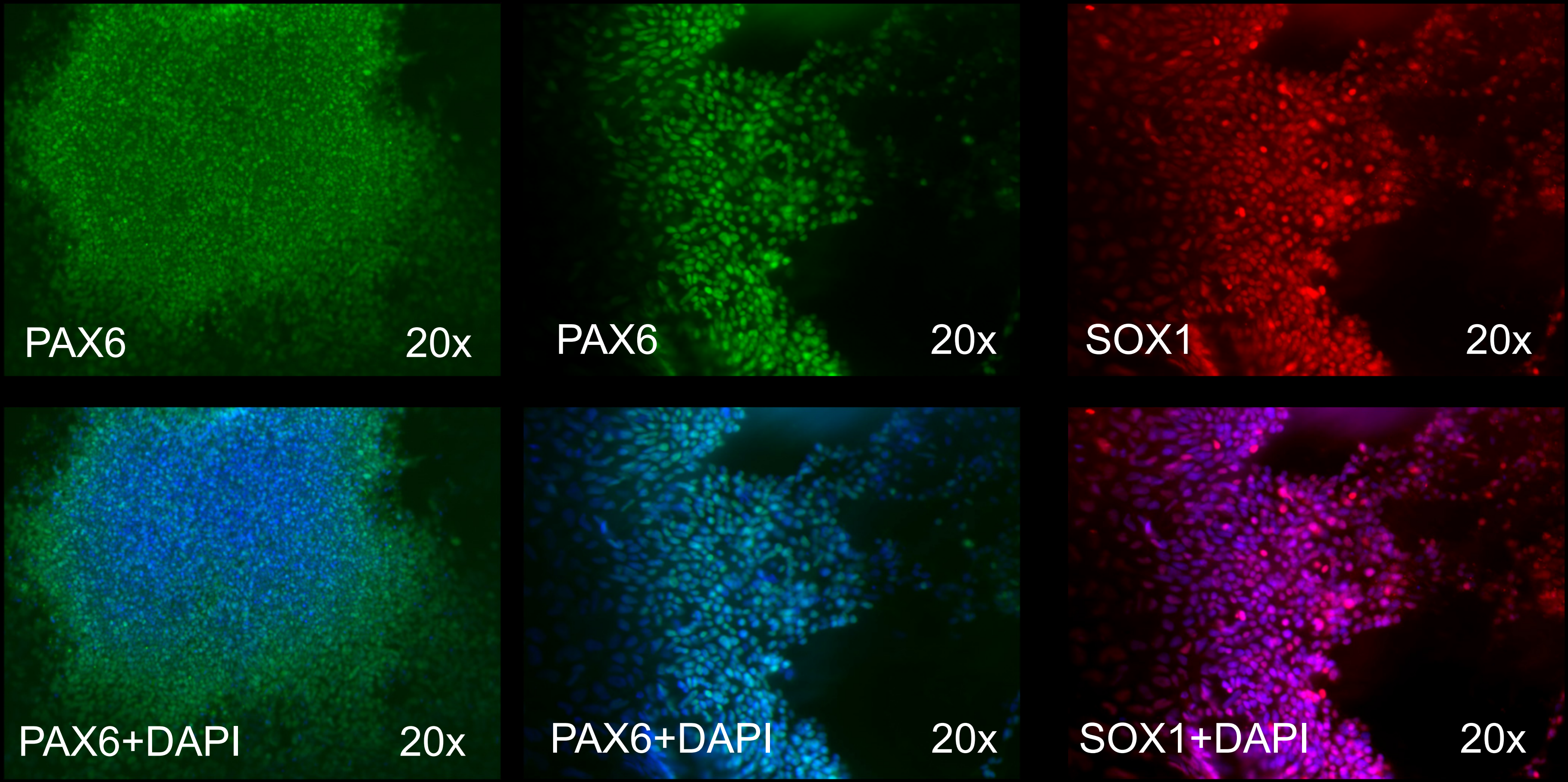

All PBD_PEX1ms1 iPS3-derived

Motor Neuron Progenitor Markers for PBD Patients

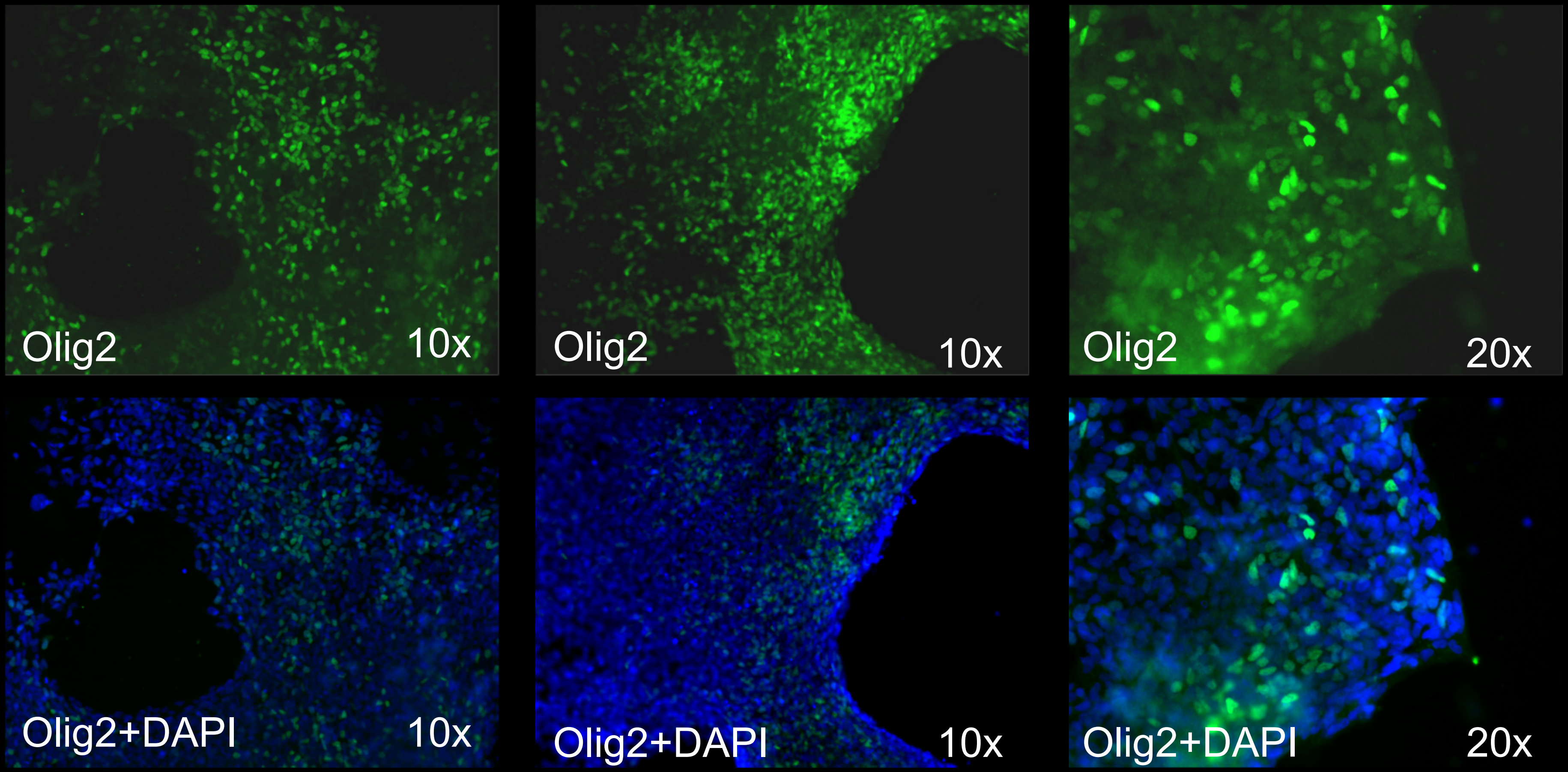

All PBD_PEX12 iPS2-derived

Motor Neuron Progenitor Markers for PBD Patients

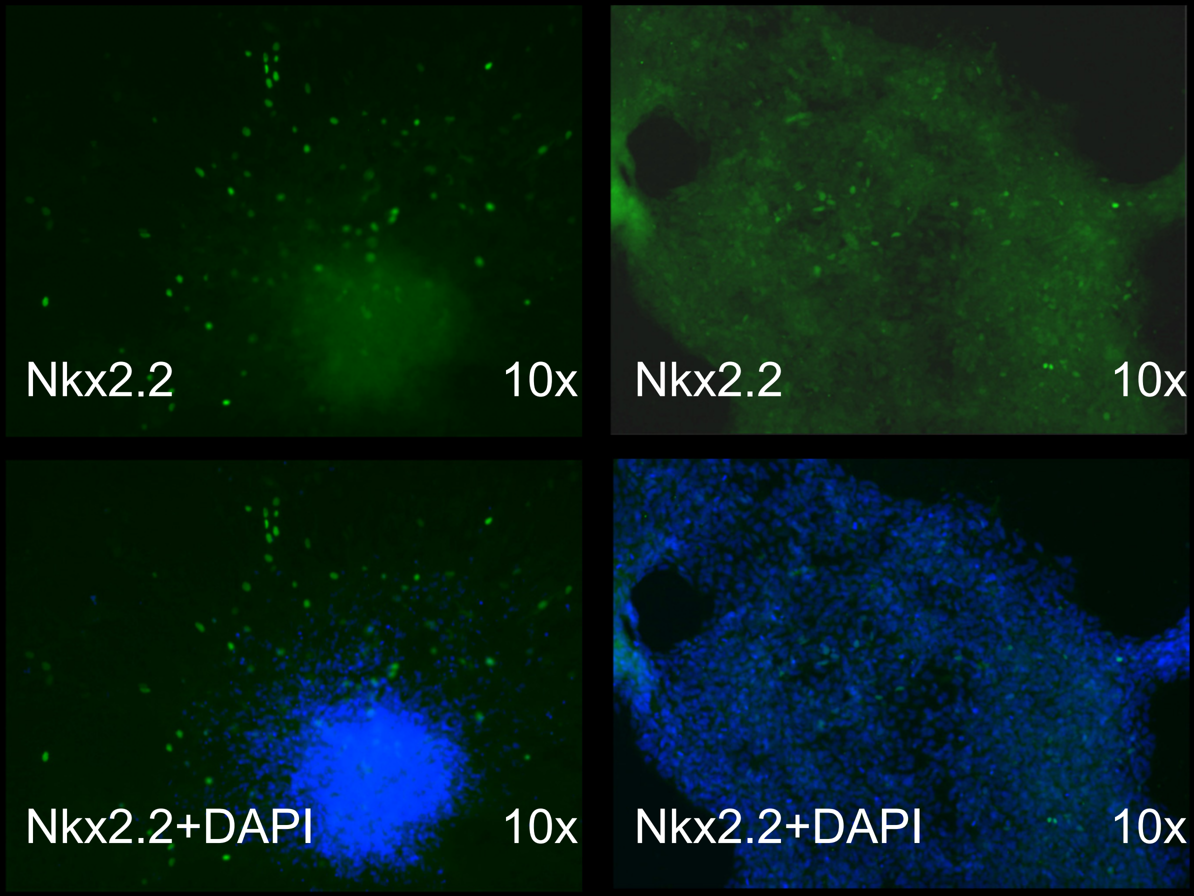

PBD_PEX1ms1
iPS1-derived

PBD_PEX12
iPS2-derived

Motor Neuron Progenitor Markers for PBD Patients

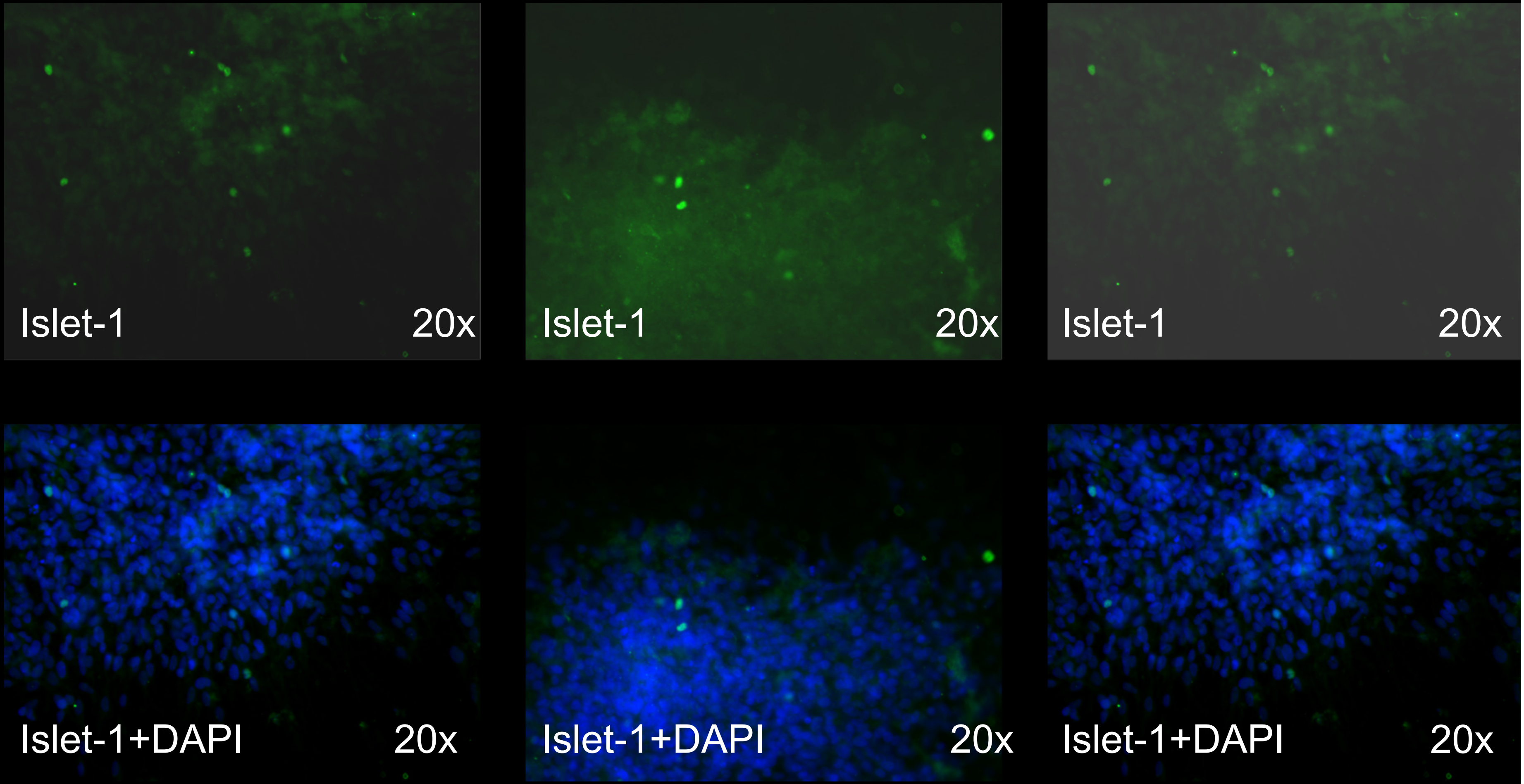

PBD_PEX1fs2
iPS2-derived

PBD_PEX1ms1
iPS2-derived

PBD_PEX1fs2
iPS2-derived

Neuron Markers for PBD patients

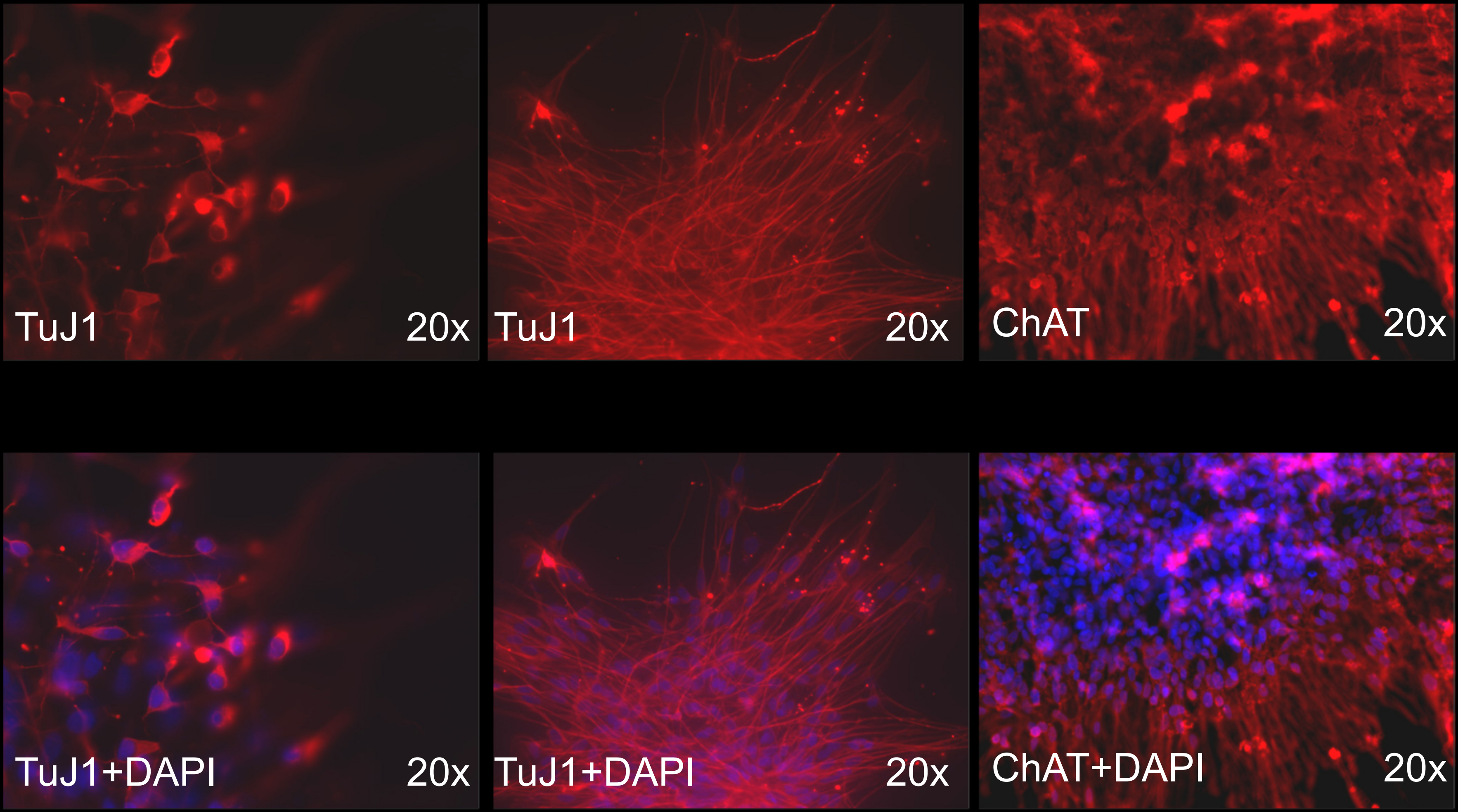

PBD_PEX1fs1
iPS2-derived

PBD_PEX1fs2
iPS2-derived

PBD_PEX1fs2
iPSC2-derived

Neuron Markers for PBD Patients

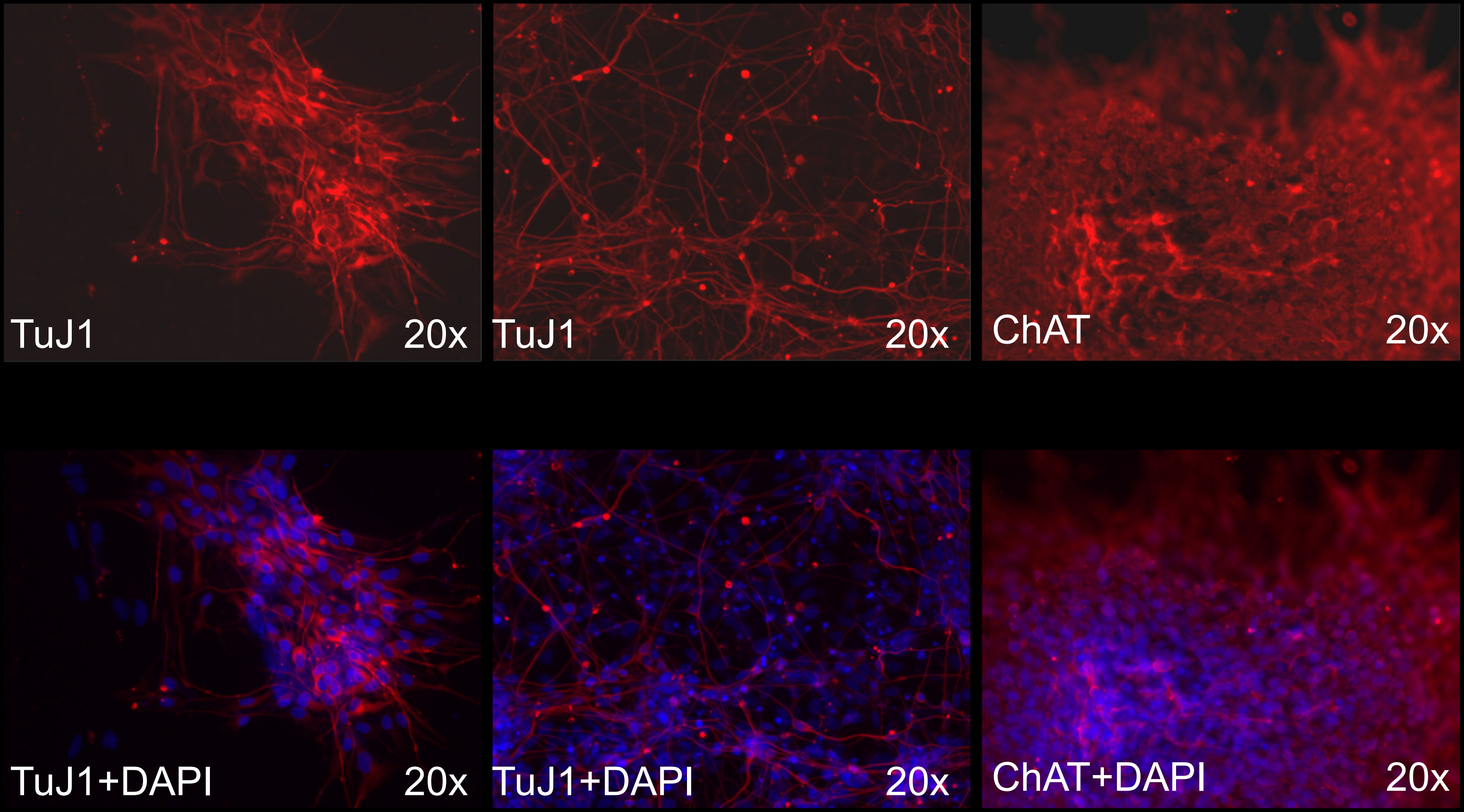

PBD_PEX1ms1
iPS2-derived

PBD_PEX1ms1
iPS1-derived

PBD_PEX1ms1
iPS2-derived

Neuron Markers for PBD Patients

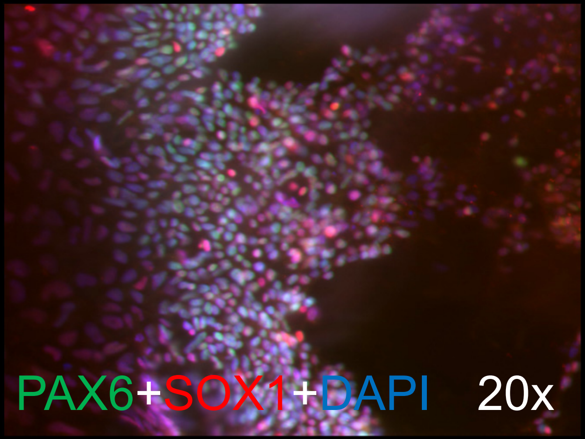

PBD_PEX1ms1
iPS3-derived

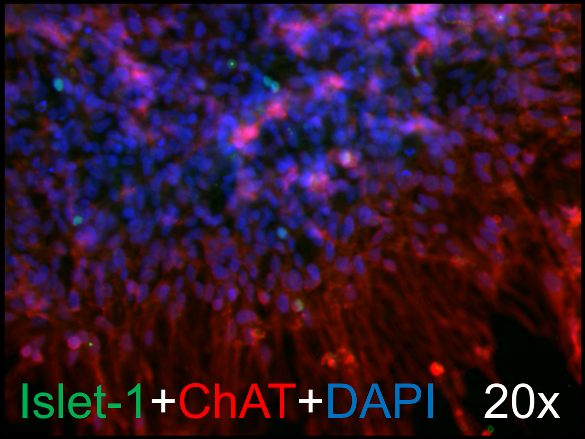

PBD_PEX1fs2
iPS1-derived

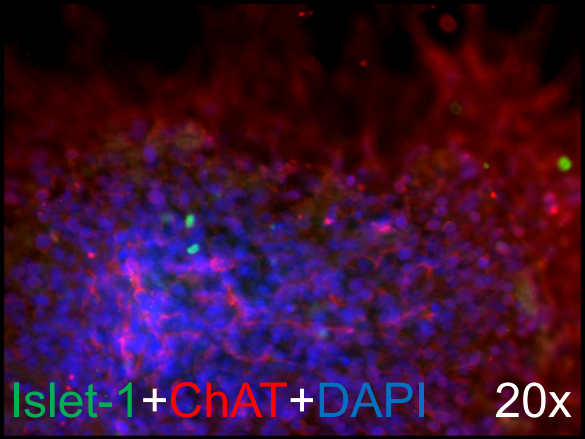

PBD_PEX1ms1
iPS2-derived

Neuroepithelial markers for PBD patients

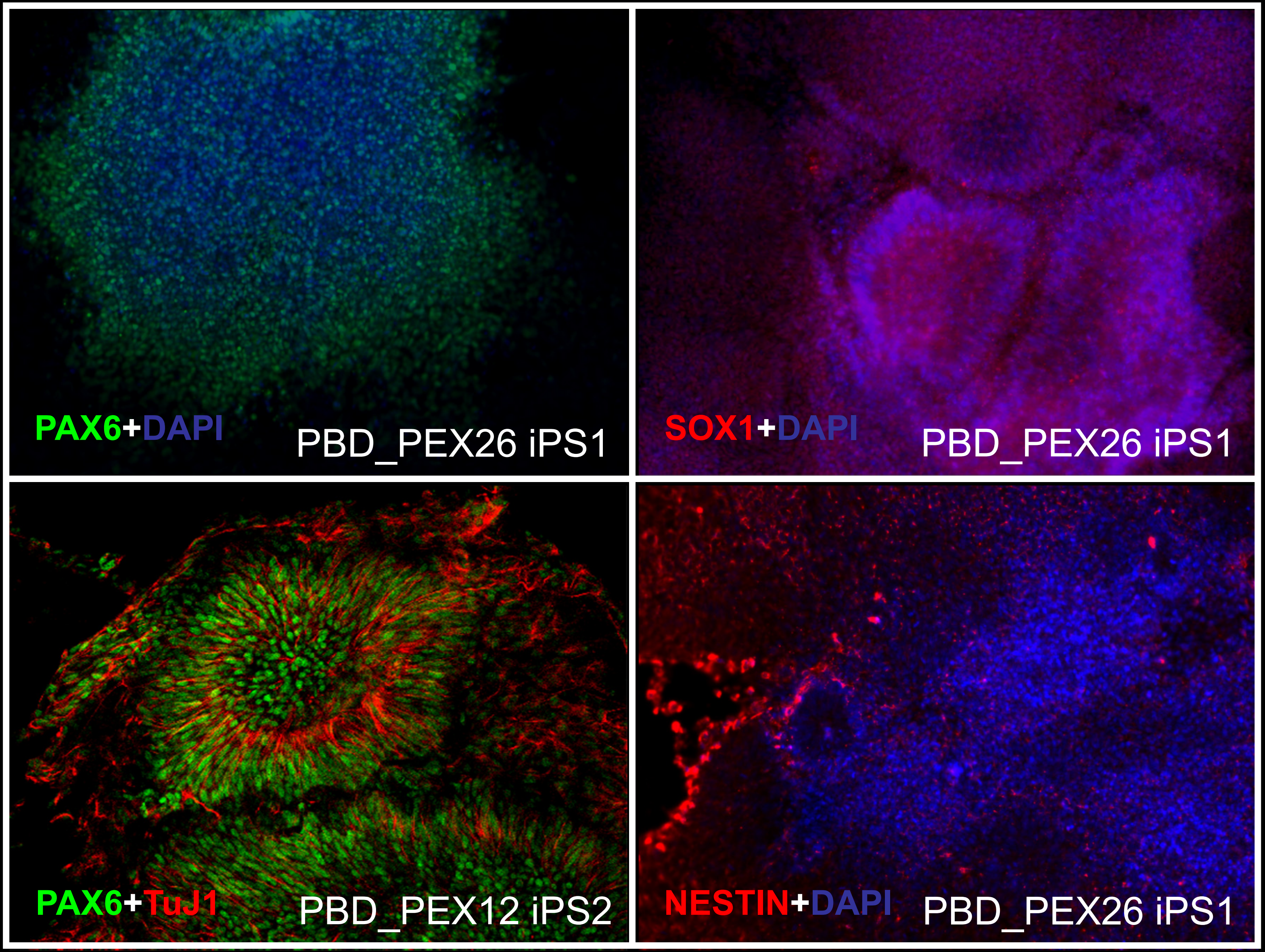

Motor neuron progenitor markers for PBD patients

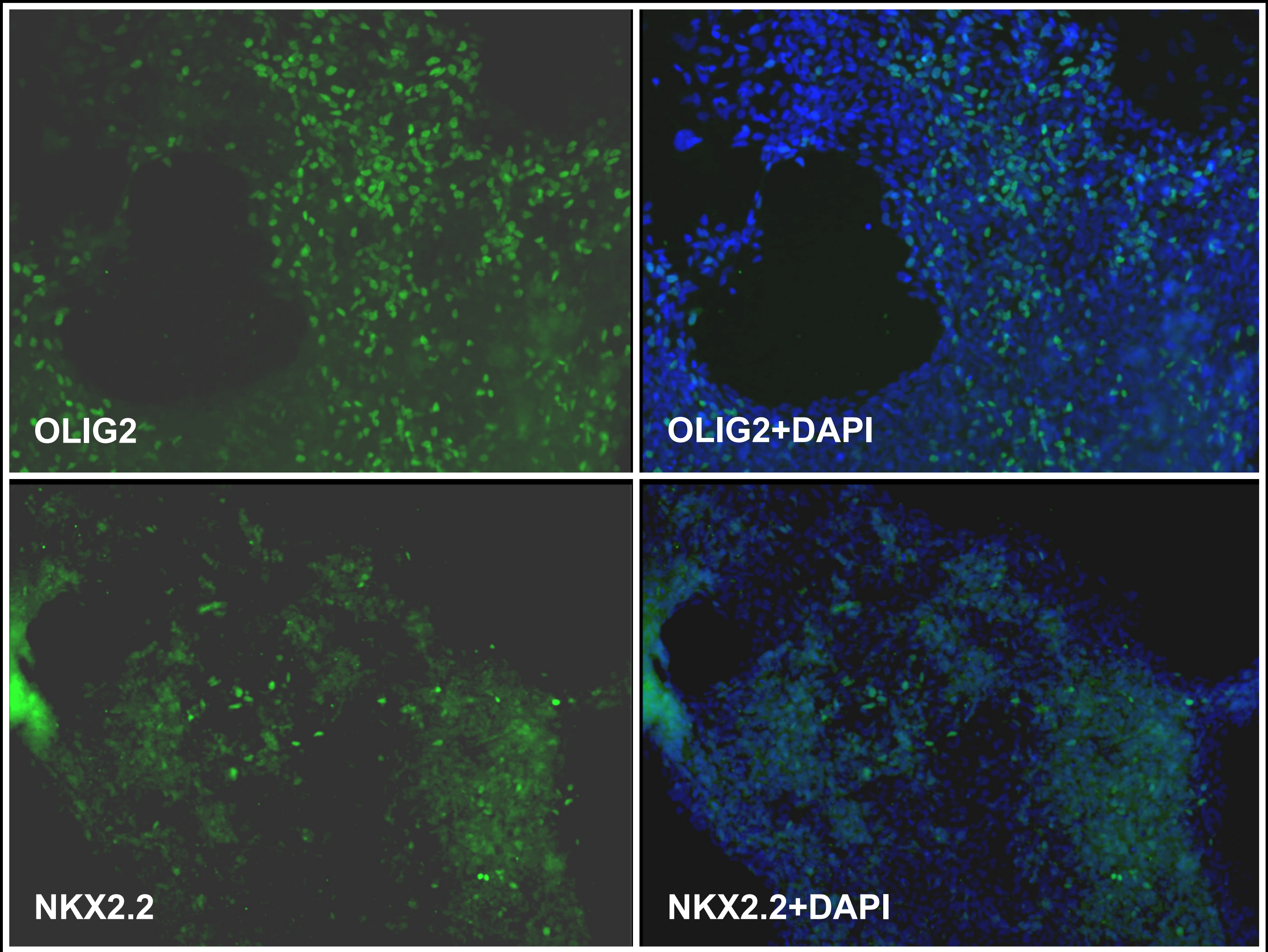

All PBD_PEX12 iPS2-derived

Motor neuron markers for Controls

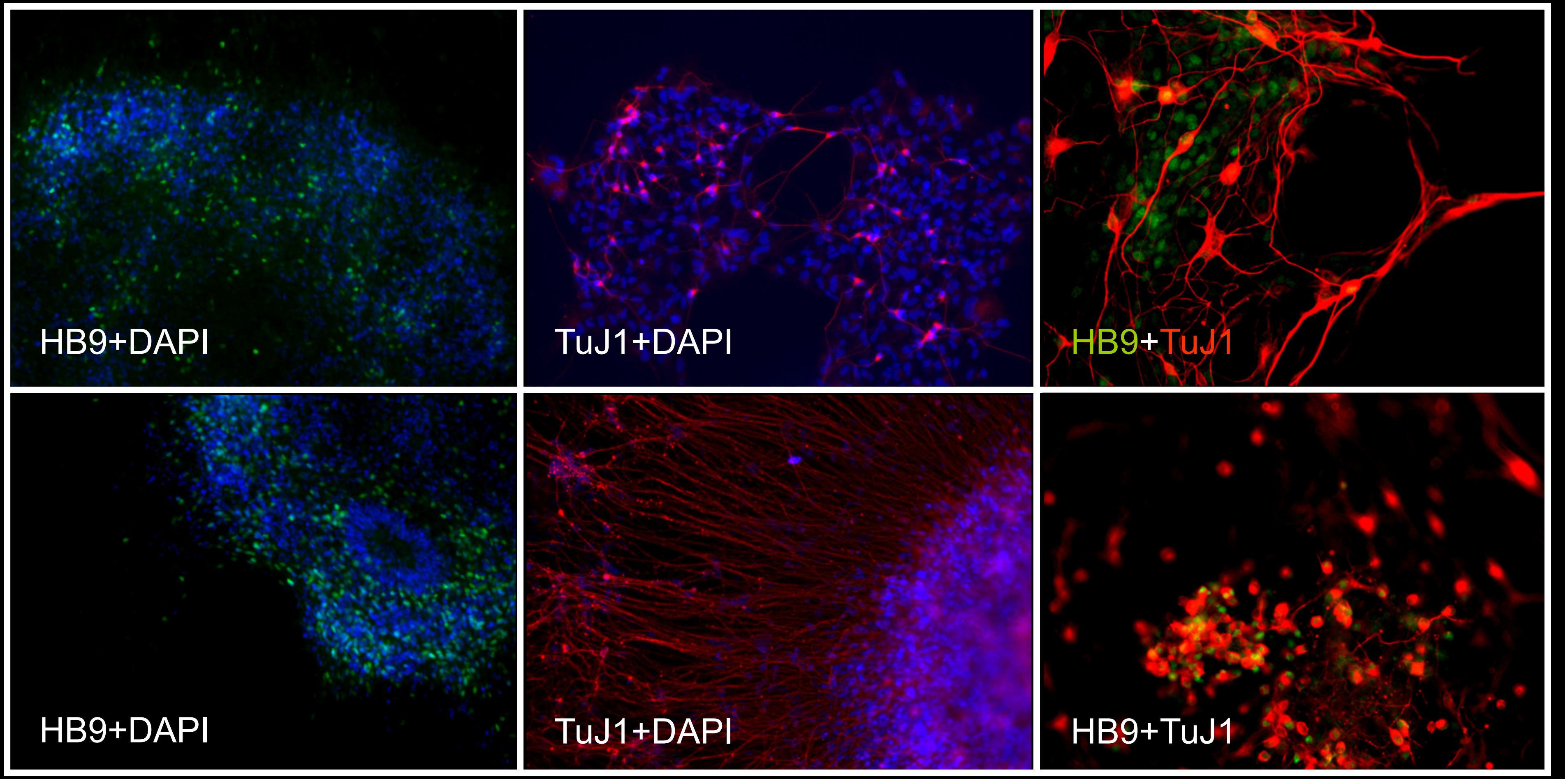

All Control2 iPS3-derived

Motor neurons transfected with HB9 driven GFP

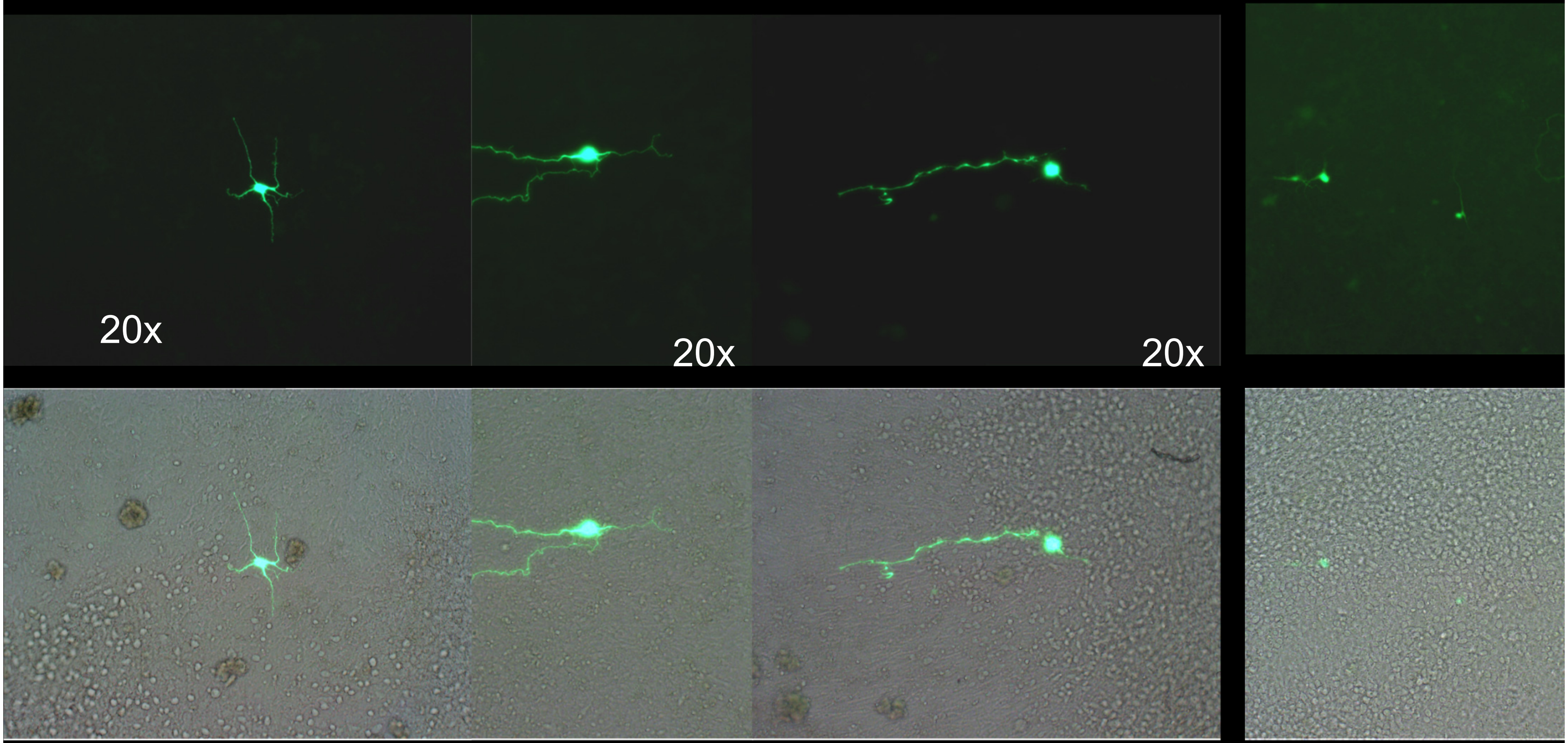

Control2 iPS3-derived

Motor neurons transfected with HB9 driven GFP

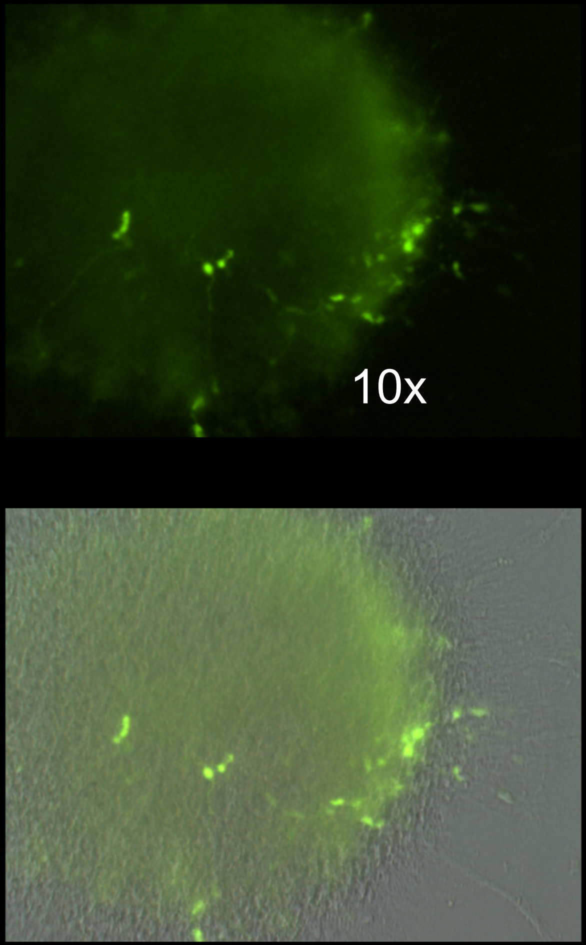

PBD_PEX1ms1 iPS1-derived

Motor neurons transfected with HB9 driven GFP

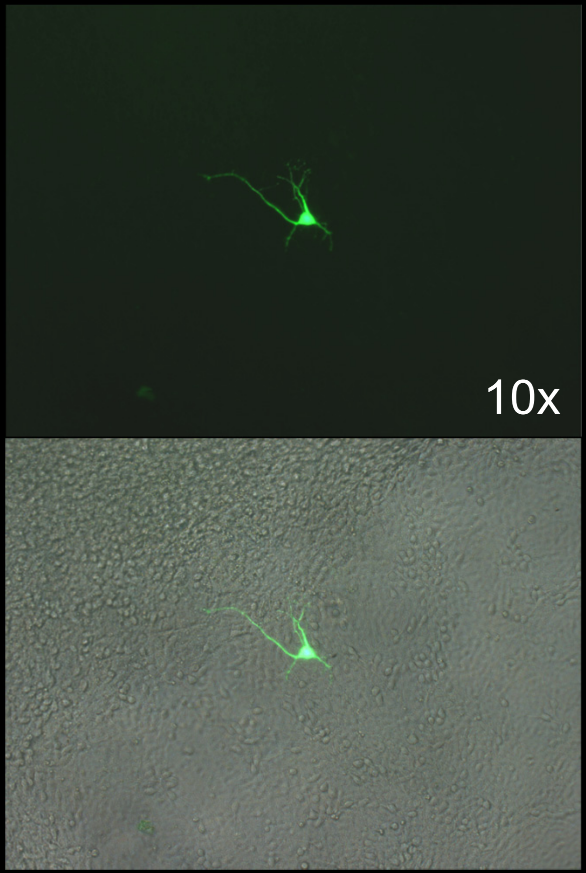

PBD_PEX1ms1 iPS2-derived

Motor neurons transfected with HB9 driven GFP

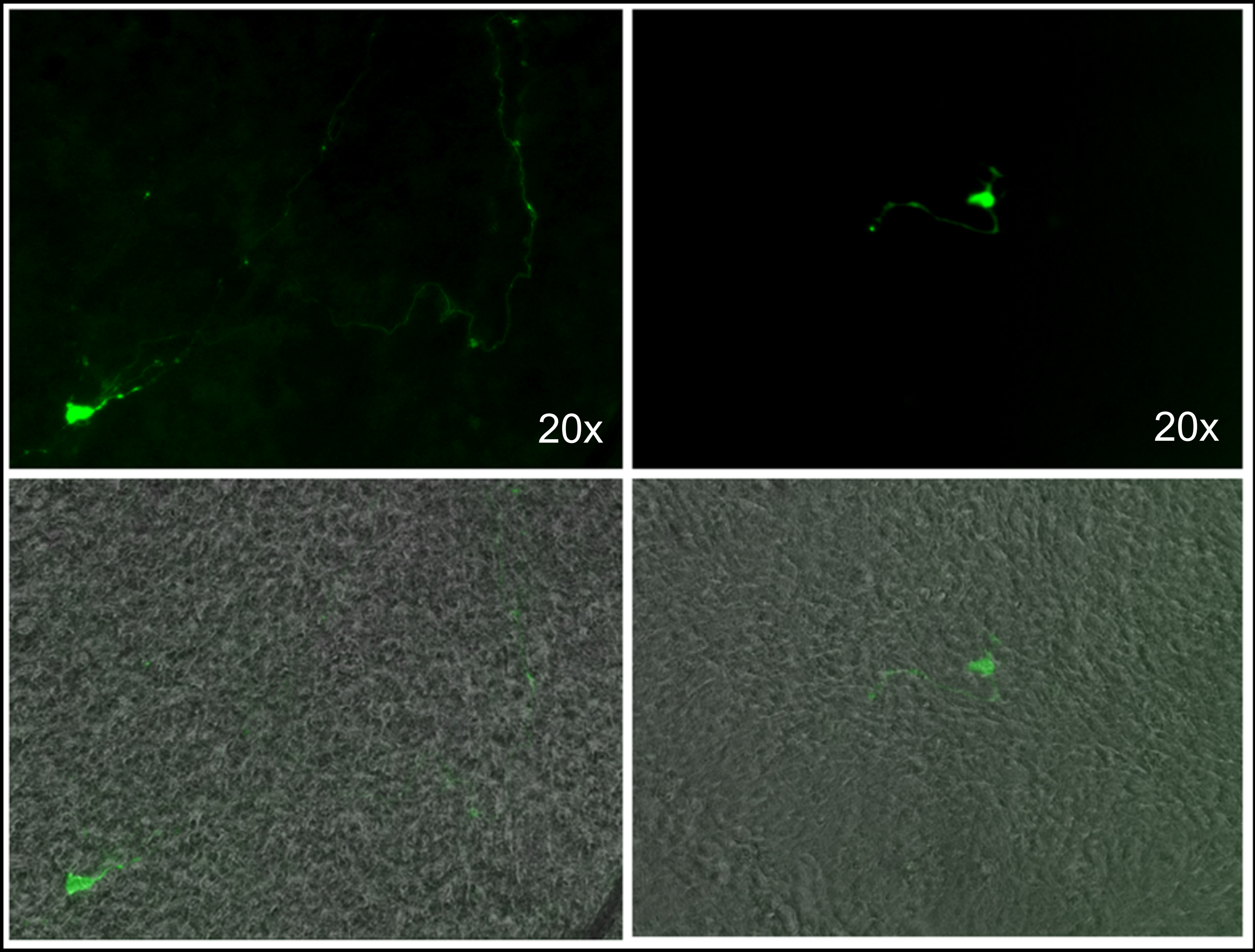

PBD_PEX1 ms1 iPS5-derived
